# Ms1 RNA Interacts With the RNA Polymerase Core in *Streptomyces coelicolor* and Was Identified in Majority of *Actinobacteria* Using a Linguistic Gene Synteny Search

**DOI:** 10.3389/fmicb.2022.848536

**Published:** 2022-05-11

**Authors:** Viola Vaňková Hausnerová, Olga Marvalová, Michaela Šiková, Mahmoud Shoman, Jarmila Havelková, Milada Kambová, Martina Janoušková, Dilip Kumar, Petr Halada, Marek Schwarz, Libor Krásný, Jarmila Hnilicová, Josef Pánek

**Affiliations:** ^1^Laboratory of Microbial Genetics and Gene Expression, Institute of Microbiology of the Czech Academy of Sciences, Prague, Czechia; ^2^Laboratory of Structural Biology and Cell Signaling, Institute of Microbiology of the Czech Academy of Sciences, Vestec, Czechia; ^3^Laboratory of Bioinformatics, Institute of Microbiology of the Czech Academy of Sciences, Prague, Czechia

**Keywords:** sRNA, *Actinobacteria*, Ms1 RNA, *Streptomyces*, gene synteny, *Mycobacterium*, 6S RNA

## Abstract

Bacteria employ small non-coding RNAs (sRNAs) to regulate gene expression. Ms1 is an sRNA that binds to the RNA polymerase (RNAP) core and affects the intracellular level of this essential enzyme. Ms1 is structurally related to 6S RNA that binds to a different form of RNAP, the holoenzyme bearing the primary sigma factor. 6S RNAs are widespread in the bacterial kingdom except for the industrially and medicinally important *Actinobacteria*. While Ms1 RNA was identified in *Mycobacterium*, it is not clear whether Ms1 RNA is present also in other *Actinobacteria* species. Here, using a computational search based on secondary structure similarities combined with a linguistic gene synteny approach, we identified Ms1 RNA in *Streptomyces*. In *S. coelicolor*, Ms1 RNA overlaps with the previously annotated scr3559 sRNA with an unknown function. We experimentally confirmed that Ms1 RNA/scr3559 associates with the RNAP core without the primary sigma factor HrdB *in vivo*. Subsequently, we applied the computational approach to other *Actinobacteria* and identified Ms1 RNA candidates in 824 *Actinobacteria* species, revealing Ms1 RNA as a widespread class of RNAP binding sRNAs, and demonstrating the ability of our multifactorial computational approach to identify weakly conserved sRNAs in evolutionarily distant genomes.

## Introduction

Small non-coding RNAs (sRNAs) are important regulators of gene expression in bacteria. A majority of sRNAs act by base-pairing to target mRNAs and change mRNA stability or translation but a minor group of sRNAs directly regulates proteins through RNA-protein interaction (Svensson and Sharma, 2016). The best-known example is 6S RNA that interacts with the RNA polymerase (RNAP) holoenzyme (Wassarman and Storz, 2000; Klocko and Wassarman, 2009; Steuten et al., 2013; Chen et al., 2017; Wassarman, 2018). The RNAP holoenzyme is composed of the catalytic core (E, subunits α_2_ββ′ω) and the primary σ factor that is required to recognize promoters of housekeeping genes to initiate transcription. All bacteria contain this housekeeping or primary σ factor, termed σ^70^ in *Escherichia coli*, σ^A^ in *Bacillus subtilis* and in *Mycobacterium smegmatis* (Gomez et al., 1998), or HrdB in *Streptomyces* (Brown et al., 1992). In addition, bacterial species contain different numbers of alternative sigma factors ranging from zero in *Mycoplasma genitalium* (Fraser et al., 1995) to almost 70 in *Streptomyces coelicolor* (Bentley et al., 2002).

6S RNA was first described in *E. coli* (Hindley, 1967) where it interacts with the RNAP-σ^70^ holoenzyme (Eσ^70^) (Wassarman and Storz, 2000) and regulates the expression of hundreds of genes (Cavanagh et al., 2008; Neusser et al., 2010). 6S RNA itself also serves as a template for transcription of pRNA, a short RNA of ≤20 nucleotides (Wassarman and Saecker, 2006; Beckmann et al., 2011; Hoch et al., 2016). Transcription of pRNA rearranges the structure of 6S RNA, releasing Eσ from 6S RNA (Wurm et al., 2010; Beckmann et al., 2012; Cavanagh et al., 2012; Panchapakesan and Unrau, 2012; Burenina et al., 2014).

6S RNA has a conserved secondary structure that is critical for the interaction with Eσ^70^ (Barrick et al., 2005; Trotochaud and Wassarman, 2005; Shephard et al., 2010). 6S RNA forms a double-stranded hairpin like structure with a central, unpaired bubble region which resembles an open promoter (Barrick et al., 2005; Wassarman, 2018). Many 6S RNAs were predicted from the genomic sequences based on secondary structure similarities (Barrick et al., 2005; Trotochaud and Wassarman, 2005; Wehner et al., 2014). Alternatively, 6S RNAs were directly identified as abundant ∼180–200 nt RNAs in *B. subtilis*, *Bordetella pertussis*, *Pseudomonas aeruginosa* or *Caulobacter crescentus* (Vogel et al., 1987; Barrick et al., 2005; Trotochaud and Wassarman, 2005) or 6S RNAs were discovered after the detection of their complementary pRNAs (Sharma et al., 2010; Köhler et al., 2015).

The intracellular levels of 6S RNAs are high, similar to those of essential non-coding RNAs, such as rRNAs, tRNAs, RNAse P, tmRNA or SRP RNA. Furthermore, 6S RNAs have been found in many bacterial species and are widespread in the bacterial kingdom (Trotochaud and Wassarman, 2005; Faucher et al., 2010; Sharma et al., 2010; Rediger et al., 2012; Wehner et al., 2014; Köhler et al., 2015; Jones et al., 2016; Elkina et al., 2017; Burenina et al., 2020) with one exception—the group of *Actinobacteria*. This phylum includes serious human pathogens (*Mycobacterium tuberculosis*, *Mycobacterium leprae*, and *Corynebacterium diphtheria*), industrially important producers of amino acids (*Corynebacterium glutamicum*) and antibiotics (*Streptomyces)*, bacteria involved in symbiotic nitrogen fixation (*Frankia*), bacteria utilized for bioremediation (*Rhodococcus*), probiotic bacteria (*Bifidobacterium*), and numerous other genera (*Nocardia*, *Micrococcus*, *Gardnerella*).

In *Actinobacteria*, 6S RNAs had been undetected for a long time. In *Mycobacterium smegmatis*, we identified a putative 6S RNA candidate by the computational suboptimal secondary structure approach (Pánek et al., 2011). However, we discovered that it interacted with the RNAP core (E) without the primary sigma factor (Hnilicova et al., 2014) and, thus, by definition, was not a 6S RNA and we therefore named it Ms1. In *Mycobacterium smegmatis*, Ms1 accumulates during the stationary phase of growth, regulates the RNAP level, and this facilitates cell outgrowth from stationary phase (Sikova et al., 2019).

Ms1 RNA is longer than a typical 6S RNA (∼300 nt vs. ∼180 nt) and their secondary structures differ as well—in addition to the central bubble, Ms1 has two additional short hairpins at its 3′ and 5′ ends (Hnilicova et al., 2014). An Ms1 homolog was also found in *M. tuberculosis* (MTS2823 sRNA) (Arnvig et al., 2011). Other Ms1 homologs were identified only in closely related species within the *Actinobacteria* group (genera *Mycobacterium*, *Rhodococcus* and *Nocardia*) (Hnilicova et al., 2014; Behra et al., 2019). Currently, it is unclear whether Ms1 RNA is present only in the three above-mentioned bacterial genera or is widespread throughout *Actinobacteria*.

In *Streptomyces coelicolor*, a putative 6S RNA (the gene was named *ssrS*) was identified (Panek et al., 2008), but the experimental proof that *ssrS* encodes 6S RNA was based on an *in vitro* interaction between the purified RNAP-HrdB holoenzyme and *in vitro* transcribed 6S RNA (Mikulík et al., 2014). In addition, this interaction was detected only after UV crosslinking (Mikulík et al., 2014). Moreover, *in vitro* transcribed *ssrS* gene only partially overlaps with the *in vivo* detected scr3559 sRNA identified by RNA-seq (Vockenhuber et al., 2011; Moody et al., 2013) which is expressed from the same genomic locus (*sco3558-sco3559* intergenic region) and thus it is unclear if the putative 6S RNA sequence is expressed *in vivo*.

Recently, (Bobek et al., 2021) reported a new 6S RNA in *S. coelicolor*, which was named “6S-like scr3559 RNA.” It is expressed from the *sco3558-sco3559* genomic locus but from the minus strand (the previously published 6S RNA sequence was expressed from the plus strand), with its transcription start site located at position 3,934,888 in the genome (Bobek et al., 2021). This 6S-like RNA is processed and its 5′ end corresponds to position 3,934,820 (Bobek et al., 2021). However, the processed 6S-like RNA was almost undetectable in wild type *S. coelicolor* by Northern blotting (Bobek et al., 2021). In addition, the 6S-like RNA does not correspond to the scr3559 sRNA that is transcribed from the plus strand with the transcription start site at position 3,934,693 (Moody et al., 2013; Jeong et al., 2016). Both sequences of the putative 6S RNAs (Mikulík et al., 2014; Bobek et al., 2021) differ from scr3559, although scr3559 is the main transcript derived from the *sco3558-sco3559* intergenic region as detected by RNA-seq (Vockenhuber et al., 2011; Moody et al., 2013). The function of scr3559 is unknown.

To elucidate whether Ms1 RNA is conserved in the phylogenetic group of *Actinobacteria*, we combined a homology search based on evolutionary conservation of RNAs with a bioinformatic linguistic search for genomic context (synteny) of RNA genes to identify Ms1 candidate genes. First, we applied this approach to *Streptomyces coelicolor*. Our search identified a Ms1 candidate—the scr3559 sRNA. We validated this result experimentally, demonstrating that scr3559 RNA binds the RNAP core without the primary σ factor HrdB *in vivo* and, thus, is an Ms1 but not a 6S RNA homolog. Subsequently, we applied our linguistic search to the phylum of *Actinobacteria*, revealing that Ms1 is present in most orders of *Actinobacteria*. Ms1 is thus a new type of regulatory RNA associated with RNA polymerase, in addition to 6S RNA, B2 RNA (regulates RNA polymerase II in humans) (Allen et al., 2004; Espinoza et al., 2004), or svRNAs (regulates the Influenza A virus RNA-dependent RNA polymerase) (Perez et al., 2010; Perez et al., 2012).

## Materials and Methods

### Computational Homology Search for New sRNAs

For the search, 10 selected *Streptomyces* well annotated genomes were used (GenBank assembly IDs shown in parenthesis following organism name): *S. coelicolor* A3(2) (GCA_000203835.1_ASM20383v1), *S. ambofaciens* ATCC 23877 (GCA_001267885.1_ASM126788v1), *S. nodosus* (GCA_000819545.1_ASM81954 v1), *S. reticuli* (GCA_001511815.1_TUE45), *S. avermitilis* MA 4680 (GCA_000009765.2_ASM976v2), *S. griseus* NBRC (GCA_000010605.1_ASM1060v1), *S. scabies* 87 22 (GCA_000091305.1_ ASM9130v1), *S. cattleya* NRRL8057 (GCA_000237305.1_ASM 23730v1), *S. lincolnensis* (GCA_001685355.1_ASM168535v1, *S. pristinaespiralis* ATCC 25486 (GCA_000154945.1_ASM 15494v1).

The search was performed in the following steps:

(i) Intergenic regions (IGRs) were identified in the 10 selected *Streptomyces* genomes, according to GenBank genomic annotations. IGRs in both DNA strands were searched, hence, an IGR in one strand may overlap with an open reading frame encoded by the other strand.
(ii) The IGR sequences were sampled with approximate nucleotide lengths of 180 and 300 nucleotides that are specific for 6S and Ms1 RNAs, respectively. The sampling was done in a sliding window, moving sequence window by 5 nucleotides a step through IGR sequences.
(iii) 10 suboptimal secondary structures were predicted for each sampled sequence by UNAfold (Markham and Zuker, 2008) with parameters *P* = 1000, *W* = 1 and *X* = 10.
(iv) The suboptimal structures were matched to secondary structures of *B. subtilis* 6S RNA (Trotochaud and Wassarman, 2005; Beckmann et al., 2012; Burenina et al., 2014) (for 6S RNA-length sequences) and *M. smegmatis* Ms1 RNA (Hnilicova et al., 2014) (for Ms1 RNA-length sequences) that were used as structural templates to get their pairwise structural similarity scores using RNAdistance (Lorenz et al., 2011).
(v) The average of three best scores for each sampled sequence was compared to the structural similarity thresholds of 80 and 135 for 6S RNA and Ms1 RNA, respectively. The thresholds were chosen such that we were able to identify known 6S and Ms1 RNAs by the search. To be considered as a candidate for one of the searched for sRNAs, the candidate sequence had to have the averaged similarity score of three most similar suboptimal structures better than the thresholds.

To increase reliability of the search, the candidates had to fulfill the following additional criteria derived from genomic properties of known 6S and Ms1 RNAs that were: (1) existence of similar sequences in evolutionarily close species identified by BLAST with BLAST E-values < 10^–20^, and (2) conserved genomic position in the ten searched *Streptomyces* genomes.

### Computational Prediction of Full Sequences of New *Streptomyces* sRNAs

**FIGURE 1:**
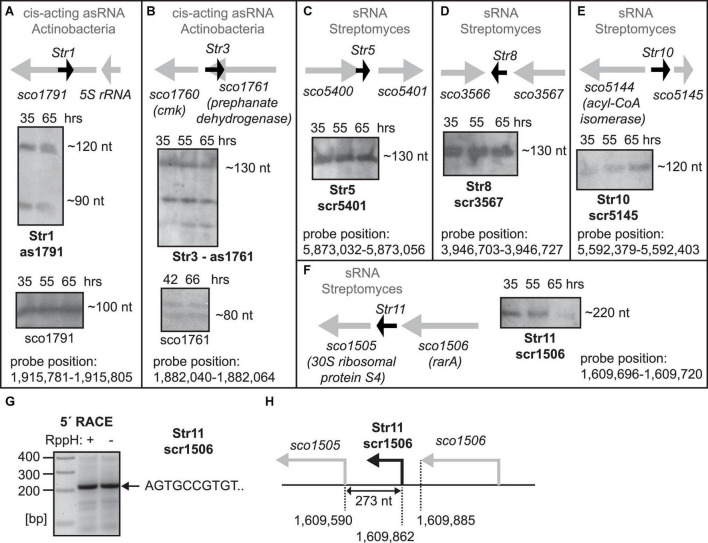
**(A–F)** Expression of predicted sRNAs. Total RNA was isolated from *S. coelicolor* at 35 (exponential phase of growth), 55 and 65 h after germination and the expression was detected by Northern blotting. 65 hours of growth represents stationary phase of growth. Orientation and the flanking genes are schematically shown for each sRNA. For as1791 and as1761 RNAs, fragments of sco1791 and sco1761 transcripts detected by Northern blotting are also shown in **(A,B)**. For scr1506, 5′ ends was detected by 5′ RACE **(G,H)**.

The full-length sequences were predicted using the approximate length of the transcripts detected by Northern blotting and the genomic positions of oligonucleotide sequences used for sRNA verification by Northern blotting (Figure 1). The sequences of approximate length were constructed using the oligonucleotides positioned at the 5′ end, center, and 3′ end. The three constructed sequences were BLASTed against genomes of the ten *Streptomyces* species listed in the previous paragraph. As sRNAs in general are conserved in evolutionarily related species, here in *Streptomyces* species, the constructed sequences had to be identified by BLAST in multiple *Streptomyces* species to ensure that they were constructed correctly. Using the superposition of constructed sequences according to their conservation identified by BLAST, we reconstructed probable sRNA sequences in *Streptomyces coelicolor*. The probable sequences are shown in Supplementary Table 1 for expressed *Streptomyces* sRNAs.

### Linguistic Search for Ms1 RNAs in *Actinobacteria*

The search was based on text phrases in genomic annotations of the genes flanking potential Ms1 RNA genes and sequence similarity search based on BLAST for verification. The search was possible as sRNAs including Ms1 RNA are known to be conserved in evolutionarily close bacteria (Hnilicova et al., 2014). The search has several steps:

(i) Synteny analysis. Specific words in annotations of 5′ and 3′ end flanking genes of known and newly discovered IGR containing Ms1 RNAs are identified. Among them, most repeating specific words are identified. This step is explained in detail in the following section.
(ii) Synteny phrases. Text phrases, comprising of at least one most repeating specific word, that are specific for genomic annotation of genes flanking Ms1 RNAs are generated. Synteny phrases used in this work are shown in Tables 1–3.
(iii) Text search for Ms1 synteny phrases in genomic annotations of *Actinobacteria* genera which had >4 annotated genomes and in which Ms1 RNA has not yet been discovered. The search is implemented as a sequence of grep LINUX searches, followed by additional text processing implemented in MATLAB computational environment.
(iv) Synteny hit. An IGR with Ms1 synteny containing a putative Ms1 RNA gene.
(v) Evolutionary conservation of the synteny hit. The hit was considered as evolutionarily conserved when there existed IGRs with similar sequence and Ms1 synteny for at least one of the flanking genes.
  The IGRs with similar sequences were identified by BLAST of the sequence of the synteny hit in NCBI’s nt database with sensitive setting for cross-species exploration (with parameters -r 1 -q 1 -G 1 -E 2 -W 7 (Korf et al., 2003) with BLAST E-value threshold set to 0.05).
  Annotations of flanking genes of the identified IGRs were checked for Ms1 synteny phrases. The annotations contained either Ms1 phrases, or their synonyms, or contained new phrases. When contained new phrases, only one of the flanking genes was allowed to contain them and at the same time they had to repeat in annotations of multiple IGRs. Both new phrases and synonyms were used to update old phrases for the next synteny search iteration (Figure 5).
  If the synteny hit was not found to be evolutionarily conserved, it was not used.
(vi) Flanking genes annotations of both synteny hit and IGRs homologous to it in new, related species. They were analyzed to get their specific words [step (ii)] and to update old synteny phrases [step (iii)].

**TABLE 1:** Synteny phrases for linguistic search for Ms1 RNA in *Streptomyces*.

‘oxidoreductase’	‘*inhibition morphological differentiation’*
	‘*IB HAD*’
	‘*HAD hydrolase*’
	‘*phosphoserine phosphatase*’

*The phrases were extracted from synteny annotations of Mycobacteria Ms1 RNA homologs (Supplementary Figure 1). Columns contains phrases for individual flanking genes. Rows indicate pairing of phrases for both flanking genes. Cells contain semantic synonyms (different phrases for the same protein function). Italics indicates functional synonyms (phrases denoting different aspects of proteins with the same function).*

**TABLE 2:** Synteny phrases for linguistic search after 1st update using synteny annotations of *Streptomyces* Ms1 RNA homologs (Supplementary Figure 2). For description of the table, see legend of Table 1.

**‘beta acetylhexosaminidase’** **‘beta glycosyl glucosidase’** **‘glycoside hydrolase’**	‘*inhibition morphological differentiation’*
‘oxidoreductase’	‘*IB HAD*’
	‘*HAD hydrolase*’
‘Fic’	‘*phosphoserine phosphatase*’
	‘CpaE’

*Here, semantic synonyms are either underlined or in italics or in bold. Note, that specific words were used to text search in a case-sensitive manner. Higher diversity of the phrases than in Table 1 was given by higher phylogenetic diversity of species bringing a higher diversity of annotations.*

**TABLE 3:** Synteny phrases for linguistic search after 2nd update using synteny annotations of *Cellulomonas* Ms1 RNA homologs (Supplementary Figure 3).

**‘beta acetylhexosaminidase’** **‘beta glycosyl glucosidase’** **‘glycoside hydrolase’**	‘*inhibition morphological differentiation’*
‘oxidoreductase’	‘*IB HAD*’
‘PH’	‘*HAD hydrolase*’
‘transcriptional regulator’	‘*phosphoserine phosphatase*’
	
‘Fic’	‘CpaE’

	‘chromosome partitioning’

*For description of the table, see legend of Tables 1, 2.*

**FIGURE 2:**
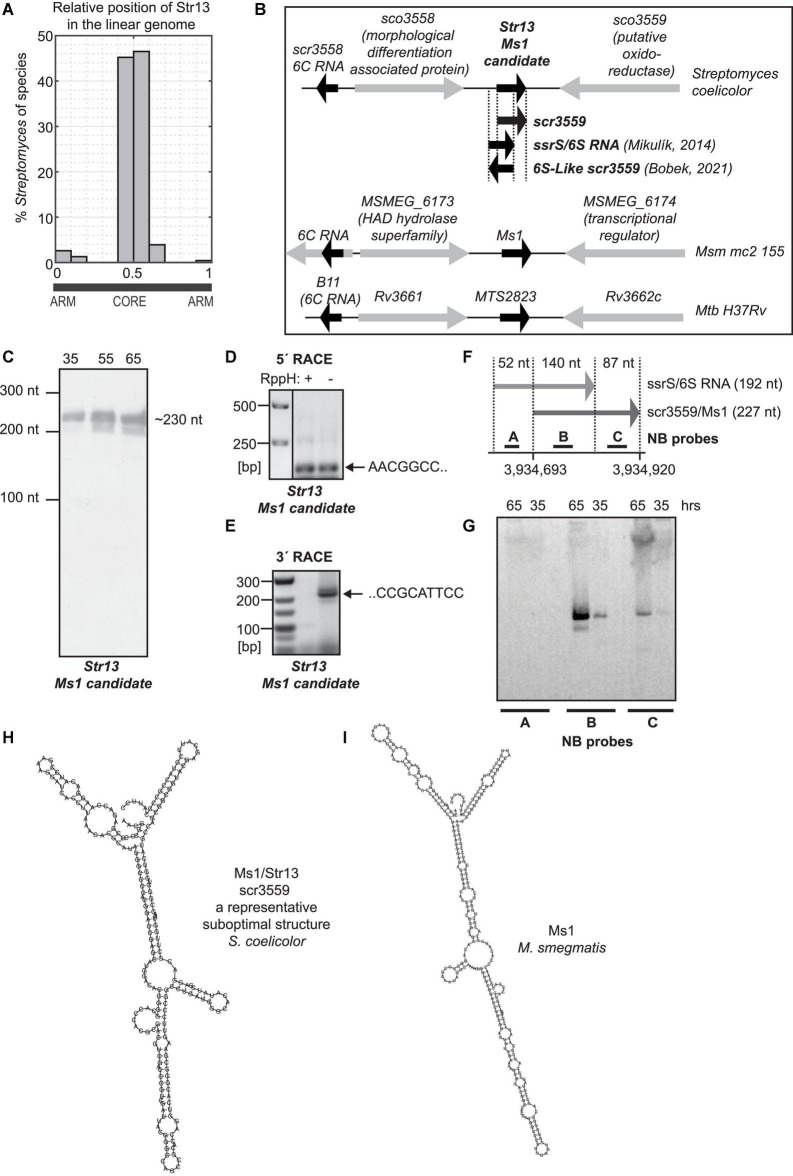
*S. coelicolor* Ms1 RNA candidate. (A) Histogram of relative genomic position of a Ms1 candidate, Str13, in 188 *Streptomyces* species. *x*-axis shows a relative genomic position with “0” and “1” corresponding to the terminal arms of the linear *Streptomyces* genome and with “0.5” corresponding to the middle of *Streptomyces* linear genome. *y*-axis shows percentage of 188 *Streptomyces* species in which Str13 homologs were identified using sequence similarity. The figure indicates that the Str13 relative genomic position is conserved in the middle of the linear *Streptomyces* genomes. (B) Ms1 candidate in *S. coelicolor*, Ms1 in *M. smegmatis* and MTS2823 Ms1 homolog in *M. tuberculosis* and their flanking genes. In *Streptomyces coelicolor*, positions of the previously published sRNAs are also included. The position of 6S-Like scr3559 was adopted from the 6S-Like scr3559 sequence reported in Figure 2B of Bobek et al. (2021). The position of *ssrS*/6S RNA was adopted from Mikulík et al. (2014), from the sequences of primers that were used to generate DNA template carrying T7 promoter for *in vitro* transcription of 6S RNA. Experiments showing 6S RNA—RNAP interaction were performed with this *in vitro* transcribed RNA. scr3559 position was adopted form available *S. coelicolor* dRNA-seq data (Romero et al., 2014; Jeong et al., 2016; Kim et al., 2020) and RNA-seq data (Vockenhuber et al., 2011; Moody et al., 2013). (C) ∼230 nt long RNA was detected by Northern blotting with the probe specific to Ms1 candidate/scr3559/(probe 2796). The 5′ end of Ms1 candidate was determined by 5′ RACE (D), 3′ end by 3′ RACE (E) and corresponds to the scr3559 sRNA (F). 6S RNA expression was not detected by Northern blotting (G). (H) Structure of scr3559. (I) Structure of Ms1 RNA from *M. smegmatis*.

**FIGURE 3:**
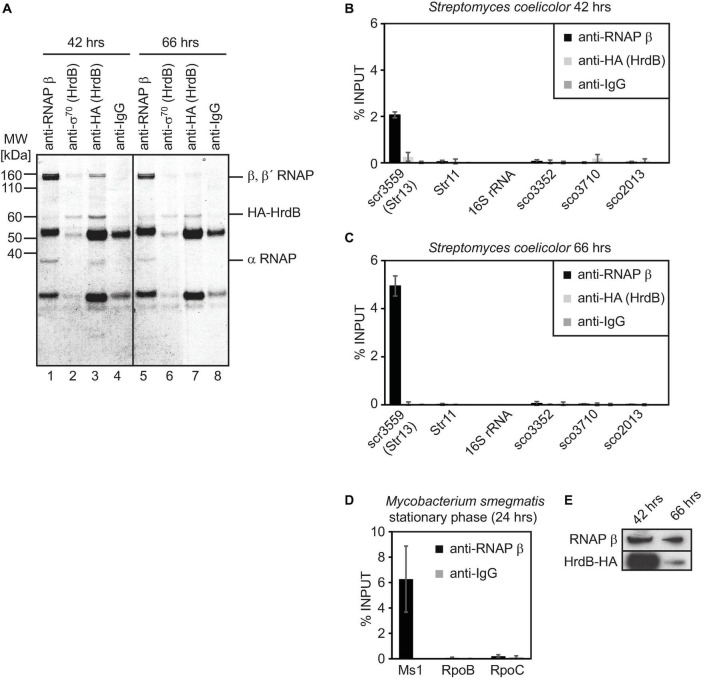
Immunoprecipitation of *S. coelicolor* RNAP β and HA-HrdB (A–C) and *M. smegmatis* RNAP β (D). Lysates from *S. coelicolor* cells carrying HA-tagged HrdB (42 and 66 h after germination, exponential and stationary phase of growth, respectively) were incubated with antibodies against RNAP β, sigma 70 and HA tag and immunoprecipitated proteins were resolved on SDS PAGE and stained with Coomassie (A). RNA that co-immunoprecipitation with RNAP or HrdB was isolated, cDNA was reverse transcribed and the amount of Ms1 and 6S RNA candidates were determined by qRT-PCR (B,C). In *S. coelicolor*, 16S rRNA and RNAs expressed from sco3352, sco3710, and sco2713 genes were selected as controls that should not bind to RNAP/HrdB. In *M. smegmatis*, the amount of Ms1 associated with RNAP is shown as a positive control, RpoB and RpoC mRNAs do not co-immunoprecipitate with RNAP (D). The error bars show ±SEM from at least three independent experiments. The amount of RNAP β and HrdB was measured by western blotting in *A3(2) hrdB-HA* 42 h (exponential phase) and 66 h after germination (stationary phase) (E), the same amount of proteins (15 μg) was loaded.

**FIGURE 4:**
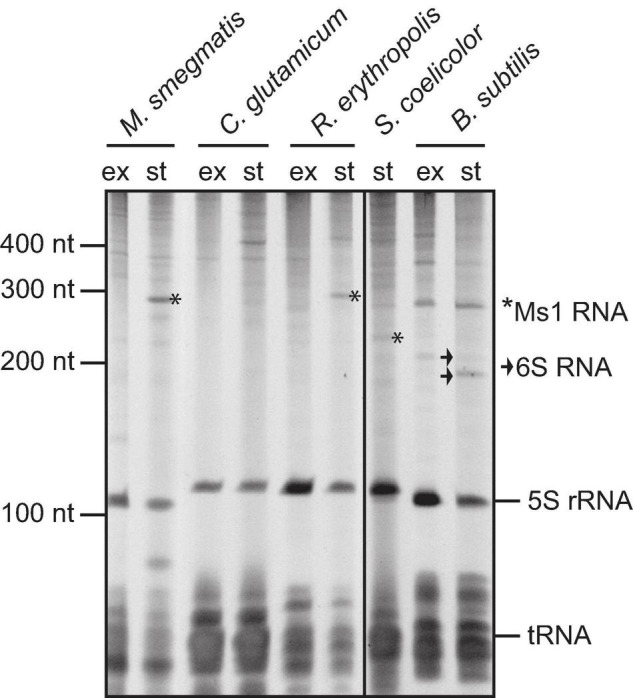
Total RNA isolated from *Mycobacterium smegmatis* mc^2^ 155, *Corynebacterium glutamicum*, *Rhodococcus erythropolis* CCM2595, *Streptomyces coelicolor* A3(2), and *Bacillus subtilis* from exponential (“ex”) and stationary (“st”) phase of growth, resolved on polyacrylamide gel electrophoresis and stained with GelRed. Ms1 RNAs in *M. smegmatis* and *R. erythropolis* and a band corresponding to the size of Str13/Ms1 in *S. coelicolor* are labelled with *. Two forms of *B. subtilis* 6S-1 RNA are marked by arrow.

**FIGURE 5:**
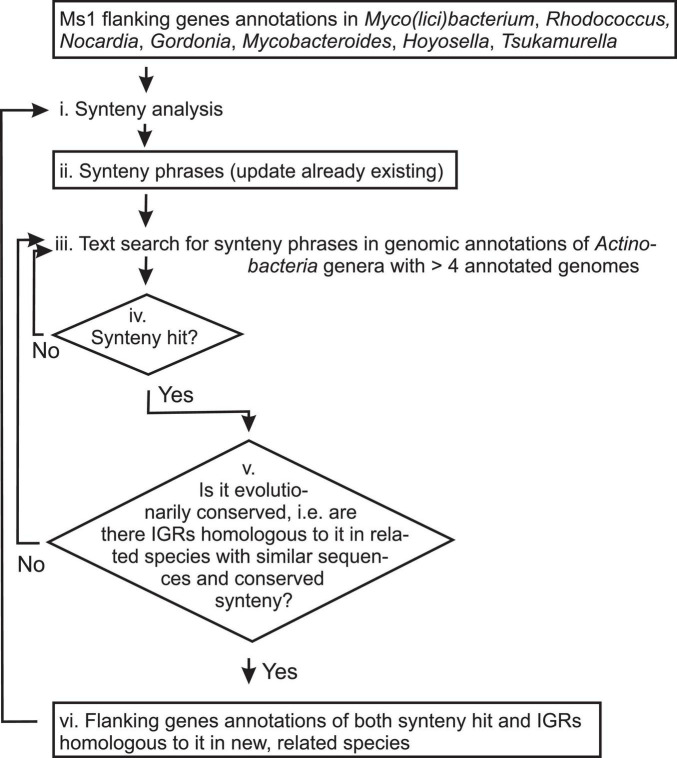
Scheme of the linguistic gene synteny search for Ms1 RNA in *Actinobacteria*. Titles of analyzed data and information are in boxes, while analytic steps are shown as plain text. Arrows indicate the data and information flow. Synteny analysis (step i.) generates Synteny phrases (Tables 1–3, step ii.). For the first synteny analysis, annotations of Ms1 flanking genes from *Mycobacterium, Rhodococcus, Nocardia*, *Gordonia, Mycobacteroides, Hoyosella*, and *Tsukamurella* were used and Table 1 generated. Synteny phrases are used to search for new putative Ms1 candidates (iv. Synteny hits). If the putative Ms1 candidates are evolutionary conserved in related species (step v.), annotations of their flanking genes (step vi.) are added to the Synteny analysis (step i.) to generate updated Synteny phrases (step ii., Tables 2, 3) and the whole procedure is repeated.

### Synteny Analysis

Annotations of flanking genes were first split into single words and non-specific words were removed. The non-specific words were (allowing for grammar errors): ‘hypothetical’, ‘protein’, ‘type’, ‘family’, ‘domain’, ‘putative’, ‘precursor’, ‘component’, ‘subunit’, ‘subfamily’, ‘conserved’, ‘chain’, ‘or’, ‘superfamily’, ‘unknown’, ‘function’, ‘of’, ‘like’, ‘containing’, ‘II’, ‘and’, ‘short’, ‘dependent’, ‘probable’, ‘associated’, ‘to’, ‘that’, ‘the’, ‘predicted’, ‘uncharacterized’, ‘production’, ‘proteases’, ‘fold’, ‘in’, ‘by’, ‘universal’, ‘pathway’, ‘involved’, ‘related’, ‘general’, ‘group’, ‘sequence’, ‘class’, ‘cluster’, ‘accepting’, and ‘determining’.

We also removed the so-called questionable non-specific words whose (non)specificity depended on the analyzed context. They were: ‘box’, ‘enzyme’, ‘factor’, ‘secreted’, ‘release’, ‘neighborhood’, ‘solute’, ‘accessory’, ‘peptide’, ‘biosynthesis’, and ‘substrate’.

Specific words were left. They characterized specific aspects of gene function. An example is the ‘Fic/Doc family protein’ annotation contained two non-specific words, ‘family’ and ‘protein’, and one specific word ‘Fic/Doc’.

The first synteny phrases were obtained from annotations of homologs of *M. smegmatis* Ms1 RNA identified by sequence similarity by BLAST in 498 species of 8 genera—*Mycobacterium*, *Mycolicibacterium*, *Rhodococcus, Nocardia*, *Gordonia*, *Mycobacteroides*, *Hoyosella*, and *Tsukamurella* (Supplementary Figure 1). Specific words were selected as those occurring in annotations of flanking genes above the average (Supplementary Figure 1). The specific words were ‘oxidoreductase’, ‘hydrolase’, ‘HAD’, ‘IB’, ‘morphological’, ‘differentiation’, ‘inhibition’, ‘phosphoserine’ and ‘phosphatase’. The occurrence in genera was used rather than the occurrence in species as there were genera with many species with repeating annotations with repeating specific words causing bias of an occurrence of certain words.

Phrases were generated from the most frequent specific words according to their co-occurrence in the annotations from which they were extracted and which defined their semantic binding. For example, specific words ‘hydrolase’, ‘HAD’, ‘IB’, ‘morphological’, ‘differentiation’, ‘inhibition’, ‘phosphoserine’ and ‘phosphatase’ (identified in Supplementary Figure 1 as most occurring) formed the following phrases: ‘IB HAD’, ‘HAD hydrolase’, ‘inhibition morphological differentiation’ and ‘phosphoserine phosphatase’. A complete list of first phrases is in Table 1.

Synteny phrases were repeatedly updated after identification of Ms1-containing IGRs in new species (Supplementary Figures 2–4). New annotations either contained synonyms to old phrases, e.g., oxidoreductase, whose synonym was Fic that also has oxidoreductase activity or contained new annotation phrases. The synonyms were identified using information found on the internet, most often in Wikipedia and/or many various public protein databases/knowledgebases. New annotations were considered as Ms1-syntenous when they repeated for one flanking gene of newly identified Ms1 IGRs in many species, while annotation of the other flanking gene must have contained the known Ms1 phrase. New annotations were analyzed to get new phrases out of them that were merged with old phrases as can be seen in Tables 2, 3.

### Bacterial Strains, Growth Conditions

*Mycobacterium smegmatis* mc^2^ 155 cells ATCC 700084 (*wt*, *LK865*) were grown at 37°C in Middlebrook 7H9 medium with 0.2% glycerol and 0.05% Tween 80, and harvested in exponential (OD_600_ ∼0.5) or early stationary phase (OD_600_ ∼2.5–3, 24 h of cultivation). *S. coelicolor A3*(*2*) spore stock expressing HA-tagged HrdB was thawed and inoculated to 2YT medium. Germination was carried out at 30°C for 5 h as described previously (Moody et al., 2013; Šmídová et al., 2019). The germinated spores were harvested by centrifugation (3200 × *g*, 25°C, 5 min), inoculated into Na-glutamate medium supplemented with trace element solution and TMS1 as described previously (Šmídová et al., 2019), cultivated at 30°C and harvested by centrifugation (3200 × *g*, 4°C, 5 min) at different time points after germination. *Rhodococcus erythropolis* CCM259 (Strnad et al., 2014) (*LK1556*) was cultivated at 26°C in 2x TY medium to exponential phase (OD_600_ ∼2) or stationary phase (OD_600_ ∼11, 24 h of cultivation), *Corynebacterium glutamicum* ATCC 13032 (*LK1557*) was grown in 2xTY at 30°C and harvested in exponential (OD_600_ ∼1) or stationary phase (OD_600_ ∼8, 24 h of cultivation). *Bacillus subtilis* 168 strain was grown in LB medium at 37°C to exponential phase (OD_600_ ∼0.3) or stationary phase (OD_600_ ∼4).

### RNA Isolation and Staining, Northern Blotting

Each frozen cell pellet was resuspended in 240 μl TE (pH 8.0) plus 60 μl LETS buffer (50 mM Tris–HCl pH 8.0, 500 mM LiCl, 50 mM EDTA pH 8.0, 5% SDS) and 600 μl acidic phenol (pH∼3):chloroform (1:1). Lysates were sonicated in a fume hood, centrifuged, the aqueous phase extracted three more times with acidic phenol (pH∼3): chloroform and precipitated with ethanol. RNA was dissolved in water, treated with DNase (TURBO DNA-free Kit, Ambion) and visualized on a 7 M urea 7% polyacrylamide gel by staining with GelRed (Labmark).

RNAs were resolved on a 7% polyacrylamide gel and transferred onto an Amersham Hybond-N membrane according to the protocol described in Pánek et al. (2011). 5′ biotinylated oligonucleotide probes were hybridized to the membrane and detected with BrightStar BioDetect Kit (Ambion) according to manufacturer’s instructions. For Northern blot probes sequences, see the Supplementary Material.

### 5′ RACE

The protocol was adopted from Martini et al. (2019). An adapter oligo CTGGAGCACGAGGACACTGACATGGACTG AAGGAGTagaaa (lower case letters are ribonucleotides, upper case letter deoxyribonucleotides) was ligated to RNA 5′ ends. 5 μg DNase-treated RNA was treated with RppH (NEB). Treated and untreated RNA samples (8 μl) were mixed with 1 μl of 1 μg/μl adapter oligo and incubated at 65°C for 10 min, then ligation reaction was set up including 10 μl 50% PEG8000, 3 μl 10X ligase buffer, 3 μl 10 mM ATP, 3 μl DMSO, 1 μl Murine RNase inhibitor (NEB), and 1 μl T4 ligase (NEB). Ligation was performed at 20°C overnight and RNA cleaned using RNA Clean and Concentrator 25 kit (Zymo). RNA was reverse-transcribed into cDNA (SuperScript III, Invitrogen) with random hexamers. PCR was done using a forward primer CTGGAGCACGAGGACACTGA and reverse (gene specific) primers (Str11, 5′-AGCCGCTCCCCTGGTCTGGG-3′, Ms1 candidate 5′-GGTGTCCATGCTCGGTCC-3′). The nucleotide position of the TSS was taken from EMBL/GenBank Accession No. AL645882.

### 3′ RACE

The protocol was adopted from Sedlyarova et al. (2017). 550 pmol of 5′-phosphorylated RNA adaptor (5′P -AAUGGACUCGUAUCACACCCGACAA-3′) was ligated to 6 μg of total DNase treated RNA using T4 RNA Ligase 1 (ssRNA Ligase, New England Biolabs) according to the manufacturer’s protocol overnight at 16°C. RNA was purified with RNA Clean and Concentrator 25 kit (Zymo) and reverse-transcribed into cDNA (SuperScript III, Invitrogen) with 3′ RACE specific primer (5′-TTGTCGGGTGTGATACGAGTCCATT-3′). The same primer was used as reverse primer for PCR together with gene specific forward primer (5′- GATCACCTTAAACACGCATATGG-3′).

### Immunoprecipitation and RT-qPCR

*Streptomyces coelicolor* cells expressing HA-tagged HrdB were pelleted and resuspended in lysis buffer (20 mM Tris–HCl pH 7.9, 150 mM KCl, 1 mM MgCl_2_, 1 mM dithiothreitol (DTT), 0.5 mM phenylmethylsulfonyl fluoride (PMSF), supplemented with Calbiochem Protease Inhibitor Cocktail Set III protease inhibitors), sonicated 15 × 10 s with 1 min pauses on ice and centrifuged. 500 μg (protein) of lysates were incubated for 16–18 h at 4°C with 20 μl of Protein G plus agarose beads (Santa Cruz) coated with 5 μg mouse monoclonal anti-β subunit of RNAP antibody [clone 8RB13] (BioLegend), 2.5 μg of anti-σ^70^ antibody [clone 2G10] (BioLegend), 1.25 μg of anti-HA antibody [clone HA-7] (Sigma-Aldrich) or 5 μg of mouse non-specific IgG (Sigma-Aldrich) used as a negative control, respectively. The captured complexes were washed 4 times using 20 mM Tris–HCl pH 7.9, 150 mM KCl, 1 mM MgCl_2_, finally resuspended in 300 μl 20 mM Tris–HCl pH 7.9, 150 mM KCl, 1 mM MgCl_2_ and divided into two parts. One third of the beads were incubated in SDS sample buffer for 5 min at 95°C and eluted proteins were detected by Coomassie staining and Western blotting. The remaining two thirds of the beads were resuspended in 200 μl 1% SDS, 150 mM KCl, 20 mM Tris–HCl pH 7.9, 1 mM MgCl_2_ and incubated on a rotating platform with 200 μl acidic phenol (pH∼3):chloroform (1:1) for 15 min. Eluted RNA was precipitated with ethanol, dissolved in water and DNase treated (TURBO DNA-free Kit, Ambion). RNA was reverse transcribed into cDNA (SuperScript III, Invitrogen) using random hexamers and amplified by quantitative reverse transcription PCR (RT-qPCR) in a LightCycler 480 System (Roche Applied Science) in duplicate reactions containing LightCycler 480 SYBR Green I Master and 0.5 μM primers (each). Primers were designed with Primer3 software and their sequences are in the Supplementary primer list. Negative controls (no template reactions and reactions with RNA as a template to control for contamination with genomic DNA) were run in each experiment, the quality of the PCR products was determined by dissociation curve analysis, and the efficiency of the primers determined by standard curves. The relative amounts of co-immunoprecipitated RNAs were quantified on the basis of the threshold cycle (Ct) for each PCR product that was normalized to input values according to the formula 2^∧^[Ct(immunoprec)–Ct(input)].

### Western Blotting

Proteins were detected by Western blotting using a rat monoclonal antibody recognizing the HA tag conjugated with HRP [clone BMG-3F10] or a mouse monoclonal antibody recognizing β subunit of RNA polymerase [clone 8RB13] in a combination with secondary antibody conjugated with HRP.

## Results

### Computational Search for 6S RNA and Ms1 RNA Candidates in *Streptomyces*

First, we conducted a computational homology search for putative 6S RNA/Ms1 RNA in the *Streptomyces* genus.

Using this computational search, we identified 12 candidate genes for 6S/Ms1 RNAs in *Streptomyces* (see Supplementary Material). Interestingly, flanking genes of one of them (Str11, Supplementary Table 1) displayed annotations syntenous to 6S-1 RNA in *Firmicutes* (Wehner et al., 2014), making it a prime candidate for *Streptomyces* 6S RNA. Nevertheless, none of the putative identified sRNA genes had the synteny of mycobacterial Ms1 RNAs. We suspected that the search might have not identified all candidate genes (for reasons see section “Discussion”). Therefore, prior to the experimental validation, we extended the homology search with linguistic synteny analysis approach.

The linguistic approach was based on search for text phrases that were specific to Ms1 RNA synteny and could be found in Ms1 flanking genes annotations. Note that meaning of the term ‘synteny’ in this work is ‘conserved genomic context’. Synteny annotations are annotations of conserved flanking genes of Ms1 RNAs. The phrases were used to identify new IGRs containing putative Ms1 RNAs in other species.

Synteny was rarely employed previously for identification of sRNAs (Sridhar and Gunasekaran, 2013), mostly because the sRNAs and their synteny occurred only in certain phyla, e.g., in *Enterobacteriaceae* (Sridhar and Rafi, 2007) and were not widespread in bacteria. Nevertheless, 6S RNA is an example of a sRNA identified throughout the bacterial kingdom with synteny conserved in specific taxons—for example in *Enterobacteriaceae* (γ-*Proteobacteria*) (Wehner et al., 2014). Although no 6S RNA syntenic pattern is valid for all bacteria, some proteins frequently occur in the syntenic regions of the 6S RNA throughout the bacterial kingdom. For example, *ygfA*, which encodes 5-formyltetrahydrofolate cyclo-ligase, is found adjacent to 6S RNA gene in α-, γ- *Proteobacteria*, and some species from β-*Proteobacteria*, δ-*Proteobacteria*, or *Firmicutes* (Barrick et al., 2005; Wehner et al., 2014). Therefore, as Ms1 and 6S RNAs are structurally and functionally similar, we assumed that Ms1 flanking genes would be at least partially conserved in *Actinobacteria* similarly to 6S RNA in γ-*Proteobacteria*.

Annotations of flanking genes of Ms1 RNAs identified previously in *Mycobacterium*, *Rhodococcus*, and *Nocardia* were conserved (Hnilicova et al., 2014). We speculated that the conservation would also be kept in those *Actinobacteria* where Ms1 RNA had not been identified. To verify this assumption, we first identified homologs of *M. smegmatis* Ms1 RNA by sequence similarity using BLAST in 498 species of 8 genera and analyzed annotations of their flanking genes. As expected, we found it conserved (Supplementary Figure 1). Therefore, we extracted synteny text phrases (Table 1) from the annotations and used them to search genomic annotations for their occurrence indicating putative Ms1 RNAs.

Intergenic regions with flanking genes with Ms1 RNA-specific synteny phrases (synteny hits) were found in numerous *Streptomyces* species for both ‘HAD hydrolase’ and ‘inhibition morphological differentiation’ phrases paired with the ‘oxidoreductase’ phrase. For example, in the first of the *Streptomyces* species, *S. actuosus*, a total of six hits of ‘HAD hydrolase’ were obtained. Only one of them fulfilled the other criteria of the synteny search, which was Ms1 synteny phrase (‘oxidoreductase’) in annotation of the other flanking gene and the length of IGR between flanking genes larger than 300 nucleotides. This IGR was considered as an IGR containing a putative *Streptomyces* Ms1 RNA.

The next criterion was an evolutionary conservation of the candidate Ms1 IGR in related species, i.e., in other *Streptomyces* species, analogously to Ms1 RNA from *M. smegmatis* conserved in other *Mycobacteria*. Therefore, the sequence of the *S. actuosus* Ms1 IGR was BLASTed against the NCBI nt database, which produced ∼250 BLAST hits in 188 different *Streptomyces* species (Supplementary Figure 6) with E-values < 1 × 10^–24^, i.e., strong sequence similarity and with a similar position in the middle of the linear *Streptomyces* chromosome (Figure 2A). Both sequence similarity and similar genomic loci indicated evolutionary conservation thus suggesting that the IGRs contained *Streptomyces* Ms1 RNAs.

Among the BLAST hits, a 419 nucleotides long IGR in *S. coelicolor* A3(2) (ENA ID AL645882.2) were identified at genomic locus 3934559: 3934978. To find out where within this IGR a putative Ms1 RNA was, the 419 nt sequence was sampled with 200–300 nt subsequences in 5 nt steps for which suboptimal secondary structures were predicted using UNAfold (Markham and Zuker, 2008) and compared to the secondary structure of *M. smegmatis* Ms1 RNA used as a structural template. This way we aimed at identification of a subsequence of the IGR able to adopt a Ms1 RNA-like secondary structure, thus identifying a local position of Ms1 RNA. The Ms1 RNA-like secondary structures were obtained with 220–235 nt subsequences at positions 126–141 downstream of 5′ end of the IGR sequence. Note that this sequence of the putative *S. coelicolor* Ms1 RNA had no similarity detectable by cross-species exploration BLAST to the sequence Ms1 RNA from *M. smegmatis*.

To summarize this part, we identified a total of 13 (12 + 1) potential 6S/Ms1 RNA candidate genes in *S. coelicolor*.

### Expression of Potential 6S/Ms1 RNAs

Next, we used Northern analysis to determine expression of these putative sRNA, probing their expression from both strands. The analysis revealed that several of them were expressed, to various degrees, in exponential and stationary phases in *S. coelicolor* (Figures 1A–F, 2C). Expression of the remaining six sRNAs was not detected. Genomic loci of the new sRNAs are depicted in Figure 1 and their basic characteristics are described in detail in the next two sections and summarized in Supplementary Table 1 (including predicted sequence, position in the *S. coelicolor* genome, annotations of flanking genes, and location at the chromosome in the *Streptomyces* genus).

### Characterization of the Expressed sRNAs: Str1, Str3, Str5, Str8, and Str10

Str1 sRNA is an antisense RNA (as1791, Figure 1A) to the tetratricopeptide repeat protein gene (*sco1791*) and it was found in two forms (90 and 120 nt). Additionally, for Str1 we detected a short transcript (100 nt) from the opposite strand, a fragment of the sco1791 (1260 nt) mRNA.

Str3 localizes to the *sco1761* gene and similarly to Str1, Str3, is also an antisense RNA (130 nt, AS1711, Figure 1B). Furthermore, we also detected two short transcripts (110 and 80 nt, respectively) from the opposite strand, fragments of the 1086 nt long sco1761 mRNA. The antisense nature of both Str1 and Str3 sRNAs suggests that these sRNAs might be *cis*-acting antisense RNAs and the detected transcripts are fragments of the respective regulated mRNAs.

Str5 (scr5401, Figure 1C), Str8 (scr3567, Figure 1D), and Str10 (scr5145, Figure 1E) were expressed from intergenic regions and their lengths ranged from 120 to 130 nt.

We concluded that the identified genes encode bona fide sRNAs that are expressed in *S. coelicolor* but their short length (<150 nt) excluded them as potential 6S/Ms1 RNAs. 6S RNA/Ms1 RNA must adopt specific secondary structures and one of the shortest known 6S RNAs is from *Aquifex aeolicus*, which is ∼160 nt long (Köhler et al., 2015).

### Characterization of the Expressed sRNAs: Str11 and Str13

Str11 (scr1506), the candidate with the same synteny as 6S-1 RNA in *B. subtilis*, was long enough (∼220 nt long, Figure 1F) to be considered as a 6S RNA candidate. We identified the exact Str11 5′ end by 5′ RACE (Figures 1G,H). Str11 also had a 6S-like predicted consensus secondary structure (Supplementary Figure 5).

Str13, the Ms1 candidate identified by the linguistics search, partially overlaps with the previously discovered *ssrS* gene (Panek et al., 2008), which was proposed to encode a 192 nt long 6S RNA (Mikulík et al., 2014). The Str13 sequence also overlaps with the scr3559 sRNA identified by RNA-seq (Vockenhuber et al., 2011; Moody et al., 2013). In *S. coelicolor*, both *scr3559* and *ssrS* are located between the *sco3558* and *sco3559* genes (Vockenhuber et al., 2011; Moody et al., 2013). The flanking genes and positions of Str13 in *S. coelicolor* and Ms1 in *M. smegmatis* and MTS2823 in *M. tuberculosis* are shown in Figure 2B; Arnvig et al. (2011), Hnilicova et al. (2014).

To start deciphering whether Str13 (Ms1 candidate) or ssrS (putative 6S RNA) is expressed from the *sco3558* - *sco3559* intergenic region, we performed Northern blot analysis. We used a probe that could hybridize to both Str13 and ssrS/6S RNA and we detected a signal that corresponded to a ∼230 nt RNA (Figure 2C). This could represent the previously reported 244 nt long ssrS/6S RNA unprocessed transcript (Mikulík et al., 2014). Although we also detected a shorter transcript (Figure 2C), the major isoform was the ∼230 nt long RNA and not the 192 nt ssrS/6S RNA. The size of the RNA detected by the Northern blot (∼230 nt) corresponded to the previously published lengths of scr3559: 235 bp (Vockenhuber et al., 2011) or 227 bp, respectively (Moody et al., 2013).

Next, we mapped the 5′ and 3′ ends of Str13 by 5′ RACE. The 5′ end was identified at position 3,934,693 (Figure 2D) that is 52 nucleotides downstream from the 5′end of the previously annotated ssrS/6S RNA (Mikulík et al., 2014; Figure 2F) and 134 nt downstream from 5′ end of IGR. This position agrees with the computationally predicted genomic locus of the putative *S. coelicolor* Ms1 (Str13) RNA - predicted Ms1 starts 126–141 nt downstream from 5′ end of IGR. This 5′ RACE result matches the 5′ end of scr3559 as determined by RNA-seq (Vockenhuber et al., 2011; Moody et al., 2013) and dRNA-seq (Romero et al., 2014; Jeong et al., 2016; Kim et al., 2020) in *S. coelicolor*. We also searched for additional transcription start sites in previously published data and found position 3,933,713 (Kim et al., 2020) that was 20 nucleotides upstream of the 5′ end of scr3559 but also did not correspond to the 5′ end of ssrS/6S RNA that is 52 nucleotides upstream. The 3′ end of Str13 was then determined by 3′ RACE (Figure 2E) at the position 3,934,920, which corresponds to the 3′ end of scr3559 (Vockenhuber et al., 2011; Moody et al., 2013) but not ssrS/6S RNA. The 3′ end in the same position (3,934,920) was identified also by Term-seq (Lee et al., 2020).

To determine whether scr3559, along with ssrS/6S RNA is perhaps expressed, we used three different probes for Northern blot analysis. Probe “A” should specifically hybridize to ssrS/6S RNA, probe “B” to both sr3559 and ssrS/6S RNA, and probe “C” only to the sr3559 (Figure 2F). We detected the ∼230 nt band only with probes B and C that hybridized to sr3559 (Figure 2G). No signal specific for ssrS/6S RNA was detected at 35 and 65 h after germination, indicating that the putative 6S RNA (*ssrS* gene) is not expressed in detectable amounts in these growth phases.

We cannot exclude that ssrS/6S RNA is expressed under unknown conditions but the main sRNA transcript derived from the *sco3558-sco3559* genomic locus starts at position 3,934,693 and differs from the ssrS/6S RNA sequence that was used to experimentally test the interaction of the putative 6S RNA with the RNAP-HrdB holoenzyme *in vitro* (Mikulík et al., 2014).

As we identified the full sequence of the Ms1 candidate by 5′ and 3′ RACE, we used suboptimal structure folding to search for Ms1-like secondary structure motifs. We folded the Ms1 candidate sequence by UNAfold (with parameters *P* = 5000, *W* = 2 and *X* = 100) that predicted 78 suboptimal structures. The structures were clustered into 5 clusters based on their mutual structure similarity to find structurally representative folds. The clusters represented structural variations of a typical Ms1 fold. The most representative fold was identified in a cluster with most mutually similar suboptimal structures that contained typical Ms1-like structures which resembled Ms1 from *M. smegmatis* (Figure 2I), revealing its potential to interact with RNAP. An example of the secondary structure from that cluster is shown in Figure 2H.

We concluded that both Str11 (scr1506) and Str13 (scr3559) satisfied the criteria for potential 6S/Ms1 candidates and we selected them for further analysis.

### Str13 (scr3559) Binds the RNAP Core *in vivo*

To answer whether Str11 (scr1505) and/or Str13 (scr3559) are homologs of 6S RNAs or Ms1, we wanted to immunoprecipitate the primary σ factor, HrdB, and RNAP from *S. coelicolor.* As the commercially available antibody against the primary σ^70^ (clone 2G10) interacted with HrdB only weakly (Figure 3A, lanes 2 and 6), we used a strain with an HA-tagged *S. coelicolor hrdB* gene (Šmídová et al., 2019) and immunoprecipitated HA-HrdB with an anti-HA antibody from exponentially growing (42 h post-germination) and stationary (66 h post-germination) cells. The anti-HA antibody pulled down HA-HrdB and α, β, β′ subunits of RNAP, especially at 42 h post-germination (Figure 3A, lane 3, protein band identities were verified by mass spectrometry). Thus, the anti-HA antibody interacted both with HA-HrdB alone and also with the RNAP-HrdB complex, which binds 6S RNA in many bacterial species. Then, we immunoprecipitated RNAP with the antibody against the RNAP β subunit (Figure 3A lanes 1 and 5). This antibody preferentially recognizes the RNAP core without the primary σ factor (Figure 3A). We subsequently isolated co-immunoprecipitated RNAs and measured their relative amounts by RT-qPCR (Figures 3B,C).

Str11 (scr1505) associated neither with the RNAP core nor with the RNAP-HrdB holoenzyme, similar to four control RNAs that also did not interact with RNAP: 16S rRNA, sco3552, sco3710 encoding membrane proteins, and sco2013 encoding response regulator PdtaR.

Importantly, ∼2% of Str13 (scr3559) was bound to the RNAP core at 42 h post-germination in *S. coelicolor* (Figure 3B, the input represents the total amount of scr3559 isolated from the cell lysates), and it increased to ∼5% at 66 h post-germination (Figure 3C). scr3559 bound neither HrdB alone nor the HrdB-RNAP complex. As a control, we performed immunoprecipitation with the same antibody from stationary phase *M. smegmatis* cells and ∼6% of Ms1 co-immunoprecipitated with the RNAP core (Figure 3D). As we noticed that only a low amount of HrdB-HA was immunoprecipitated at 66 h post-germination (Figure 3A, lane 7) compared to 42 h (Figure 3A, lane 3), we compared the relative levels of HrdB-HA and RNAP in *S. coelicolor*. The amount of HrdB-HA significantly decreased at 66 h post-germination compared to 42 h (Figure 3E) while the level of the RNAP β subunit was almost unchanged. This suggests that the level of HrdB and subsequently, the level of the RNAP-HrdB complex is low in the late phase of growth in *S. coelicolor*, similar to previous observations in *M. smegmatis* (Hnilicova et al., 2014). Str13 (scr3559) was associated *in vivo* with the RNAP core but not with the HrdB-RNAP holoenzyme.

Therefore, we concluded that Str11 (scr1505) is neither 6S RNA nor Ms1 but a sRNA of unknown function. To the contrary, Str13 (scr3559) is a bona fide homolog of Ms1 in *S. coelicolor* and we propose to rename Str13 (scr3559) as Ms1.

### Relative Amounts of Ms1 in Selected *Actinobacteria* Species

In *Mycobacterium smegmatis* and *Rhodococcus erythropolis*, Ms1 is an abundant RNA in stationary phase, prominently visible in the gel, similarly to 6S RNA in *Bacillus subtilis* (Figure 4). In *Streptomyces coelicolor*, we detected a weak ∼230 nt long RNA visible in stationary phase RNA, which might be Ms1 RNA identified in this study. However, in *Corynebacterium glutamicum*, a species that is relatively evolutionarily close to *Mycobacteria* (both are in one order - *Corynebacteriales*), there are no prominent bands in the ∼200–300 nt range, suggesting that it might not contain an Ms1 RNA or its expression is below the detection limit of the staining. Therefore, we decided to extend the linguistic search to the whole group of *Actinobacteria* to reveal how widespread Ms1 RNA is within other *Actinobacteria* species.

### Linguistic Search for Ms1 RNA in *Actinobacteria*

After we identified *S. coelicolor* Ms1 RNA using the linguistic search, we applied it to other *Actinobacteria*. A flowchart of the search procedure is shown in Figure 5.

We started with the synteny phrase identified in *Mycobacterium, Rhodococcus, Nocardia*, *Gordonia, Mycobacteroides, Hoyosella*, and *Tsukamurella* (Table 1, step ii. in Figure 5) which had been used to discover Str13 (Ms1 homolog) in *Streptomyces coelicolor*.

For the search, *Actinobacteria* genera with more than four annotated species available in GenBank, according to NCBI Taxonomy (Schoch et al., 2020), were used. In total, there were 40 including *Streptomyces*. Synteny hits (step iv. in Figure 5) were obtained in five of them, namely in *Cellulomonas, Williamsia, Actinospicaceae, Actinopolyspora*, and *Streptomyces* (Str13). Note that sequences of the synteny hits, i.e., IGRs containing putative Ms1 RNAs, may be dissimilar to each other and therefore could not be identified by sequence similarity searches.

Based on sequence similarity to synteny hit from *Streptomyces actuosus*, we identified IGRs containing Ms1 in 158 species from 46 *Actinobacteria* genera other than *Streptomyces* (step v. in Figure 5). Based on occurrence of specific words from the annotations of the identified IGRs (Supplementary Figure 2 step vi. in Figure 5), we generated the new synteny phrases (step i. in Figure 5) and added them to the original phrases (this returned us to the step ii. in Figure 5 and new Table 2 was generated with updated synteny phrases).

Using the updated synteny phrases and the second iterative synteny search, a synteny hit in *Cellulomonas gilvus* was found. Based on sequence similarity, Ms1 IGRs in 708 species from 109 *Actinobacteria* genera were identified. Specific words and a histogram of their occurrence in synteny annotations of the 708 Ms1 IGRs are shown in Supplementary Figure 3. The subsequently updated synteny phrases are shown in Table 3.

The phrases in Table 3 yielded synteny hits into another three genera: *Williamsia*, *Actinopolyspora*, and *Actinospica* whose synteny annotations did not produce any new synteny phrases. Sequence similarity of the *Williamsia* synteny hit was limited to the *Williamsia* genus and sequence similarity of the both *Actinopolyspora* and *Actinospicaceae* synteny hits identified with mostly already known Ms1 RNA candidates in species of evolutionarily closed genera.

Interestingly, in *Catenulispora* (*Catenulisporales*), *Brevibacterium* (*Micrococcales*), *Corynebacterium* (*Corynebacteriales*), *Actinomyces* (*Actinomycetales*), and *Bifidobacterium* (*Bifidobacteriales*) we found no IGRs that could contain Ms1 RNA (Figure 5A, labeled in gray). We found IGRs with the Ms1 RNA synteny in the species of these taxonomic groups but they were too short to accommodate Ms1 RNA. For example, in *Corynebacterium bovis*, the Ms1-syntenous IGR was only 6 bps long and in species of the other groups there were Ms1 RNA-syntenous IGRs between only 20 and 100 nucleotides long.

**FIGURE 6:**
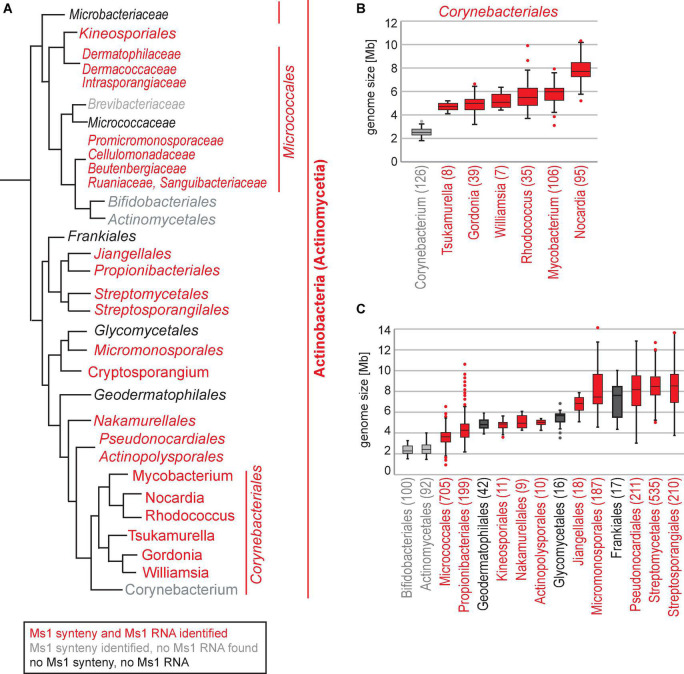
**(A)** Ms1 homologs were identified in *Actinobacteria* orders (in italics) or families (narrow italics) labeled by red. *Actinobacteria* groups with no identified Ms1 homologs and Ms1 flanking genes are in black, groups with identified Ms1 flanking genes but no Ms1 homologs in grey. The tree was adopted from Nouioui et al. (2018). **(B)** Genome sizes of the *Corynebacteriales* and **(C)** *Actinobacteria.* Only NCBI reference genomes are shown, number of genomes is indicated in brackets.

In summary, the linguistics gene synteny search identified Ms1 RNA homologs in 824 *Actinobacteria* species (Supplementary Table 2 and Supplementary Figure 5 for specific words in their synteny annotations) belonging to 146 genera and 14 *Actinobacteria* orders (Figure 6A, labeled in red and Table 4). Thus, Ms1 RNA is widespread among *Actinobacteria* and Ms1 RNA interaction with the RNA polymerase core is conserved both in *Mycobacterium smegmatis* and *Streptomyces coelicolor*.

**TABLE 4:** List of *Actinobacteria* orders, families, and genera with species with predicted Ms1 RNAs.

Order	Family	Genus
Actinopolysporales	Actinopolysporaceae	Actinopolyspora, Halopolyspora
Catenulisporales	Actinospicaceae	Actinospica, Actinocrinis
Corynebacteriales	Gordoniaceae	Gordonia
	Mycobacteriacea	Mycobacterium, Mycobacteroides, Mycolicibacterium
	Nocardiaceae	Nocardia, Rhodococcus, Williamsia
	Tsukamurellaceae	Tsukamurella
	Corynebacteriales incertae sedis	Fodinicola
Cryptosporangiales	Cryptosporangiaceae	Cryptosporangium
Sporichthyales	Sporichthyaceae	Sporichthya
Jiangellales	Jiangellaceae	Jiangella
Kineosporiales	Kineosporiaceae	Angustibacter, Kineosporia
Micrococcales	Beutenbergiaceae	Beutenbergia
	Cellulomonadaceae	Cellulomonas, Actinotalea, Oerskovia
	Dermacoccaceae	Allobranchiibius, Barrientosiimonas, Calidifontibacter, Demetria, Flexivirga, Leekyejoonella, Luteipulveratus, Metallococcus, Piscicoccus, Rudaeicoccus, Yimella
	Dermatophilaceae	Austwickia, Mobilicoccus
	Intrasporangiaceae	Humibacillus, Janibacter, Intrasporangium, Knoellia, Lapillicoccus, Ornithinicoccus, Oryzihumus, Pedococcus, Phycicoccus, Segeticoccus, Tetrasphaera
	Micrococcaceae	Arthrobacter, Ornithinimicrobium
	Ornithinimicrobiaceae	Ornithinimicrobium
	Promicromonosporaceae	Cellulosimicrobium, Isoptericola
	Ruaniaceae	Occultella, Ruania
	Sanguibacteriaceae	Sanguibacter
	Micrococcales incertae sedis	Luteimicrobium
Micromonosporales	Micromonosporaceae	Actinocatenispora, Actinoplanes, Allocatelliglobosispora, Allorhizocola, Asanoa, Catellatospora, Catelliglobosispora, Catenuloplanes, Couchioplanes, Dactylosporangium, Hamadaea, Krasilnikovia, Mangrovihabitans, Phytohabitans, Pilimelia, Planosporangium, Pseudosporangium, Rhizocola, Rugosimonospora, Spirilliplanes, Virgisporangium
Nakamurellales	Nakamurellaceae	Nakamurella
Propionibacteriales	Nocardioidaceae	Actinopolymorpha, Aeromicrobium, Kribbella, Nocardioides, Marmoricola, Pimelobacter
	Propionibacteriaceae	Auraticoccus, Friedmanniella, Microlunatus
Pseudonocardiales	Pseudonocardiaceae	Actinoalloteichus, Actinokineospora, Actinophytocola, Actinopolyspora, Actinosynnema, Alloactinosynnema, Amycolatopsis, Allokutzneria, Crossiella, Goodfellowiella, Haloechinothrix, Herbihabitans, Kibdelosporangium, Kutzneria, Labedaea, Lentzea, Longimycelium, Prauserella, Pseudonocardia, Saccharomonospora, Saccharopolyspora, Saccharothrix, Streptoalloteichus, Tamaricihabitans, Thermocrispum, Thermobispora, Umezawaea
Streptomycetales	Carbonactinosporaceae	Carbonactinospora
	Streptomycetaceae	Embleya, Kitasatospora, Streptomyces
Streptosporangiales	Nocardiopsaceae	Lipingzhangella, Marinactinospora, Marinitenerispora, Nocardiopsis, Spinactinospora, Streptomonospora, Thermobifida
	Streptosporangiaceae	Acrocarpospora, Bailinhaonella, Herbidospora, Microbispora, Microtetraspora, Non-omuraea, Planobispora, Planomonospora, Planotetraspora, Sinosporangium, Sphaerimonospora, Sphaerisporangium, Spongiactinospora, Streptosporangium, Thermoactinospora, Thermocatellispora, Thermopolyspora
	Thermomonosporaceae	Actinoallomurus, Actinocorallia, Actinomadura, Spirillospora, Thermomonospora, Thermostaphylospora

*One hit was obtained in unspecified species annotated as ‘Actinobacteria bacterium’ (not included in the table).*

## Discussion

The presented study reveals the ubiquitous presence of Ms1 RNA in *Actinobacteria* (exceptions might exist, see below), identifying this sRNA as a major class of protein-interacting RNAs. Ms1 RNA associates with the RNAP core as previously demonstrated in *Mycobacteria* (Hnilicova et al., 2014) and here in *Streptomyces* (Figure 3). In addition, our linguistic gene synteny search proved to be a potent tool to identify sequentially unrelated RNAs in evolutionarily distant species.

### Ms1 RNA Binds RNAP in *Streptomyces*

We bioinformatically identified Str13 as the Ms1 RNA candidate in *S. coelicolor*. Str13 overlaps with scr3559 sRNA (Figure 2B), an sRNA with unknown function. We showed that Str13/scr3559 is an Ms1 homolog in *S. coelicolor.* Str13/scr3559 had a similar predicted secondary structure with the mycobacterial Ms1 (cf. Figures 2H,I) and both RNAs bind the RNAP core (Figure 3). Ms1 RNA is thus conserved in evolutionarily distant and morphologically divergent *Mycobacterium* and *Streptomyces*. *Mycobacteria* are unicellular rod-shaped bacteria, while *Streptomyces* have a complex life cycle, which starts with the germination of a spore that prolongs into filamentous tubes of highly branched vegetative (primary) mycelium, then secondary mycelium is formed and eventually spores (Trotochaud and Wassarman, 2004, 2006; Faucher et al., 2010; Cavanagh and Wassarman, 2013; Hoch et al., 2015). Despite the completely different life cycles, both bacterial species have maintained Ms1 sRNA.

### Genomic Position of Ms1

In circular genomes of *Mycobacterium*, *Nocardia*, and *Rhodococcus*, Ms1 RNA is located close to the ori (replication start site) with the direction of transcription oriented toward it (Hnilicova et al., 2014). This is similar to Ms1 RNAs in *Streptomyces* that is positioned in the middle of the linear genome, close to ori (Figure 2A). The *Streptomyces* genomes have a core region of about 4.9 Mb containing essential genes and left and right arms with 1.5 Mb and 2.3 Mb, respectively (Hopwood, 2006), carrying mostly non-essential and species-specific genes. The position of Ms1 RNA genes in the core region indicates that this sRNA belongs among conserved genes in *Streptomyces*, consistent with our findings that identified Ms1 RNA candidates in 188 *Streptomyces* species (see Supplementary Figure 6). We further found Ms1 RNA in 145 other *Actinobacteria* genera (Table 4). Note that the number of newly identified Ms1 RNAs was limited by the availability of annotated *Actinobacteria* genomes as there were relatively many genera with only one or two genomes available or/and with a single species classified per genus.

### Ms1 and Genome Size

In some genera, such as *Corynebacterium, Bifidobacterium*, and *Actinomyces*, we detected the Ms1 synteny but the IGR was too short to accommodate Ms1. The missing Ms1 RNA-containing IGR in *Corynebacterium* was consistent with the absence of a prominent band in the 200–300 nt range in RNA gels from *Corynebacterium* (Figure 4). The *Corynebacterium* genus belongs to the *Corynebacteriales* order, which also includes *Mycobacterium*, *Nocardia*, *Rhodococcus* [where Ms1 RNA has been already described (Hnilicova et al., 2014)], *Williamsia, Gordonia*, and *Tsukamurella*, where we identified Ms1 RNAs using the linguistic gene synteny search (Figure 6A). Within the *Corynebacteriales* order, *Corynebacteria* have the smallest genome (Figure 6B). Ms1 thus might have been lost from *Corynebacterium* due to the evolutionary pressure to maintain a reduced genome. Alternatively, Ms1 RNA could be essential for *Actinobacteria* with the larger genomes.

A comparison of genome sizes of the main *Actinobacteria* orders with the occurrence of predicted Ms1 RNAs (Figures 6A,C) reveals a trend where Ms1 is lost in bacteria with smaller genomes while the Ms1 synteny is still present. Examples are *Bifidobacteriales* and *Actinomycetales* where the respective IGRs were too short to accommodate Ms1 RNA; these orders have the smallest genomes within *Actinobacteria* (mostly <3.0 Mbp).

However, in *Frankiales*, *Geodermatophilales*, and *Glycomycetales*, where neither the Ms1 synteny nor Ms1 RNA were detected, genome sizes were comparable to *Actinobacteria* orders with identified Ms1 RNAs. *Frankia* (*Frankiales*) genome sizes vary between 4.3 and 10 Mb and this variability is due to the degree of host dependence. *Frankia* are N_2_-fixing filamentous plant symbiotic bacteria that can either survive independently in the soil or be entirely dependent on their host plants (Benson et al., 2011). The high diversity of *Frankia* genomes might be a reason why Ms1 RNA was not detected in our search (the gene synteny on which our search depends, might be too low in *Frankia*). *Glycomycetales* are aerobic bacteria that produce branched vegetative mycelia and aerial mycelia with chains of square-ended conidia (Labeda and Kroppenstedt, 2004). *Glycomyces* were isolated from soil, hypersaline habitats, and seawater (Han et al., 2014; Xing et al., 2014; Nikitina et al., 2020). *Geodermatophilales* create pigmented (very often black) colonies with the individual cubic cells and extracellular matrix forming cauliflower-like aggregates and have been reported to be highly resistant to stresses such as gamma-radiation, UV, and desiccation (Hezbri et al., 2016). Species from both *Glycomycetales* and *Geodermatophilales* can adapt to extreme stress conditions and thus might have evolved specific regulatory pathways independent of Ms1 RNA.

To summarize this section, the genome size is not the only indicator of the Ms1 RNA presence. Alternatively, Ms1 RNA may be present in these species but the Ms1 RNA gene synteny was lost and therefore we were unable to detect Ms1 RNA using our gene-synteny based approach.

### Gene Linkage of Ms1 With HAD Hydrolase

The Ms1 RNA synteny itself, especially the “HAD hydrolase” gene is of interest. HAD hydrolase is annotated also as “inhibition of morphological differentiation protein” or “phosphoserine phosphatase” (see Tables 1–3) and it was found in the Ms1 RNA synteny of most species. In *Mycobacterium smegmatis*, HAD hydrolase is *MSMEG_6173*, a 293 amino acid long protein with a predicted transmembrane domain at its C-terminus. *SCO3558*, a 5′ end flanking gene of scr3559, that shares 60% identity with *MSMEG_6173*, is homologous to CicA (Bellier et al., 2006), which encodes a phosphotransferase in *Caulobacter crescentus*. An increased concentration of CicA in *Caulobacter crescentus* causes a loss of the normal rod shape, an almost 10-fold increase of the bacteria’s cell volume and a cell division block (Fuchs et al., 2001). As Ms1 regulates the RNAP amount in stationary phase (Sikova et al., 2019) it is tempting to speculate that the conserved association of the HAD hydrolase gene with the Ms1 RNA gene indicates a link between transcription regulation and cell size and shape.

### Computational Approaches Revisited

From the perspective of computational biology, our work demonstrated limits of the use of secondary structure in computational homology searches for homologs of known sRNAs in bacterial genomes. Homology searches use similarity of secondary structure between potential homologs and known sRNAs either solely [e.g., (Pánek et al., 2011)] or in combination with sequence similarity [e.g., (Barquist et al., 2016)]. Structure similarity increases the efficiency and capability of these searches to find homologs as the sRNA secondary structures are more evolutionarily conserved than sRNA sequences.

Nevertheless, still the efficiency of homology searches decreases substantially with the increasing evolutionary distance. This was demonstrated here in the extremely diverse group of *Actinobacteria* by the search for Ms1 RNA homologs, which was not successful. Also Rfam (Kalvari et al., 2017), an RNA database that employs the infernal software (Barquist et al., 2016) for computational search for homologs of known RNAs, provides Ms1 RNA candidates only from species closely related to *Mycobacteria*.

A weak point of homology searches is the limited reliability of secondary RNA structure prediction, which decreases especially for sequences >100 nt. Furthermore, a correct RNA sequence, i.e., a sequence with both a correct position in the genome and a correct length, is required for the prediction. But it is not always available in the homology search as (1) the length of the sequence can vary substantially between species or genera, and (2) genomic position of the sequence can be only approximated. In the presented work, the length difference between the known *M. smegmatis* Ms1 RNA and the identified *S. coelicolor* Ms1 RNA homolog was 70 nucleotides and therefore predicted secondary structures of potential *S. coelicolor* Ms1 RNA homologs were wrong and structure similarity to known Ms1 RNA could not be detected. Thus, the unavailability of correct sequences of potential homologs could be another reason why homology search was not successful in the presented work.

To overcome this problem, we employed a genomic synteny search. We adopted a linguistics approach based on similarity of genome annotations rather than similarity of nucleotide or amino acid sequences of flanking genes. Ms1 RNA has conserved synteny with a highly specific phrase in one of its flanking genes, the ‘HAD IB hydrolase’. This phrase is relatively rare and occurs only a few times in well annotated genomes (e.g., 5 × in *S. coelicolor*). The annotation helped us to identify the IGRs that might contain Ms1 RNA even in extremely distant species as represented here by the *Actinobacteria* genera with predicted Ms1 RNA, in which flanking nucleotide/amino acid sequences might be dissimilar.

We designed the most parsimonious form of a linguistic search for conserved synteny based on text searches for exact words or phrases. Once the search had identified the first possible Ms1 homolog in a genus (synteny hit), it then proceeded in an iteratively progressive manner within the genus and also in evolutionarily close genera using sequence similarity of the synteny hits. The obtained information was subsequently applied to other genera, expanding the list of identified candidates. Even in this simple form, the computational text search was able to identify Ms1 homologs in *Streptomyces* and other distantly related *Actinobacteria* genera. Its versatility and ease of use make it a convenient tool that can be, in principle, applied to searches for other RNAs/genes, allowing their fast identification across a wide range of organisms.

## Data Availability Statement

The original contributions presented in the study are included in the article/Supplementary Material, further inquiries can be directed to the corresponding authors.

## Author Contributions

OM, MK, DK, and MJ validated sRNAs expressions. MŠ and JH performed 5′ and 3′ RACE. VVH and MŠ performed immunoprecipitation experiments. PH identified proteins by mass spectrometry. JP and MSc performed the computations. MSc, LK, JHn, and JP wrote the manuscript. JHn and JP designed the study. All authors contributed to the article and approved the submitted version.

## Conflict of Interest

The authors declare that the research was conducted in the absence of any commercial or financial relationships that could be construed as a potential conflict of interest.

## Publisher’s Note

All claims expressed in this article are solely those of the authors and do not necessarily represent those of their affiliated organizations, or those of the publisher, the editors and the reviewers. Any product that may be evaluated in this article, or claim that may be made by its manufacturer, is not guaranteed or endorsed by the publisher.

## References

[B1] Allen T. A. Von Kaenel S. Goodrich J. A. Kugel J. F. (2004). The SINE-encoded mouse B2 RNA represses mRNA transcription in response to heat shock. *Nat. Struct. Mol. Biol.* 11 816–821. 10.1038/nsmb813 15300240

[B2] Arnvig K. B. Comas I. Thomson N. R. Houghton J. Boshoff H. I. Croucher N. J. (2011). Sequence-based analysis uncovers an abundance of non-coding RNA in the total transcriptome of *Mycobacterium tuberculosis*. *PLoS Pathog.* 7:e1002342. 10.1371/journal.ppat.1002342 22072964 PMC3207917

[B3] Barquist L. Burge S. W. Gardner P. P. (2016). Studying RNA homology and conservation with infernal: from single sequences to RNA families. *Curr. Protoc. Bioinformatics* 54 12.13.1–12.13.25. 10.1002/cpbi.4. 27322404 PMC5010141

[B4] Barrick J. E. Sudarsan N. Weinberg Z. Ruzzo W. L. Breaker R. R. (2005). 6S RNA is a widespread regulator of eubacterial RNA polymerase that resembles an open promoter. *RNA* 11 774–784. 10.1261/rna.7286705 15811922 PMC1370762

[B5] Beckmann B. M. Burenina O. Y. Hoch P. G. Kubareva E. A. Sharma C. M. Hartmann R. K. (2011). In vivo and in vitro analysis of 6S RNA-templated short transcripts in *Bacillus subtilis*. *RNA Biol.* 8 839–849. 10.4161/rna.8.5.16151 21881410

[B6] Beckmann B. M. Hoch P. G. Marz M. Willkomm D. K. Salas M. Hartmann R. K. (2012). A pRNA-induced structural rearrangement triggers 6S-1 RNA release from RNA polymerase in *Bacillus subtilis*. *EMBO J.* 31 1727–1738. 10.1038/emboj.2012.23 22333917 PMC3321203

[B7] Behra P. R. K. Pettersson B. M. F. Das S. Dasgupta S. Kirsebom L. A. (2019). Comparative genomics of *Mycobacterium mucogenicum* and *Mycobacterium neoaurum* clade members emphasizing tRNA and non-coding RNA. *BMC Evol. Biol.* 19:124. 10.1186/s12862-019-1447-7 31215393 PMC6582537

[B8] Bellier A. Gominet M. Mazodier P. (2006). Post-translational control of the *Streptomyces lividans* ClgR regulon by ClpP. *Microbiology* 152(Pt 4), 1021–1027. 10.1099/mic.0.28564-0 16549666

[B9] Benson D. R. Brooks J. M. Huang Y. Bickhart D. M. Mastronunzio J. E. (2011). The biology of Frankia sp. strains in the post-genome era. *Mol. Plant Microbe Interact.* 24 1310–1316. 10.1094/MPMI-06-11-0150 21848398

[B10] Bentley S. D. Chater K. F. Cerdeno-Tarraga A. M. Challis G. L. Thomson N. R. James K. D. (2002). Complete genome sequence of the model actinomycete *Streptomyces coelicolor* A3(2). *Nature* 417 141–147. 10.1038/417141a 12000953

[B11] Bobek J. Mikulová A. Šetinová D. Elliot M. Çihák M. (2021). 6S-Like scr3559 RNA affects development and antibiotic production in *Streptomyces coelicolor*. *Microorganisms* 9:2004. 10.3390/microorganisms9102004 34683325 PMC8539372

[B12] Brown K. L. Wood S. Buttner M. J. (1992). Isolation and characterization of the major vegetative RNA polymerase of *Streptomyces coelicolor* A3(2); renaturation of a sigma subunit using GroEL. *Mol. Microbiol.* 6 1133–1139. 10.1111/j.1365-2958.1992.tb01551.x 1350315

[B13] Burenina O. Y. Elkina D. A. Migur A. Y. Oretskaya T. S. Evguenieva-Hackenberg E. Hartmann R. K. (2020). Similarities and differences between 6S RNAs from *Bradyrhizobium japonicum* and *Sinorhizobium meliloti*. *J. Microbiol.* 58 945–956. 10.1007/s12275-020-0283-1 33125669

[B14] Burenina O. Y. Hoch P. G. Damm K. Salas M. Zatsepin T. S. Lechner M. (2014). Mechanistic comparison of *Bacillus subtilis* 6S-1 and 6S-2 RNAs–commonalities and differences. *RNA* 20 348–359. 10.1261/rna.042077.113 24464747 PMC3923129

[B15] Cavanagh A. T. Klocko A. D. Liu X. Wassarman K. M. (2008). Promoter specificity for 6S RNA regulation of transcription is determined by core promoter sequences and competition for region 4.2 of sigma70. *Mol. Microbiol.* 67 1242–1256. 10.1111/j.1365-2958.2008.06117.x 18208528

[B16] Cavanagh A. T. Sperger J. M. Wassarman K. M. (2012). Regulation of 6S RNA by pRNA synthesis is required for efficient recovery from stationary phase in E. coli and B. subtilis. *Nucleic Acids Res* 40 2234–2246. 10.1093/nar/gkr1003 22102588 PMC3299989

[B17] Cavanagh A. T. Wassarman K. M. (2013). 6S-1 RNA function leads to a delay in sporulation in *Bacillus subtilis*. *J. Bacteriol.* 195 2079–2086. 10.1128/JB.00050-13 23457253 PMC3624594

[B18] Chen J. Wassarman K. M. Feng S. Leon K. Feklistov A. Winkelman J. T. (2017). 6S RNA mimics B-form DNA to regulate *Escherichia coli* RNA polymerase. *Mol. Cell* 68 388–397.e6. 10.1016/j.molcel.2017.09.006 28988932 PMC5683422

[B19] Elkina D. Weber L. Lechner M. Burenina O. Weisert A. Kubareva E. (2017). 6S RNA in *Rhodobacter sphaeroides*: 6S RNA and pRNA transcript levels peak in late exponential phase and gene deletion causes a high salt stress phenotype. *RNA Biol.* 14 1627–1637. 10.1080/15476286.2017.1342933 28692405 PMC5785217

[B20] Espinoza C. A. Allen T. A. Hieb A. R. Kugel J. F. Goodrich J. A. (2004). B2 RNA binds directly to RNA polymerase II to repress transcript synthesis. *Nat. Struct. Mol. Biol.* 11 822–829. 10.1038/nsmb812 15300239

[B21] Faucher S. P. Friedlander G. Livny J. Margalit H. Shuman H. A. (2010). *Legionella pneumophila* 6S RNA optimizes intracellular multiplication. *Proc. Natl. Acad. Sci. U. S. A.* 107 7533–7538. 10.1073/pnas.0911764107 20368425 PMC2867745

[B22] Fraser C. M. Gocayne J. D. White O. Adams M. D. Clayton R. A. Fleischmann R. D. (1995). The minimal gene complement of *Mycoplasma genitalium*. *Science* 270 397–403. 10.1126/science.270.5235.397 7569993

[B23] Fuchs T. Wiget P. Osterås M. Jenal U. (2001). Precise amounts of a novel member of a phosphotransferase superfamily are essential for growth and normal morphology in *Caulobacter crescentus*. *Mol. Microbiol.* 39 679–692. 10.1046/j.1365-2958.2001.02238.x 11169108

[B24] Gomez M. Doukhan L. Nair G. Smith I. (1998). sigA is an essential gene in *Mycobacterium smegmatis*. *Mol. Microbiol.* 29 617–628. 10.1046/j.1365-2958.1998.00960.x 9720877

[B25] Han X. X. Luo X. X. Zhang L. L. (2014). *Glycomyces fuscus* sp. nov. and *Glycomyces albus* sp. nov., actinomycetes isolated from a hypersaline habitat. *Int. J. Syst. Evol. Microbiol.* 64(Pt 7), 2437–2441. 10.1099/ijs.0.061788-0 24776532

[B26] Hezbri K. Ghodhbane-Gtari F. Montero-Calasanz M. D. C. Nouioui I. Rohde M. Spröer C. (2016). *Geodermatophilus pulveris* sp. nov., a gamma-radiation-resistant actinobacterium isolated from the Sahara desert. *Int. J. Syst. Evol. Microbiol.* 66 3828–3834. 10.1099/ijsem.0.001272 27381197

[B27] Hindley J. (1967). Fractionation of 32P-labelled ribonucleic acids on polyacrylamide gels and their characterization by fingerprinting. *J. Mol. Biol.* 30 125–136. 10.1016/0022-2836(67)90248-3 4865141

[B28] Hnilicova J. Matejckova J. J. Sikova M. Pospisil J. Halada P. Panek J. (2014). Ms1, a novel sRNA interacting with the RNA polymerase core in mycobacteria. *Nucleic Acids Res.* 42 11763–11776. 10.1093/nar/gku793 25217589 PMC4191392

[B29] Hoch P. G. Burenina O. Y. Weber M. H. Elkina D. A. Nesterchuk M. V. Sergiev P. V. (2015). Phenotypic characterization and complementation analysis of *Bacillus subtilis* 6S RNA single and double deletion mutants. *Biochimie* 117 87–99. 10.1016/j.biochi.2014.12.019 25576829

[B30] Hoch P. G. Schlereth J. Lechner M. Hartmann R. K. (2016). *Bacillus subtilis* 6S-2 RNA serves as a template for short transcripts in vivo. *RNA* 22 614–622. 10.1261/rna.055616.115 26873600 PMC4793215

[B31] Hopwood D. A. (2006). Soil to genomics: the *Streptomyces* chromosome. *Annu. Rev. Genet.* 40 1–23. 10.1146/annurev.genet.40.110405.090639 16761950

[B32] Jeong Y. Kim J. N. Kim M. W. Bucca G. Cho S. Yoon Y. J. (2016). The dynamic transcriptional and translational landscape of the model antibiotic producer *Streptomyces coelicolor* A3(2). *Nat. Commun.* 7:11605. 10.1038/ncomms11605 27251447 PMC4895711

[B33] Jones A. J. Venkataramanan K. P. Papoutsakis T. (2016). Overexpression of two stress-responsive, small, non-coding RNAs, 6S and tmRNA, imparts butanol tolerance in *Clostridium acetobutylicum*. *FEMS Microbiol. Lett.* 363:fnw063. 10.1093/femsle/fnw063 26989157

[B34] Kalvari I. Argasinska J. Quinones-Olvera N. Nawrocki E. P. Rivas E. Eddy S. R. (2017). Rfam 13.0: shifting to a genome-centric resource for non-coding RNA families. *Nucleic Acids Res.* 46 D335–D342. 10.1093/nar/gkx1038 29112718 PMC5753348

[B35] Kim W. Hwang S. Lee N. Lee Y. Cho S. Palsson B. (2020). Transcriptome and translatome profiles of *Streptomyces* species in different growth phases. *Sci. Data* 7:138. 10.1038/s41597-020-0476-9 32385251 PMC7210306

[B36] Klocko A. D. Wassarman K. M. (2009). 6S RNA binding to Esigma(70) requires a positively charged surface of sigma(70) region 4.2. *Mol. Microbiol.* 73 152–164. 10.1111/j.1365-2958.2009.06758.x 19538447 PMC2758106

[B37] Köhler K. Duchardt-Ferner E. Lechner M. Damm K. Hoch P. G. Salas M. (2015). Structural and mechanistic characterization of 6S RNA from the hyperthermophilic bacterium *Aquifex aeolicus*. *Biochimie* 117 72–86. 10.1016/j.biochi.2015.03.004 25771336

[B38] Korf I. Yandell M. Bedell J. (2003). *BLAST.* Sebastopol, CA: O’Reilly & Associates.

[B39] Labeda D. P. Kroppenstedt R. M. (2004). Emended description of the genus *Glycomyces* and description of *Glycomyces algeriensis* sp. nov., *Glycomyces arizonensis* sp. nov. and *Glycomyces lechevalierae* sp. nov. *Int. J. Syst. Evol. Microbiol.* 54(Pt 6), 2343–2346. 10.1099/ijs.0.63089-0 15545481

[B40] Lee Y. Lee N. Hwang S. Kim W. Jeong Y. Cho S. (2020). Genome-scale determination of 5′ and 3′ boundaries of RNA transcripts in *Streptomyces* genomes. *Sci. Data* 7:436. 10.1038/s41597-020-00775-w 33319794 PMC7738537

[B41] Lorenz R. Bernhart S. H. Honer Zu Siederdissen C. Tafer H. Flamm C. Stadler P. F. (2011). ViennaRNA Package 2.0. *Algorithms Mol. Biol.* 6:26. 10.1186/1748-7188-6-26 22115189 PMC3319429

[B42] Markham N. R. Zuker M. (2008). UNAFold: software for nucleic acid folding and hybridization. *Methods Mol. Biol.* 453 3–31. 10.1007/978-1-60327-429-6_1 18712296

[B43] Martini M. C. Zhou Y. Sun H. Shell S. S. (2019). Defining the transcriptional and post-transcriptional landscapes of. *Front. Microbiol.* 10:591. 10.3389/fmicb.2019.00591 30984135 PMC6448022

[B44] Mikulík K. Bobek J. Zídková J. Felsberg J. (2014). 6S RNA modulates growth and antibiotic production in *Streptomyces coelicolor*. *Appl. Microbiol. Biotechnol.* 98 7185–7197. 10.1007/s00253-014-5806-4 24859522

[B45] Moody M. J. Young R. A. Jones S. E. Elliot M. A. (2013). Comparative analysis of non-coding RNAs in the antibiotic-producing *Streptomyces* bacteria. *BMC Genomics* 14:558. 10.1186/1471-2164-14-558 23947565 PMC3765725

[B46] Neusser T. Polen T. Geissen R. Wagner R. (2010). Depletion of the non-coding regulatory 6S RNA in *E. coli* causes a surprising reduction in the expression of the translation machinery. *BMC Genomics* 11:165. 10.1186/1471-2164-11-165 20222947 PMC2848244

[B47] Nikitina E. Liu S. W. Li F. N. Buyantueva L. Abidueva E. Sun C. H. (2020). sp. nov., an actinobacterium isolated from steppe soil. *Int. J. Syst. Evol. Microbiol.* 70 1356–1363. 10.1099/ijsem.0.003923 31808739

[B48] Nouioui I. Carro L. García-López M. Meier-Kolthoff J. P. Woyke T. Kyrpides N. C. (2018). Genome-based taxonomic classification of the phylum. *Front. Microbiol.* 9:2007. 10.3389/fmicb.2018.02007 30186281 PMC6113628

[B49] Panchapakesan S. S. Unrau P. J. (2012). E. coli 6S RNA release from RNA polymerase requires σ70 ejection by scrunching and is orchestrated by a conserved RNA hairpin. *RNA* 18 2251–2259. 10.1261/rna.034785.112 23118417 PMC3504675

[B50] Panek J. Bobek J. Mikulik K. Basler M. Vohradsky J. (2008). Biocomputational prediction of small non-coding RNAs in *Streptomyces*. *BMC Genomics* 9:217. 10.1186/1471-2164-9-217 18477385 PMC2422843

[B51] Pánek J. Krásny L. Bobek J. Jezková E. Korelusová J. Vohradsky J. (2011). The suboptimal structures find the optimal RNAs: homology search for bacterial non-coding RNAs using suboptimal RNA structures. *Nucleic Acids Res.* 39 3418–3426. 10.1093/nar/gkq1186 21193488 PMC3082871

[B52] Perez J. T. Varble A. Sachidanandam R. Zlatev I. Manoharan M. García-Sastre A. (2010). tenOever: Influenza a virus-generated small RNAs regulate the switch from transcription to replication. *Proc. Natl. Acad. Sci. U. S. A.* 107, 11525–11530. 10.1073/pnas.1001984107 20534471 PMC2895093

[B53] Perez J. T. Zlatev I. Aggarwal S. Subramanian S. Sachidanandam R. Kim B. (2012). tenOever: A small-RNA enhancer of viral polymerase activity. *J. Virol.* 86, 13475-13485. 10.1128/JVI.02295-12 23035211 PMC3503082

[B54] Rediger A. Geissen R. Steuten B. Heilmann B. Wagner R. Axmann I. M. (2012). 6S RNA - an old issue became blue-green. *Microbiology* 158(Pt 10), 2480–2491. 10.1099/mic.0.058958-0 22767549

[B55] Romero D. A. Hasan A. H. Lin Y. F. Kime L. Ruiz-Larrabeiti O. Urem M. (2014). A comparison of key aspects of gene regulation in *Streptomyces coelicolor* and *Escherichia coli* using nucleotide-resolution transcription maps produced in parallel by global and differential RNA sequencing. *Mol. Microbiol.* 94 963–987. 10.1111/mmi.12810 25266672 PMC4681348

[B56] Schoch C. L. Ciufo S. Domrachev M. Hotton C. L. Kannan S. Khovanskaya R. (2020). NCBI Taxonomy: a comprehensive update on curation, resources and tools. *Database* 2020:baaa062. 10.1093/database/baaa062 32761142 PMC7408187

[B57] Sedlyarova N. Rescheneder P. Magán A. Popitsch N. Rziha N. Bilusic I. (2017). Natural RNA polymerase aptamers regulate transcription in *E. coli*. *Mol. Cell* 67 30–43.e6. 10.1016/j.molcel.2017.05.025 28648779 PMC5535762

[B58] Sharma C. M. Hoffmann S. Darfeuille F. Reignier J. Findeiss S. Sittka A. (2010). The primary transcriptome of the major human pathogen *Helicobacter pylori*. *Nature* 464 250–255. 10.1038/nature08756 20164839

[B59] Shephard L. Dobson N. Unrau P. J. (2010). Binding and release of the 6S transcriptional control RNA. *RNA* 16 885–892. 10.1261/rna.2036210 20354151 PMC2856883

[B60] Sikova M. Janouskova M. Ramaniuk O. Palenikova P. Pospisil J. Bartl P. (2019). Ms1 RNA increases the amount of RNA polymerase in *Mycobacterium smegmatis*. *Mol. Microbiol.* 111 354–372. 10.1111/mmi.14159 30427073

[B61] Šmídová K. Ziková A. Pospíšil J. Schwarz M. Bobek J. Vohradsky J. (2019). DNA mapping and kinetic modeling of the HrdB regulon in *Streptomyces coelicolor*. *Nucleic Acids Res.* 47 621–633. 10.1093/nar/gky1018 30371884 PMC6344877

[B62] Sridhar J. Gunasekaran P. (2013). Computational small RNA prediction in bacteria. *Bioinform. Biol. Insights* 7 83–95. 10.4137/BBI.S11213 23516022 PMC3596055

[B63] Sridhar J. Rafi Z. A. (2007). Small RNA identification in *Enterobacteriaceae* using synteny and genomic backbone retention. *OMICS* 11 74–99. 10.1089/omi.2006.0006 17411397

[B64] Steuten B. Setny P. Zacharias M. Wagner R. (2013). Mapping the spatial neighborhood of the regulatory 6S RNA bound to *Escherichia coli* RNA polymerase holoenzyme. *J. Mol. Biol.* 425 3649–3661. 10.1016/j.jmb.2013.07.008 23867276

[B65] Strnad H. Patek M. Fousek J. Szokol J. Ulbrich P. Nesvera J. (2014). Genome sequence of *Rhodococcus erythropolis* strain CCM2595, a phenol derivative-degrading bacterium. *Genome Announc.* 2:e00208-14. 10.1128/genomeA.00208-14 24652983 PMC3961730

[B66] Svensson S. L. Sharma C. M. (2016). Small RNAs in bacterial virulence and communication. *Microbiol. Spectr.* 4 169–212. 10.1128/microbiolspec.VMBF-0028-2015 27337442

[B67] Trotochaud A. E. Wassarman K. M. (2004). 6S RNA function enhances long-term cell survival. *J. Bacteriol.* 186 4978–4985. 10.1128/JB.186.15.4978-4985.2004 15262935 PMC451630

[B68] Trotochaud A. E. Wassarman K. M. (2005). A highly conserved 6S RNA structure is required for regulation of transcription. *Nat. Struct. Mol. Biol.* 12 313–319. 10.1038/nsmb917 15793584

[B69] Trotochaud A. E. Wassarman K. M. (2006). 6S RNA regulation of pspF transcription leads to altered cell survival at high pH. *J. Bacteriol.* 188 3936–3943. 10.1128/JB.00079-06 16707685 PMC1482906

[B70] Vockenhuber M. P. Sharma C. M. Statt M. G. Schmidt D. Xu Z. Dietrich S. (2011). Deep sequencing-based identification of small non-coding RNAs in *Streptomyces coelicolor*. *RNA Biol.* 8 468–477. 10.4161/rna.8.3.14421 21521948 PMC3218513

[B71] Vogel D. W. Hartmann R. K. Struck J. C. Ulbrich N. Erdmann V. A. (1987). The sequence of the 6S RNA gene of *Pseudomonas aeruginosa*. *Nucleic Acids Res.* 15 4583–4591. 10.1093/nar/15.11.4583 2438656 PMC340881

[B72] Wassarman K. M. (2018). 6S RNA, a global regulator of transcription. *Microbiol. Spectr.* 6: RWR-0019-2018 10.1128/microbiolspec.RWR-0019-2018 29916345 PMC6013841

[B73] Wassarman K. M. Saecker R. M. (2006). Synthesis-mediated release of a small RNA inhibitor of RNA polymerase. *Science* 314 1601–1603. 10.1126/science.1134830 17158328

[B74] Wassarman K. M. Storz G. (2000). 6S RNA regulates *E. coli* RNA polymerase activity. *Cell* 101 613–623. 10.1016/s0092-8674(00)80873-9 10892648

[B75] Wehner S. Damm K. Hartmann R. K. Marz M. (2014). Dissemination of 6S RNA among bacteria. *RNA Biol.* 11 1467–1478. 10.4161/rna.29894 25483037 PMC4615782

[B76] Wurm R. Neusser T. Wagner R. (2010). 6S RNA-dependent inhibition of RNA polymerase is released by RNA-dependent synthesis of small de novo products. *Biol. Chem.* 391 187–196. 10.1515/BC.2010.018 20030589

[B77] Xing K. Qin S. Zhang W. D. Cao C. L. Ruan J. S. Huang Y. (2014). *Glycomyces phytohabitans* sp. nov., a novel endophytic actinomycete isolated from the coastal halophyte in Jiangsu, East China. *J. Antibiot.* 67 559–563. 10.1038/ja.2014.40 24736858

