# Supplementary material for: Ms1 RNA Interacts With the RNA Polymerase Core in Streptomyces coelicolor and Was Identified in Majority of Actinobacteria Using a Linguistic Gene Synteny Search
Source: Front Microbiol. 2022 May 11;13:848536. doi: 10.3389/fmicb.2022.848536 (PMC9130861; doi:10.3389/fmicb.2022.848536)
Supplement: Supplementary file 1 [file Data_Sheet_1.docx]

**Sequences of 12 predicted sRNAs (*Computational homology search for new sRNAs)***

Positions of oligonucleotides used for detection of predicted sRNA sequences by Northern blotting (Figures 1 and 2) are underlined

Str_1

StrcoelicolorA3_2__2_1915678_1915941_2e_30_SambATCC23877_GCA_001267885_1915854_1916153

GGGACGGTCACCCCGGTCGTCGCGACGGTACGAGGGACGGTCGCCCCGGTCCCCACGCTCACCACGGTCGTCGCGGCGGAAGGACGGACGGTCACCACCCCGGTCATCGCGACGGAAGCCCCGGTCGCCGCCACGGTCCCGGTCGTCCCGACGCCCGTAGCCGGCACCGCGGTTGTCCTCGCGACGACCGTCGCGGCGGTCATCACGGCCGTCATCCCGACGGTCGTCACGACGGAAGCCGCCGCGCTGCCCACCGCGGTCATCGCGCCGATCGTCACGACGGTCATCACGGCGATC

Str_2 StrcoelicolorA3_2__6_3275271_3274972_8e_80_Ssca87_GCA_000091305_6167357_6167656

CCGAGGCGCACCGGGCGGGAGAACTGGGGACCGGGCCGCTCGTCGCCTACGCCGTCACACCGGCGCTGCGCGAGTGGTACCTGTCGGACGACATCGAGGAGCTGGAGTACGCCGCCCTCAACCGGGCCGCCCTGGCCTCGCTGCGCCTGCTGGCGGCCGACCCGGCCGGGGTGCGGCGCCGGGTCGTGGTCGCCGCGGACGTCCCCGACGGCGCCGCCGTGGCCGACCCGGACCGGGGACTCGATCCGGCGGCGCTGGGCGAGGTCCGCCTCGCCGGACCGCTGCCGCTGGCCAAGGCGG

Str_3 StrcoelicolorA3_2__14_1881845_1881948_3e_09_SgrigriseTsNBRC_GCA_000010605_1691770_1692069

GGTGCGTGCGATCGCAAGGCGCCGGAGCGCCCTCGTGGCGGAGCCACGCGGGCGCTTCGGCAACGCGGCGAGCGTGCGTGCCAGGCGTCGCCGGGCAGGCGGGAATCATCAGACAGGCCCTAGGTTACCGGGCCGGGCAGACCCCTCCGACAGCCGTCCGCAGGCCCCGGTCACTGCCGGATCGCCCAGCCCCGTTCCTTCAGGGCCGCCGTGAGGACGGCGGCGGCCTTCGGCTCCACCATGAGCTGCACCAGACCCGCCTGCTGCCCGGTCGCGTGCTCGATCCGTACGTCCTCCAC

Str_4 StrcoelicolorA3_2__11_1553780_1553709_4e_07_SplT_GCA_000802245_939010_939309_extended

GCGACCGTTGGGGCGGCGTCTCATGGCGGTCGTCCCCCCTGGACCGAAGCCGGTGAACCGAACGGTCACCACGGTCCCGGCGCCGCGCCCGCACCCCATCGCGCCCGGGACGGGAGCCGCCTGCGCCGCGCGGCGGGGAGGGCCGCCCCGGTCTGATCCCCGAGGACGAGTACCGCACCCGCCGGAGGGTGCGCAACGAATCGCCGTGGGGTGCCCCGGGCACGCAACGATCCGCTCACCGGCGCGCAACAGGTCCGTCTTGTCCGGCCGAGTCCGCCGACCGTACCGTCTATCTGGCCCCCTGAACCCCCCTCGTGACCGGTGAGGAACACGC

Str_5 Sco_AL939123_1_191892_192006_Snod_GCA_000819545_3000867_3001166_reconstrTcted_from_Save_BLAST

TGACGCGGGGGCTGAGCGGTGCGGGGCGGTCTTCCGGCGCGGGGTTTCTTCGCCCCCGCCGCCCCTTCCCTTCCCGACCCTGGGGGCGCCACCCCCAGACCCCCGCTTTCGGCCTGAACGGCCTCGTCCTCAAACGCCGGACGGGCTGGGGGCTCAACGGCCTCGTCCTCAAGTGCCGGACGGGCTGGGGGGGCTCAACGGTCTCGTCCTCGAGTGCCGGACGGGCTAAAGTGACCCGCGTGTTCCTCCTCTTGGCCTAGGACCCGCCCCGCACGCGACCGCCGCATCCGGCCGTCGCGC

Str_6

ScoeA3_GCA_000203835_2249619_2249798_-

GGACCGGCCCACCCCGAATCACGGACACCGCGCCGAAAAAGTTCCGCGCGACACCACTTCCCGCATTCTTGCCGCCGTTACCGATTCGTGTCACTCGCCTGTGGACAACTGGCCGCGATATGTCGGCGCTTACGGCCTTTGCGGAGCTTGCACGGCGGGATCGTCGGGGCGGGCCGCCGG

Str_7

StrcoelicolorA3_2__4_4186663_4186807_3e_31_SambATCC23877_GCA_001267885_4103311_4103490

GGCCTACGATCGTCACACGAAAGCACTGCCGGGGTGCCGCACAGCAGCGTAACTTAGCGTTCCAAGACACCCGCCGACTGCCGCCACCGCCACTGGCACCGCCGCCACCAACCGCCCGTGTACCGACCGCCGAGGGGTCCCGTTC

Str_8

ScoeA3_GCA_000203835_3946569_3946748_-

CCGTGGATGGCGCAGGCGTACGGAGACCCAGCGGGCCCTGCGGCGCAGGATGCGCGGAATACCCACCCGAAGGTCCGCACCCGCGAGTTGCGGGGTGCCGCCGCGTCGGTGGGTGCTGCTCACGCCGCTCGTGGAGCGGCCGTCGCGTGCTACGTCACTGTAGTCGTGCGTCCAGCCCAT

Str_9

ScoeA3_GCA_000203835_1671946_1672125_+

GCTTCGGGGCGCGAGACTCAGGTGGTGCGGGGGCCCTCTAGAAGGCGCACATTCGGCGGCACATACAACGAGCACCGGGCGTCGTGGTCGCCTCGGTCGCAGGGGTGCGGTCGCTCGTGGTGCTCATGCCGATCAGTAAAGCAGACGTATGCCTCTGGCCGGAGACCGCTGTCCGGATAC

Str_10

StrcoelicolorA3_2__1_5592190_5592364_1e_67_SambATCC23877_GCA_001267885_5219563_5219742

CTCATGAGGTGTGACCTGCGTCAAAGAAAACCGGTCCGTATCCATTCGGTGGAAGTGCGTGAGGGCGCGCAGTATCGCAGTCACATCGCCGAATTGAGTGGTTTTGCTCGCGCGCGTTGCCCAAGCGATGCCGACCGATGTTGGTCATCGGGTCCTGGGATGCGGGATAATGGCTGGGAA

Str_11

StrcoelicolorA3_2__2_1609809_1609633_2e_64_Ssca87_GCA_000091305_8296565_8296744

TTCCAGGAGCGTCGCGCACCGTCGATGGTGTCGCGGGCAGCCCACCACCACCCGGCTTCCGGGAGCGGTCGGTGGGCCACTCGCGTGCTGCACGTATGTGCCCAGACCAGGGGAGCGGCTGCCCGGCAGGTCCCGAGCGGACCCGGCGGGTTTCCCCGGCTGCGGATTGCGACCTCCCT

Str_12

ScoeA3_2__5808519_5808699_+_ML5_GCA_000177655_6788539_6788718_+

CGCGTCTGCTCGGCTCTGCCTGGGTCGATGGCGGACGGGATGGGAGCTCCCCGCGCATGCCACTACGGACCCGCAGTCGGAAGCGGCGCTCTTTCCATCCCGGTGCCGGCCGATCCCCCGCCGGAGGCATCATCTTGTGCCTGCGGATGACGTCCCGTCCTTGCCCGCATGCACGCAGCG

**Sequence of Str_13/src3559/Ms1** ***(final sequence verified by 5´RACE and 3´RACE)***

AACGGCCCGCGAGACCAAGGACATCCGAAAGGATCACCTTAAACACGCATATGGCCCCACGGACCGAGCATGGACACCGGGCACCCACGCGACGTCGACCCGTCGATTACGGGCCAGCCGCACCAGGTCACGGGCGAAGTTCCCGGCCTGATGGGCACATATCGAGGACGCTTGGTAACCGGGTGGTCATGCCAGCGGCGGTACGAGCATTCGTACCGCCGCATTCC

Sequences of biotinylated oligos used for Northern blotting (Figures 1 and 2):

2786 Str1_F btn-GACGGAAGCCCCGGTCGCCGCCACG

2787 Str1_R btn-CGTGGCGGCGACCGGGGCTTCCGTC

2788 Str_2_F btn-GAGTGGTACCTGTCGGACGACATCG

2789 Str_2_R btn-CGATGTCGTCCGACAGGTACCACTC

2790 Str_3_F btn-CGTTCCTTCAGGGCCGCCGTGAGGA

2791 Str_3_R btn-TCCTCACGGCGGCCCTGAAGGAACG

2792 Str_4_F btn-GCACGCAACGATCCGCTCACCGGCG

2793 Str_4_R btn-CGCCGGTGAGCGGATCGTTGCGTGC

2794 Str_5_F btn-CTGAACGGCCTCGTCCTCAAACGCC

2795 Str_5_R btn-GGCGTTTGAGGACGAGGCCGTTCAG

2828 Str6_F btn-CCGATTCGTGTCACTCGCCTGTGGA

2829 Str6_R btn-TCCACAGGCGAGTGACACGAATCGG

2830 Str11_F btn-ACGTATGTGCCCAGACCAGGGGAGC

2831 Str11_R btn-GCTCCCCTGGTCTGGGCACATACGT

2832 Str8_F btn-GAATACCCACCCGAAGGTCCGCACC

2833 Str8_R btn-GGTGCGGACCTTCGGGTGGGTATTC

2834 Str9_F btn-GTCGCTCGTGGTGCTCATGCCGATC

2835 Str9_R btn-GATCGGCATGAGCACCACGAGCGAC

2836 Str10_F btn-CATCGCCGAATTGAGTGGTTTTGCT

2837 Str10_R btn-AGCAAAACCACTCAATTCGGCGATG

2838 Str7_F btn-GTAACTTAGCGTTCCAAGACACCCG

2839 Str7_R btn-CGGGTGTCTTGGAACGCTAAGTTAC

2840 Str12_F btn-CACTACGGACCCGCAGTCGGAAGCG

2841 Str12_R btn-CGCTTCCGACTGCGGGTCCGTAGTG

2842 Str_5S btn-CGCTGTAAGGCTTAGCTTCCGGGTT

2796 Str_13 (ssrS/scr3559/Ms1) btn-TCCTTGGTCTCGCGGGCCGTTGAGT

3813 Sc_probe A btn-GGTCCCGAGTAAGCGGAAGTGCTCG

3812 Sc_probe B btn-GTCCGTGGGGCCATATGCGTGTTA

3811 Sc_probe C btn- GCTCGTACCGCCGCTGGCATGACCA

Primers used for RT-qPCR validation of Str11/Str13 association with RNA polymerase/HrdB (Figure 3)

3155/Sco_Str11_F1 CGTCGCGCACCGTCGATGGT

3156/Sco_Str11_R1 TACGTGCAGCACGCGAGTGG

3225/Sco_Str13_F GATCACCTTAAACACGCATATGG

3226/Sco_Str13_R GAACTTCGCCCGTGACCT

3298/Sco3352_F CTTGGAGATCGAGGACCTGG

3299/Sco3352_R CCAGCTCCACCACGTACTC

3300/Sco3710 _F TTCTGCTACGTCCACCCCAT

3301/Sco3710 _R CAGGACGAGGAAACCCAGG

3302/Sco2013_F ctggaggaggaggggtact

3303/Sco2013_R catcttgggcatcttcacgt

1617/Sco_16SrRNA_F TGTCACCCGATTACGGGTAT

1618/Sco_16SrRNA_R ACCGAAGTGGTTCATCGTTC

1024/Msmeg_Ms1_F GCCGGAAGAGAAGGCTAGAT

1025/Msmeg_Ms1_R CGTCCGCTTTTCGAAACTAC

2816/Msmeg_rpoB_F CACCGAACCGAGTTTCATTT

2817/Msmeg_rpoB_R TCGAAGGAATCCGTCTGAAC

989/Msmeg_rpoC_F CGACGAGATCTGGAACACCT

990/Msmeg_rpoC_R GGTGAAGTACTCGCCGTAGC

**Supplementary Table S1**. Characteristics of presented expressed *Streptomyces* sRNAs.

| RNA | Predicted function | Gen. locus in S. *coelicolor* A3(2) (GenBank ID AL645882.2) # | Conserved in * | Approx. length (nts) | Strand | Genomic position | Genomic locus † | Predicted sequence in *S. coelicolor* (oligo for expression verification in lowercase) ** |
| --- | --- | --- | --- | --- | --- | --- | --- | --- |
| Str1 | cis-acting asRNA | - (4 hits at different gen. loci in S. co., also see the 8th column) | Actinobacteria | 90, 120 | + (RNA), - (fragments) | + strand opposite to 'tetratricopeptide repeat protein' that is on the - strand | 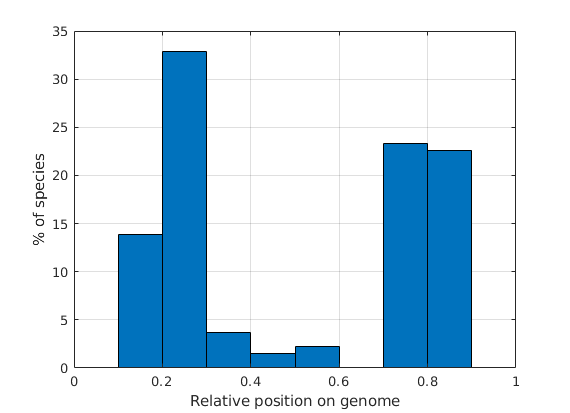 | - (impossible to predict 5' and 3' ends for overlapping incomplete BLAST hits in single species) |
| Str3 | cis-acting asRNA | 1882014 : 1882198 | Actinobacteria | 160 | + (RNA), - (fragments) | overlaps both the prephenate dehydrogenase that is on - strand and IGR between 'prephenate dehydrogenase' and '[cytidylate OR (d)CMP] kinase' | 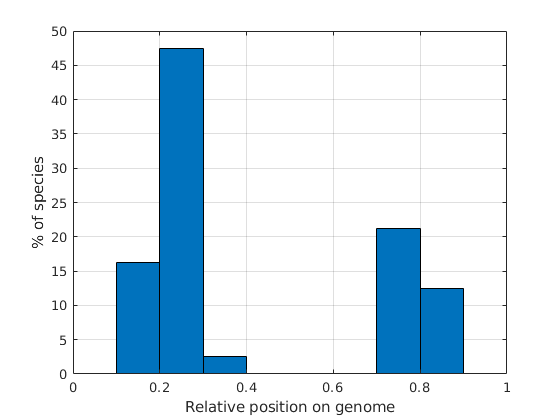 | GUCACUGCCGGAUCGCCCAGCCCcguuccuucagggccgccgugaggacGGCGGCGGCCUUCGGCUCCACCAUGAGCUGCACCAGACCCGCCUGCUGCCCGGUCGCGUGCUCGAUCCGUACGUCCUCCACGUUGACCCCGGCUCGUCCCGCGUCCGCGAAGAUGCGGGCCAGCUGACCGGGCUG |
| Str5 | small noncoding RNA | 5872966 : 5873065 (+ another 8 gapped, but very good BLAST hits at different gen. loci in S. co.) | Streptomyces | 130 | + | IGR between 'methylmalonyl Co-A mutase-associated GTPase MeaB' - 'MFS transporter' (+ a lot other IGRs with different flanking genes) | 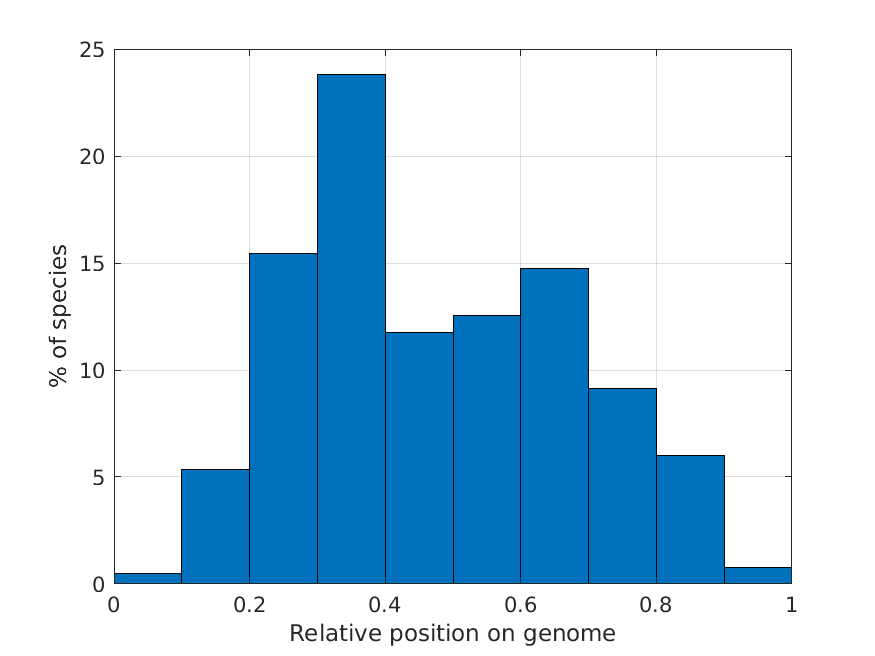 | CUUCGCCCCCGCCGCCCCUUCCCUUCCCGACCCUGGGGGCGCCACCCCCAGACCCCCGCUUUCGGCcugaacggccucguccucaaacgccGGACGGGCU |
| Str8 | small noncoding RNA | 3946749 : 3946619 | Streptomyces | 130 | - | IGR between ['colicin V synthesis protein' OR 'serine protease'] - 'alpha/beta hydrolase' | 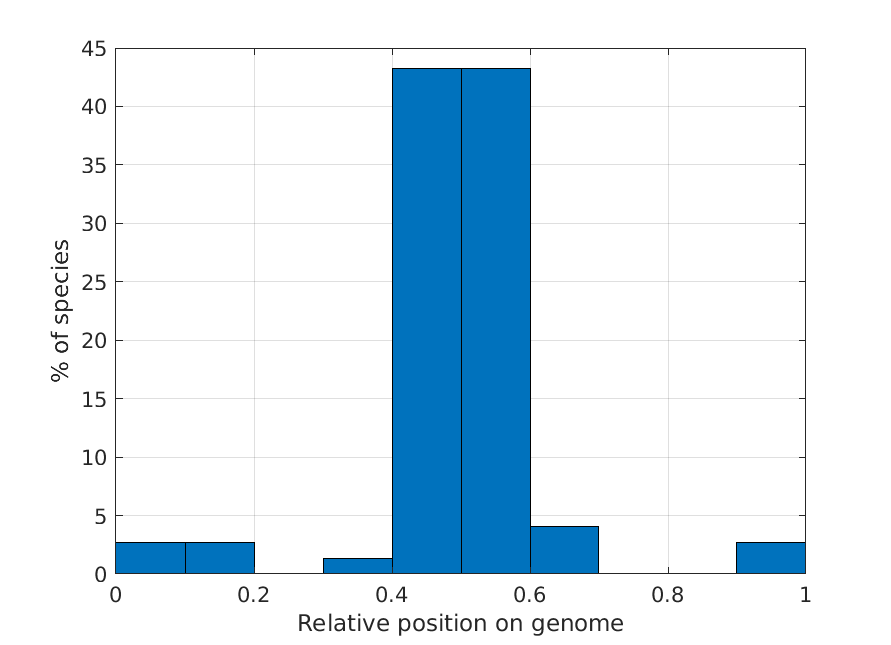 | CCGUGGAUGGCGCAGGCGUACGGAGACCCAGCGGGCCCUGCGGCGCAGGAUGCGCGgaauacccacccgaagguccgcaccCGCGAGUUGCGGGGUGCCGCCGCGUCGGUGGGUGCUGCUCACGCCGCUC |
| Str10 | small noncoding RNA | 5592268 : 5592388 | Streptomyces | 120 | + | IGR between ['DUF3117 domain-containing protein' OR 'methyltransferase'] - ['enoyl-CoA hydratase' OR 'acyl CoA isomerase'] | 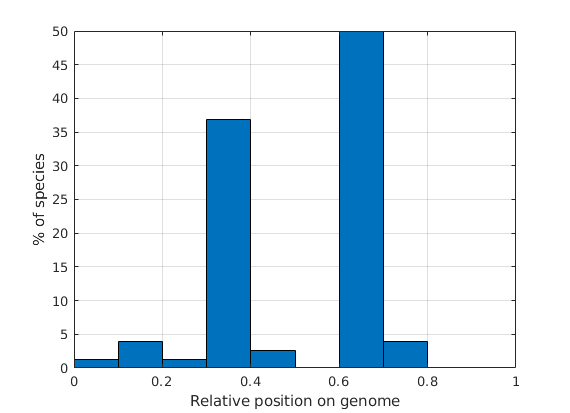 | caucgccgaauugagugguuuugcucGCGCGCGUUGCCCAAGCGAUGCCGACCGAUGUUGGUCAUCGGGUCCUGGGAUGCGGGAUAAUGGCUGGGAAGCAAUGUGUUCGAUGCCGGUGUCG |
| Str11 | small noncoding RNA | 1609864 : 1609645 | Streptomyces | 220 | - | IGR between '30S ribosomal protein S4' - ['AAA family ATPase' OR 'Recombination factor protein RarA' OR 'replication-associated recombination protein A'] (they are synonyms) | 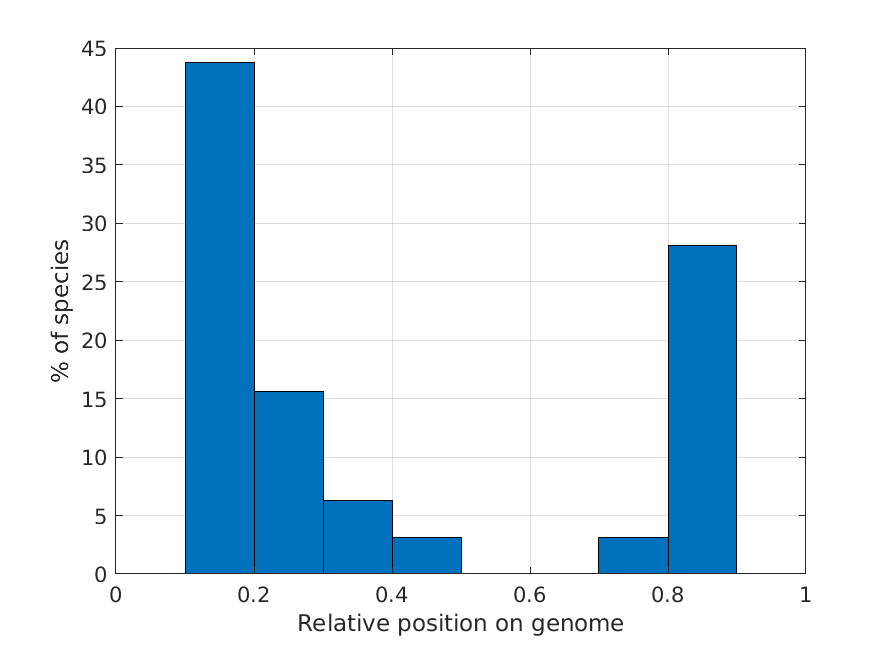 | UUCCAGGAGCGUCGCGCACCGUCGAUGGUGUCGCGGGCAGCCCACCACCACCCGGCUUCCGGGAGCGGUCGGUGGGCCACUCGCGUGCUGCacguaugugcccagaccaggggagcGGCUGCCCGGCAGGUCCCGAGCGGACCCGGCGGGUUUCCCCGGCUGCGGAUUGCGACCUCCCU |
| Str13 | small noncoding RNA | 3934693 : 3934920 | Actinobacteria | 230 | - | IGR between ['IB HAD hydrolase' OR 'inhibition morphological differetiation'] - ['oxidoreductase' OR 'Fic' OR 'hexosaminidase'] | 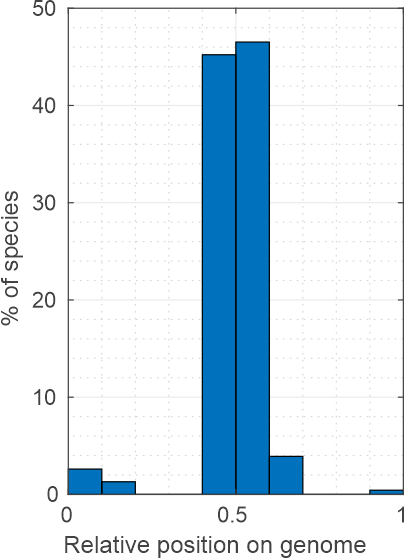 | AACGGCCCGCGAGACCAAGGACAUCCGAAAGGAUCACCUUAAACACGCAUAUGGCCCCACGGACCGAGCAUGGACACCGGGCACCCACGCGACGUCGACCCGUCGAUUACGGGCCAGCCGCACCAGGUCACGGGCGAAGUUCCCGGCCUGAUGGGCACAUAUCGAGGACGCUUGGUAACCGGGUGGUCAUGCCAGCGGCGGUACGAGCAUUCGUACCGCCGCAUUCCU |

- Not applicable (from reasons shown in parenthesis).

* Based on sequence similarity by NCBI BLASTn (with parameters Word size = 7, Match/Mismatch Scores = 1, -1, Gap Costs = Existence: 2 Extension: 1), synteny and genomic loci similarity.

** See Materials and Methods for details on prediction.

† Distribution in first 100 homologs found by BLAST in nt database.

# Computationally predicted based on sequence similarity, except for Str7 5' end identified by 5' RACE.

**
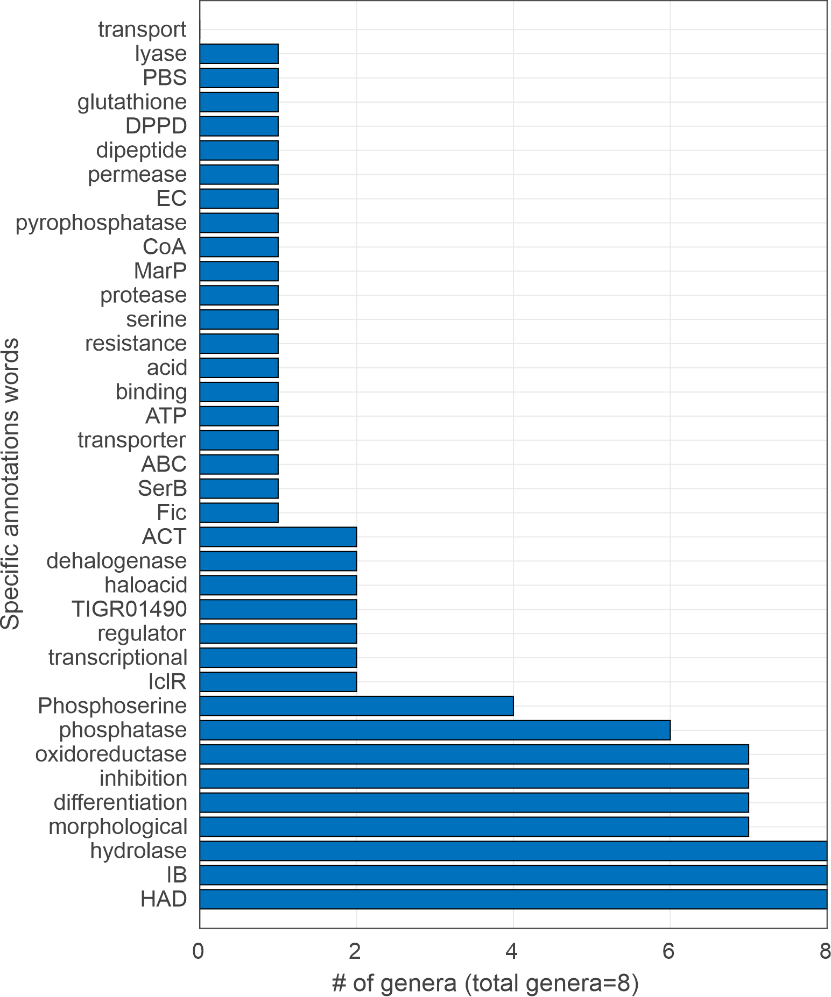
Supplementary Figure S1**. Specific words and their occurrence in synteny annotations of flanking genes of homologs of *M. smegmatis* Ms1 RNA across eight genera – *Mycobacterium*, *Mycolicibacterium*, *Rhodococcus, Nocardia*, *Gordonia*, *Mycobacteroides*, *Hoyosella* and *Tsukamurella.* The homologs had sequences similar to *M. smegmatis* Ms1 RNA identified by BLAST (with BLAST E-value < 10^-20^) and conserved Ms1 synteny.

**
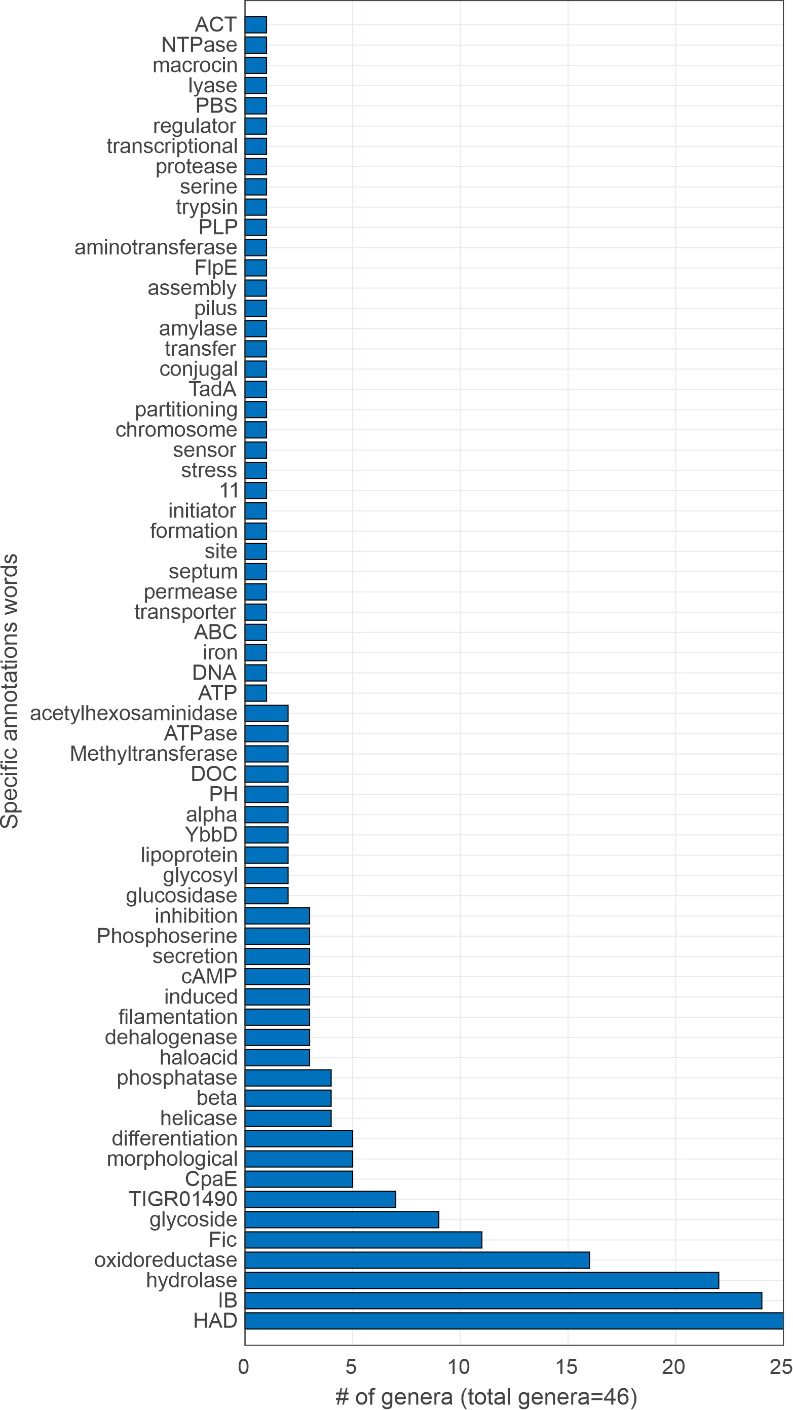
Supplementary Figure S2**. Specific words and their occurrence in synteny annotations of Ms1 IGRs homologous to the 1^st^ synteny hit in *Streptomyces*. Specific words with higher than average occurrence (3.4 genera), 10 words starting 'HAD' up to 'phosphatase' (see y-axis), were used for the update of the original phrases in Table 1. The updated phrases are shown in Table 2. 'TIGR01490' was not used as it was a part of 'IB HAD hydrolase' annotation. Some of the most occurring specific words semantically bound with other specific words that were not most occurring to form a phrase. Here 'morphological' and 'differentiation' bound with 'inhibition'. Specific word 'beta' occurred in 'beta-N-acetylhexosaminidase' and 'beta-glucosidase-like glycosyl hydrolase' resulting into two new synteny phrases 'beta acetylhexosaminidase ' and 'beta glycosyl glucosidase'. 'glycoside' bound with 'hydrolase' producing 'glycoside hydrolase' phrase and 'phosphatase' bound with 'phosphoserine' producing 'phosphoserine phosphatase' phrase. Phrases 'Fic' and 'CpaE' originated from 'filamentation induced by cAMP protein Fic' and 'helicase/secretion neighborhood CpaE-like protein' annotations, respectively. A CpaE-like protein represented an extended Ms1 RNA synteny and as it is a next Ms1 IGR flanking gene after HAD hydrolase. It occurred here as the HAD hydrolase gene annotation was missing in genomic annotations of some species.

**
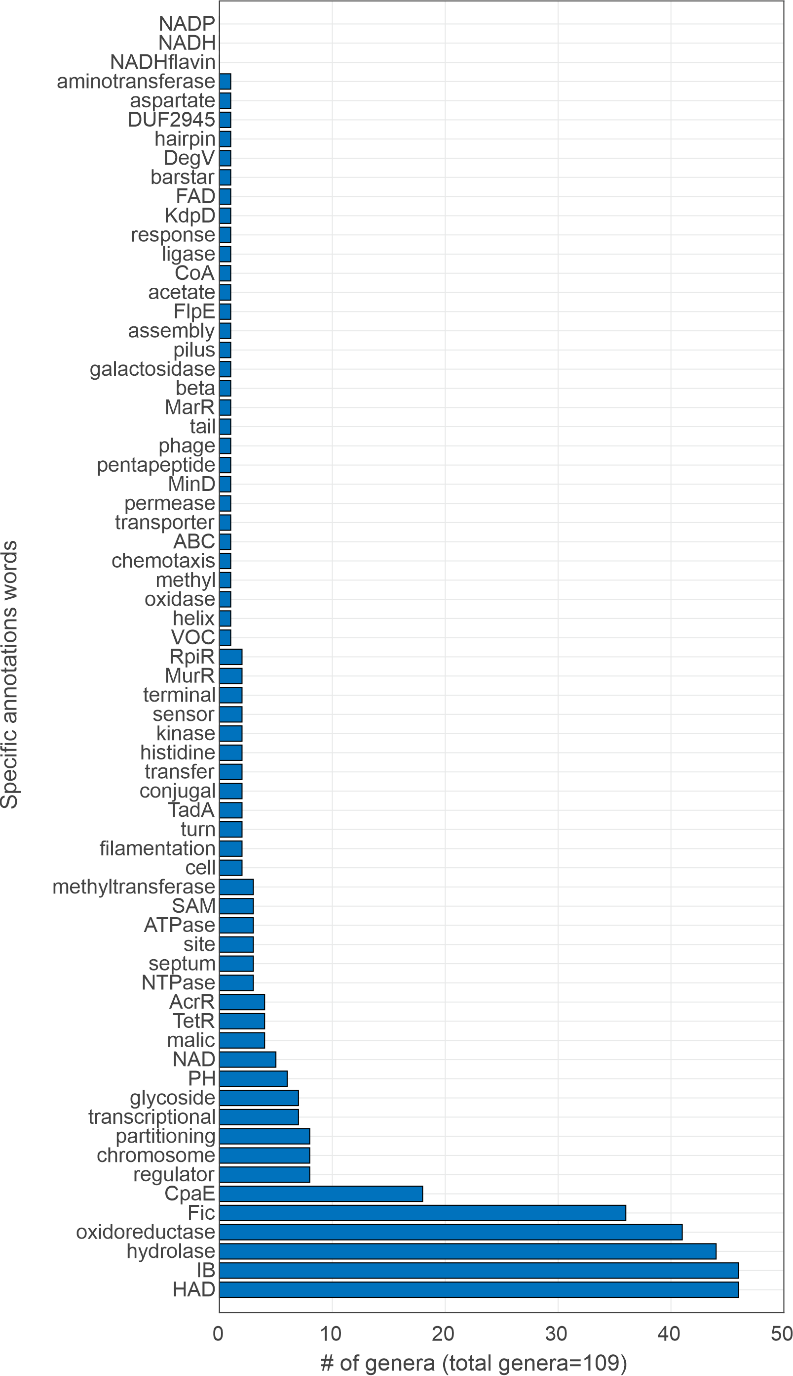
Supplementary Figure S3**. Specific words and their occurrence in synteny annotations of 708 Ms1 IGRs in 109 genera identified by the first iterative synteny search. Average occurrence of specific words was in 5.4 genera, resulting in 12 most occurring specific words starting 'HAD' up to 'PH' (see y-axis) used for the update the original phrases in Table 2. 7 of the most occurring specific words were the same as before (Supplementary Figure S2). 'transcriptional' and 'regulator' were semantically bound through 'TetR/AcrR family transcriptional regulator' or 'MurR/RpiR family transcriptional regulator' annotations, forming 'transcriptional regulator' phrase (Table 3). 'partitioning' and 'chromosome' originated from 'chromosome partitioning protein' annotation, forming a phrase 'chromosome partitioning'. Phrase 'PH' originated from 'PH domain-containing protein' annotation.

**Supplementary Figure S4.** Specific words and their occurrence in synteny annotations of finally obtained 824 Ms1 IGRs from 146 *Actinobacteria* genera.


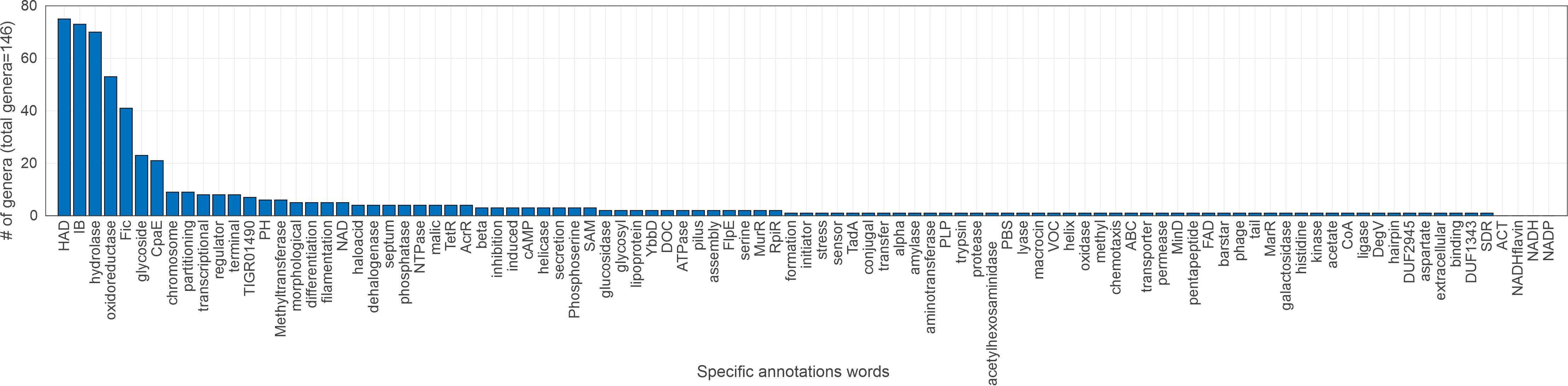


**
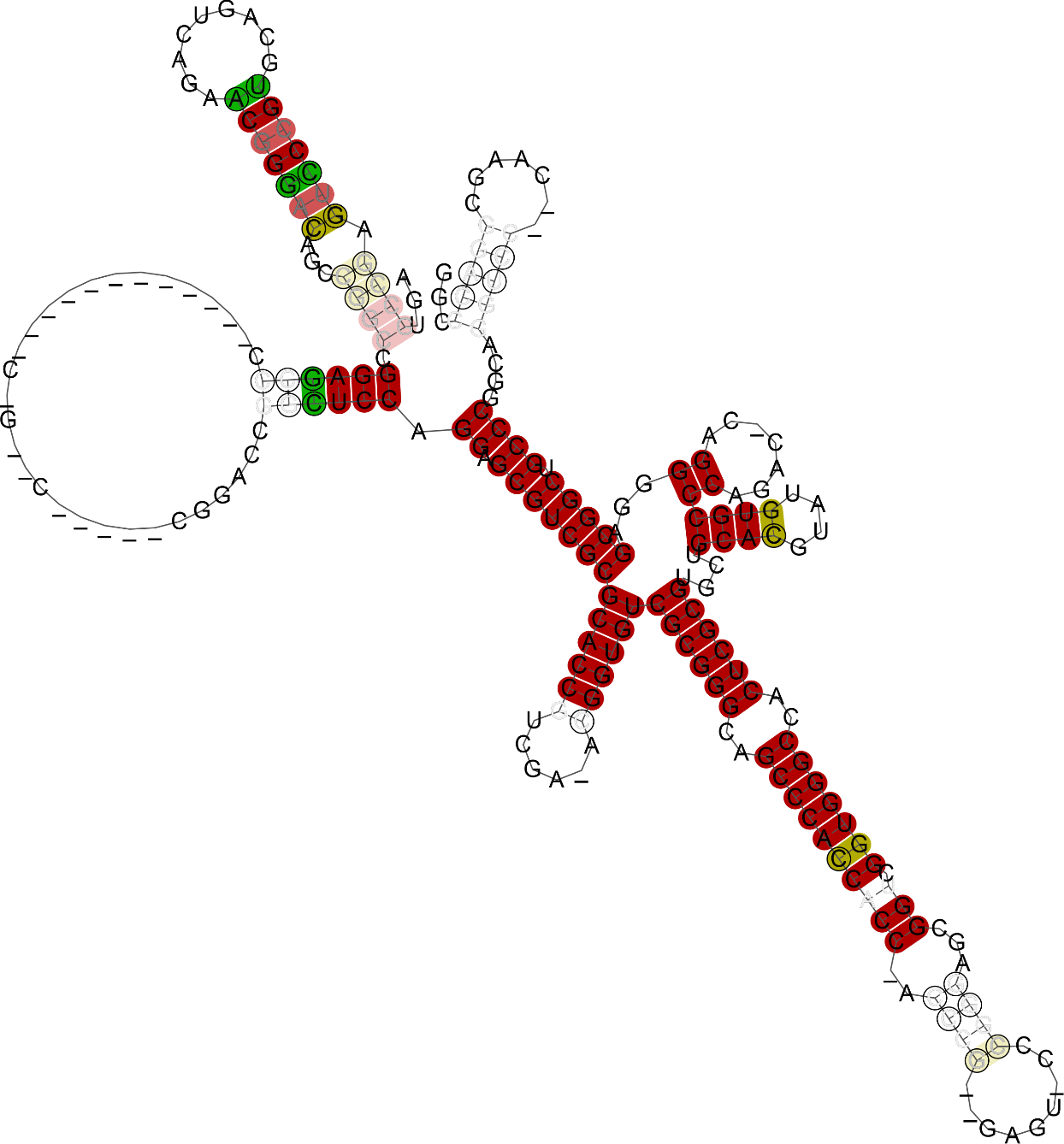
Supplementary Figure S5**. A consensus secondary structure of Str11 (scr1506). The structure was created by RNAalifold [1] from ClustalW2 [2] multiple sequence alignment of sequences from 42 *Streptomyces* species identified by BLAST search of *S. coelicolor* Str11 sequence in nt NCBI nucleotide database.

**
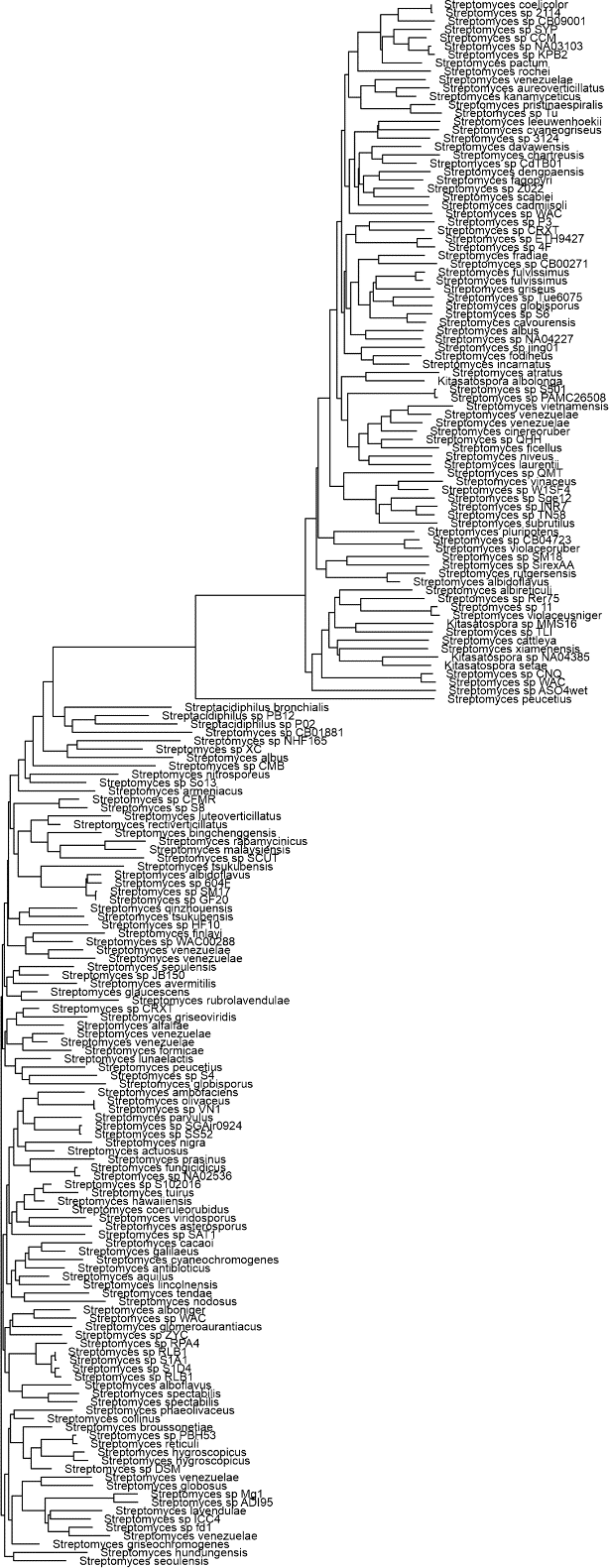
Supplementary Figure S6**. A phylogenetic tree of Ms1 IGRs identified in 188 *Streptomyces* species. The tree was generated using ClustalW2. The tree leaves were denoted only with genus and species names. Other details as strain identifiers were left out for readability of the tree.

**Supplementary Table S2**. List of 824 species, in which Ms1 RNAs were predicted in *Actinobacteria*. *Streptomyces* species were not included as they are shown in Supplementary Figure S1 unless found by sequence similarity to the synteny hits. Columns show: genomic sequence ID, species name, genomic loci of BLAST hits with sequence similarity to predicted Ms1 RNAs in evolutionarily related species and genomic locus of IGR with a predicted Ms1 RNA.

| ID | name | BLAST hit genomic locus | IGR genomic locus |
| --- | --- | --- | --- |
| CP023445.1 | Actinosynnema pretiosum X47 | 275876:275630 | 275382:275972 |
| CP029607.1 | Actinosynnema pretiosum subsp. pretiosum ATCC 31280 | 280450:280204 | 279961:280546 |
| CP024894.1 | Amycolatopsis sp. AA4 | 7906792:7907038 | 7906664:7907296 |
| CP060131.1 | Pseudonocardia sp. CGMCC 4.1532 | 1258061:1257817 | 1257783:1258119 |
| CP016077.1 | Actinoalloteichus sp. GBA129-24 | 299336:299089 | 298803:299520 |
| LN850107.1 | Alloactinosynnema sp. L-07 Alloactinosynnema sp. L-07 I | 6195183:6194939 | 6194735:6195363 |
| HE804045.1 | Saccharothrix espanaensis DSM 44229 | 315710:315466 | 315268:315846 |
| NZ_CP045480.1 | Amycolatopsis sp. YIM 10 4e-16 | 464448:464708 | 464128:464795 |
| CP016353.1 | Prauserella marina DSM 45268 | 6319114:6319359 | 6318982:6319527 |
| CP064192.1 | Saccharothrix sp. 6-C | 8740658:8740412 | 8740080:8740794 |
| CP054925.1 | Amycolatopsis sp. Hca4 | 6745264:6745018 | 6744621:6745411 |
| NZ_KB913032.1 | Amycolatopsis alba DSM 44262 scaffold1 2e-07 | 3176975:3176700 | 3176581:3177158 |
| CP022521.1 | Actinoalloteichus hoggarensis DSM 45943 | 289476:289229 | 288923:289661 |
| CP016793.1 | Lentzea guizhouensis DHS C013 | 2553820:2554067 | 2553599:2554309 |
| CP012752.1 | Kibdelosporangium phytohabitans KLBMP1111 | 375401:375153 | 374633:375556 |
| CP053564.1 | Pseudonocardia sp. Gen01 | 1179901:1179654 | 1179616:1179960 |
| CP009110.1 | Amycolatopsis methanolica 239 | 6769674:6769921 | 6769585:6770105 |
| CP012184.1 | Pseudonocardia sp. EC080619-01 | 2445161:2444914 | 2444868:2445307 |
| CP001736.1 | Kribbella flavida DSM 17836 | 567915:567696 | 567680:567951 |
| LT629732.1 | Actinopolymorpha singaporensis DSM 22024 I | 2013151:2012919 | 2012737:2013134 |
| CP049867.1 | Nocardioides sp. HDW12B | 3401637:3401880 | 3401595:3401933 |
| AP012204.1 | Microlunatus phosphovorus NM-1 DNA | 5295527:5295766 | 5295327:5295783 |
| CP025581.1 | Nocardioides houyundeii 78 | 3417894:3418121 | 3417834:3418143 |
| CP033324.1 | Nocardioides sp. 603 | 3157786:3158030 | 3157186:3158414 |
| AP022871.1 | Phytohabitans suffuscus NBRC 105367 DNA | 4056387:4056148 | 4056138:4055653 |
| CP046122.1 | Microlunatus sp. Gsoil 973 | 3298334:3298570 | 3297965:3298590 |
| LT629749.1 | Friedmanniella luteola DSM 21741 I | 271109:270874 | 270839:271345 |
| LT629772.1 | Microlunatus soli DSM 21800 I | 2351268:2351505 | 2350894:2351515 |
| CP041692.1 | Microlunatus sp. KUDC0627 | 816251:816015 | 815744:816568 |
| CP009896.1 | Pimelobacter simplex VKM Ac-2033D | 342461:342222 | 342195:342486 |
| CP038436.1 | Nocardioides seonyuensis MMS17-SY207-3 | 1274084:1274324 | 1274080:1274328 |
| CP060713.1 | Nocardioides mesophilus KACC 16243 | 2170430:2170196 | 2170186:2170456 |
| CP059164.1 | Nocardioides ungokensis LMG 28591 | 441392:441153 | 441142:441394 |
| CP000509.1 | Nocardioides sp. JS614 | 387431:387189 | 386220:387475 |
| CP040748.1 | Nocardioides sp. dk3136 | 3263693:3263929 | 3263651:3263963 |
| CP049866.1 | Nocardioides sp. HDW12A | 683296:683540 | 683283:683551 |
| CP038267.1 | Nocardioides euryhalodurans MMS17-SY117 | 2158069:2158307 | 2158055:2158428 |
| LT629757.1 | Marmoricola scoriae DSM 22127 I | 2587314:2587554 | 2587280:2587600 |
| CP059259.1 | Nocardioidaceae bacterium isolate SSC3 | 464425:464666 | 464458:464799 |
| LT629688.1 | Auraticoccus monumenti MON 2.2 I | 187327:187559 | 187182:187589 |
| CP033729.1 | Actinobacteria bacterium YIM 96077 | 508618:508383 | 508316:508625 |
| LT629791.1 | Jiangella alkaliphila DSM 45079 I | 695996:696236 | 696005:696261 |
| LT629771.1 | Jiangella sp. DSM 45060 I | 6975474:6975234 | 6975101:6975465 |
| CP001738.1 | Thermomonospora curvata DSM 43183 | 5489394:5489629 | 5488756:5490245 |
| CP032402.1 | Thermomonospora amylolytica YIM 77502 | 6609043:6609278 | 6608183:6609310 |
| CP036455.1 | Streptomonospora sp. M2 | 176676:176917 | 176262:177224 |
| CP031320.1 | Streptomyces armeniacus ATCC 15676 | 3246694:3246457 | 3246435:3246786 |
| LT559118.1 | Nonomuraea sp. ATCC 39727 isolate nono1 I | 1037878:1037644 | 0:0 |
| CP017717.1 | Nonomuraea sp. ATCC 55076 | 9673564:9673798 | 9674581:9673814 |
| CP045572.1 | Nonomuraea nitratireducens WYY166 | 410532:410295 | 410265:410633 |
| CP029711.1 | Streptosporangium sp. _caverna_ | 46893:46655 | 46625:47848 |
| CP001814.1 | Streptosporangium roseum DSM 43021 | 479835:479600 | 479260:480519 |
| CP001874.1 | Thermobispora bispora DSM 43833 | 254817:254581 | 253843:255271 |
| AP022870.1 | Phytohabitans flavus NBRC 107702 DNA | 7996830:7996586 | 7996569:7996841 |
| CP022753.1 | Nocardiopsis gilva YIM 90087 | 616361:616603 | 615813:616757 |
| CP006272.1 | Actinoplanes friuliensis DSM 7358 | 392931:392691 | 392680:392989 |
| LT629711.1 | Phycicoccus dokdonensis DSM 22329 I | 3602372:3602612 | 3602326:3603201 |
| LR134501.1 | Nocardiopsis dassonvillei NCTC10488 1 | 4300251:4300499 | 4299702:4300776 |
| CP046121.1 | Tetrasphaera sp. HKS02 | 2422696:2422458 | 2422104:2422730 |
| CP060712.1 | Phycicoccus endophyticus JCM 31784 | 2369196:2368958 | 2368588:2369199 |
| CP031194.1 | Streptomyces paludis GSSD-12 | 3171721:3171952 | 3171608:3171992 |
| CP063232.1 | Thermobifida fusca UPMC 901 | 1086203:1086443 | 1085834:1086559 |
| CP000088.1 | Thermobifida fusca YX | 153146:153386 | 0:0 |
| CP049935.1 | Phycicoccus sp. HDW14 | 3949084:3949321 | 3949026:3950231 |
| CP053707.1 | Arthrobacter sp. NEB 688 | 1362445:1362681 | 1362415:1363371 |
| CP041616.1 | Ornithinimicrobium sp. H23M54 | 3847534:3847296 | 3846993:3847529 |
| CP031447.1 | Austwickia chelonae LK16-18 | 494728:494963 | 494715:495362 |
| CP064985.1 | Austwickia sp. isolate Fred_18-Q3-R57-64_BATAC.85v2 | 738428:738192 | 737718:738511 |
| CP005929.1 | Actinoplanes sp. N902-109 | 474003:473765 | 473762:473991 |
| LT629758.1 | Actinoplanes derwentensis DSM 43941 I | 7196422:7196662 | 7196202:7196924 |
| AP012319.1 | Actinoplanes missouriensis 431 DNA | 329779:329537 | 329534:329800 |
| AP019371.1 | Actinoplanes sp. OR16 DNA | 2596823:2596579 | 2596574:2596841 |
| CP023865.1 | Actinoplanes teichomyceticus ATCC 31121 | 292747:292507 | 292488:293105 |
| CP023298.1 | Actinoplanes sp. SE50 | 422558:422322 | 422129:423123 |
| AP023355.1 | Actinocatenispora thailandica NBRC 105041 DNA | 213014:212779 | 212540:213116 |
| CP015163.1 | Amycolatopsis albispora WP1 | 5803799:5804044 | 5803687:5804356 |
| LN877229.1 | Kibdelosporangium sp. MJ126-NF4 | 6538888:6538643 | 0:0 |
| CP041306.1 | Amycolatopsis sp. Poz14 | 1220523:1220277 | 0:0 |
| CP034550.1 | Saccharothrix syringae NRRL B-16468 | 317899:317653 | 317453:318024 |
| CP007155.1 | Kutzneria albida DSM 43870 | 328239:327991 | 327985:328452 |
| CP069353.1 | Saccharopolyspora erythraea NRRL 23338 | 485511:485267 | 484264:485622 |
| CP038101.1 | Saccharomonospora sp. 31sw | 4293791:4294039 | 4293708:4294179 |
| NZ_CP045929.1 | Saccharopolyspora coralli E2A 1e-23 | 310730:311004 | 310549:311094 |
| CP001683.1 | Saccharomonospora viridis DSM 43017 | 3916948:3917196 | 3916855:3917461 |
| NZ_LT629701.1 | Allokutzneria albata DSM 44149 I 9e-19 | 6577109:6577384 | 6576693:6577483 |
| CP025990.1 | Actinoalloteichus sp. AHMU CJ021 | 2311565:2311808 | 2311501:2312342 |
| CP002593.1 | Pseudonocardia dioxanivorans CB1190 | 448050:448292 | 447912:448397 |
| NZ_CP040605.1 | Saccharopolyspora sp. ASAGF58 1e-29 | 2875598:2875882 | 2875360:2875966 |
| NZ_CP031142.1 | Saccharopolyspora pogona NRRL30141 1e-30 | 6147603:6147328 | 6147235:6147838 |
| NZ_PJNB01000001.1 | Saccharopolyspora spinosa DSM 44228 Ga0074763_11 2e-32 | 1347327:1347611 | 1347102:1347703 |
| CP014859.1 | Actinoalloteichus hymeniacidonis HPA177T _DSM 45092T | 294856:294610 | 294406:295031 |
| NZ_CP022752.1 | Actinopolyspora erythraea YIM 90600 5e-79 | 307994:308270 | 307607:308360 |
| AP018920.1 | Pseudonocardia autotrophica NBRC 12743 DNA | 484940:485186 | 484762:485196 |
| CP013854.1 | Pseudonocardia sp. HH130630-07 | 1916925:1916678 | 1916653:1917068 |
| CP011868.1 | Pseudonocardia sp. HH130629-09 | 4973558:4973313 | 4973296:4972544 |
| CP011862.1 | Pseudonocardia sp. AL041005-10 | 3940600:3940848 | 3940154:3939969 |
| CP001737.1 | Nakamurella multipartita DSM 44233 | 799218:798980 | 798423:799733 |
| CP050902.1 | Nocardioides sp. JQ2195 | 3749594:3749842 | 3749590:3749910 |
| CP038462.1 | Nocardioides daphniae JCM 16608 | 3291364:3291607 | 3291299:3291675 |
| CP040695.2 | Nocardioides sp. S-1144 | 399891:399641 | 399624:399902 |
| CP049257.1 | Nocardioides anomalus HKS04 | 4186364:4186120 | 4186105:4186365 |
| CP015079.1 | Nocardioides dokdonensis FR1436 | 1636696:1636447 | 1636404:1636721 |
| AP019307.1 | Nocardioides baekrokdamisoli KCTC 39748 DNA | 2020661:2020915 | 2020652:2020938 |
| CP060587.1 | Aeromicrobium sp. zg-629 | 176396:176157 | 176138:176362 |
| CP026952.1 | Aeromicrobium sp. 592 | 304101:303862 | 303824:304121 |
| CP027482.1 | Aeromicrobium sp. A1-2 | 2878001:2878240 | 2877991:2878327 |
| CP045737.1 | Aeromicrobium sp. MF47 | 289247:289009 | 288964:289267 |
| LT796768.1 | Aeromicrobium choanae 9H-4 I | 1393541:1393780 | 1393562:1393815 |
| LT629799.1 | Friedmanniella sagamiharensis DSM 21743 I | 4155411:4155179 | 4155098:4155533 |
| LT629710.1 | Nakamurella panacisegetis P4-7KCTC 19426CECT 7604 I | 2553372:2553127 | 2552953:2552840 |
| CP060298.1 | Nakamurella sp. PAMC28650 | 1683530:1683289 | 1683009:1684132 |
| CP034170.1 | Nakamurella sp. s14-144 | 508957:508720 | 508417:509408 |
| CP022434.1 | Nocardiopsis dassonvillei HZNU_N_1 | 183738:183986 | 183205:184264 |
| CP017965.1 | Nocardiopsis dassonvillei NOCA502F | 183643:183891 | 183094:184168 |
| CP054933.1 | Nocardiopsis flavescens NA01583 | 194845:195094 | 194338:195319 |
| CP041763.1 | Gordonia sp. HY186 | 2935841:2936130 | 2935775:2936160 |
| CP046320.1 | Gordonia bronchialis FDAARGOS_676 | 4547805:4548090 | 4547699:4548291 |
| CP023405.1 | Gordonia sp. 1D | 446192:445907 | 445820:446840 |
| CP022580.1 | Gordonia rubripertincta CWB2 | 559827:559543 | 559455:559563 |
| CP059694.1 | Gordonia rubripertincta SD5 | 1543146:1542862 | 1542773:1543809 |
| CP052884.1 | Gordonia ajococcus A2 | 447641:447356 | 447269:448289 |
| CP054691.1 | Gordonia sp. X0973 | 344407:344122 | 344104:344566 |
| CP047235.1 | Gordonia sp. JH63 | 586787:586503 | 586402:587358 |
| CP046257.1 | Gordonia sp. 135 | 527477:527192 | 527104:528142 |
| CP049836.1 | Gordonia terrae RL-JC02 | 5013304:5013588 | 5012733:5013689 |
| CP045810.1 | Gordonia amarae ATCC 27808 | 572569:572284 | 0:0 |
| CP033972.1 | Gordonia insulae MMS17-SY073 | 4371409:4371124 | 4371072:4371648 |
| CP029604.1 | Gordonia terrae NRRL B-16283 | 637698:637414 | 637314:638268 |
| CP002907.1 | Gordonia sp. KTR9 | 491434:491150 | 491051:491921 |
| CP059491.1 | Gordoniaceae bacterium zg-686 | 525708:525422 | 525401:526350 |
| CP045809.1 | Gordonia amarae DSM 43602 | 4663189:4663473 | 0:0 |
| CP003119.1 | Gordonia polyisoprenivorans VH2 | 895644:895360 | 895243:895768 |
| CP027433.1 | Gordonia iterans Co17 | 643106:642823 | 642629:643137 |
| CP025435.1 | Gordonia sp. YC-JH1 | 970806:970511 | 970454:970873 |
| CP011853.1 | Gordonia phthalatica QH-11 | 447791:447500 | 447348:447858 |
| CP001966.1 | Tsukamurella paurometabola DSM 20162 | 4026137:4026409 | 4026041:4026479 |
| LR134469.1 | Tsukamurella tyrosinosolvens NCTC13231 plasmid 27 | 64436:64716 | 64343:64805 |
| CP019066.1 | Tsukamurella tyrosinosolvens MH1 | 4532801:4533081 | 4532708:4533170 |
| LR131273.1 | Tsukamurella paurometabola NCTC10741 1 | 1971738:1971459 | 1971399:1971831 |
| NC_015671.1 | Cellulomonas gilvus ATCC 13127 4e-127 | 2931450:2931763 | 2931449:2931764 |
| NZ_BJUA01000004.1 | Cellulomonas persica NBRC 101101 04 sequence 2e-51 | 215342:215044 | 214758:215399 |
| NZ_BMOJ01000004.1 | Cellulomonas gelida JCM 1490 04 sequence 5e-50 | 148291:148554 | 148270:148666 |
| NZ_CP021430.1 | Cellulomonas sp. PSBB021 1e-49 | 2260156:2260426 | 2260137:2260475 |
| NZ_BJLP01000009.1 | Cellulomonas uda NBRC 3747 009 sequence 2e-47 | 43503:43234 | 43185:43523 |
| NZ_JAHBOH010000001.1 | Cellulomonas sp. DKR-3 contig1 5e-46 | 1696762:1696500 | 1696431:1696775 |
| NZ_JEOE01000013.1 | Cellulomonas sp. HZM contig13 4e-41 | 42206:41947 | 41931:42251 |
| NZ_BJWG01000011.1 | Cellulomonas composti NBRC 100758 11 sequence 2e-39 | 75537:75265 | 75238:75540 |
| NZ_RKHX01000001.1 | Cellulomonas sp. PhB150 Ga0304843_11 5e-38 | 1429138:1429403 | 1429130:1429410 |
| NZ_BKAL01000011.1 | Cellulomonas soli NBRC 109434 11 sequence 4e-37 | 130876:130619 | 130562:130877 |
| NZ_CP048210.1 | Cellulomonas sp. H30R-01 3e-36 | 2748595:2748876 | 2748475:2748952 |
| NZ_JAHKSJ010000002.1 | Cellulomonas aurantiaca THG-SMD2.3 contig2 8e-36 | 571794:571493 | 571442:571914 |
| NZ_CABKRU010000001.1 | Cellulomonas massiliensis isolate MGYG-HGUT-01416 2e-34 | 1546669:1546456 | 1546400:1546785 |
| NZ_JACYFR010000001.1 | Cellulomonas sp. JH27-2 contig1 2e-34 | 197250:197508 | 197246:197537 |
| NZ_QWKP01000218.1 | Cellulomonas rhizosphaerae NEAU-TCZ24 scf_218 5e-34 | 23969:23699 | 23588:24004 |
| NZ_BONP01000012.1 | Cellulomonas phragmiteti NBRC 110785 12 sequence 3e-32 | 53336:53080 | 53048:53346 |
| NZ_LMFC01000001.1 | Cellulomonas sp. Root485 contig_1 2e-31 | 158470:158296 | 158003:158559 |
| NZ_BONK01000010.1 | Cellulomonas chitinilytica NBRC 110799 10 sequence 4e-30 | 10430:10178 | 10128:10468 |
| NZ_BJUB01000002.1 | Cellulomonas xylanilytica NBRC 101102 02 sequence 4e-30 | 52081:52261 | 51993:52411 |
| NC_014151.1 | Cellulomonas flavigena DSM 20109 4e-30 | 3112997:3113235 | 3112980:3113293 |
| NZ_LMQI01000003.1 | Cellulomonas sp. Leaf395 contig_3 4e-30 | 122742:122570 | 122553:122835 |
| NC_015514.1 | Cellulomonas fimi ATCC 484 3e-29 | 916141:915885 | 915782:916308 |
| NZ_BONN01000001.1 | Cellulomonas oligotrophica NBRC 109435 01 sequence 9e-29 | 546415:546184 | 546157:546452 |
| NZ_LMOO01000001.1 | Cellulomonas sp. Leaf334 contig_1 9e-29 | 1627367:1627541 | 1627278:1627545 |
| NZ_BIMR01000353.1 | Cellulomonas biazotea NBRC12680 353 sequence 7e-28 | 1187:1443 | 1103:1535 |
| NZ_BJWH01000001.1 | Cellulomonas terrae NBRC 100819 01 sequence 7e-28 | 387430:387604 | 387341:387608 |
| NZ_AUEW01000012.1 | Cellulomonas sp. URHD0024 I847DRAFT_scaffold00010.10_C 7e-28 | 151183:150937 | 150897:151224 |
| NZ_BCRB01000091.1 | Cellulomonas iranensis NBRC 101100 _ JCM 18110 NBRC 101100 6e-27 | 11550:11312 | 11307:11592 |
| NZ_SPHX01000008.1 | Cellulomonas sp. HD19AZ1 Scaffold8_1 4e-26 | 70514:70300 | 70269:70556 |
| NZ_QOHN01000267.1 | Cellulomonas iranensis LZ-P1 contig267 9e-25 | 651:442 | 406:693 |
| NZ_NFZP01000037.1 | Cellulomonas iranensis SID13905 NODE_37_length_35857_cov_213.993_ID_73 3e-24 | 2101:2315 | 2059:2346 |
| NZ_QXFN01000003.1 | Cellulomonas telluris CPCC 204705 Scaffold3 2e-22 | 103930:103715 | 103705:104072 |
| NZ_CP045245.1 | Cellulomonas sp. JZ18 2e-22 | 861135:860909 | 860905:861248 |
| NZ_CP074404.1 | Cellulomonas sp. zg-ZUI188 4e-22 | 3060451:3060660 | 3060316:3060662 |
| NZ_QEES01000002.1 | Cellulomonas sp. WB94 Seq0 4e-22 | 908714:908961 | 908705:908970 |
| NZ_JAGFBN010000002.1 | Cellulomonas sp. zg-ZUI168 Scaffold2_1 1e-21 | 706255:706467 | 706120:706469 |
| NZ_JACHVX010000002.1 | Cellulomonas cellasea RAS26 Ga0372465_02 1e-21 | 181192:180946 | 180930:181336 |
| NZ_BJLR01000004.1 | Cellulomonas cellasea NBRC 3753 04 sequence 3e-21 | 71786:72081 | 71696:72100 |
| NZ_FOKA01000005.1 | Cellulomonas marina CGMCC 4.6945 2e-20 | 30137:30428 | 30136:30578 |
| NZ_RWJX01000091.1 | Cellulomonas endophytica SYSUP0004 SYSUP0004_Contig91 5e-19 | 1787:2037 | 1545:2047 |
| NZ_CP039291.1 | Cellulomonas shaoxiangyii Z28 1e-18 | 823293:823026 | 822969:823498 |
| NZ_VWSD01000172.1 | Cellulomonas citrea Ao-9 NODE_172_length_5653_cov_200.433044 2e-16 | 342:514 | 0:536 |
| NZ_BONO01000008.1 | Cellulomonas pakistanensis NBRC 110800 08 sequence 2e-15 | 18733:18472 | 18412:18766 |
| NZ_JABCJJ010000006.1 | Cellulomonas fimi SB NODE_6_length_172402_cov_89.658256 2e-15 | 124261:124102 | 123972:124316 |
| NZ_VEGH01000002.1 | Cellulosimicrobium cellulans DE0111 NODE_2_length_458829_cov_17.014138 1e-14 | 47776:48015 | 47718:48060 |
| NZ_JAHLPH010000002.1 | Cellulomonas hominis N95 No95_ctg002 1e-13 | 214093:213830 | 213800:214183 |
| NZ_JAAXOZ010000190.1 | Cellulomonas hominis ATCC BAA-786 W8037_190 1e-13 | 3661:3398 | 3368:3751 |
| NZ_BJVQ01000078.1 | Cellulomonas hominis NBRC 16055 078 sequence 3e-13 | 3054:3317 | 2964:3347 |
| NZ_SDWW01000083.1 | Cellulomonas sp. HLT2-17 NODE_83_length_7172_cov_92.745455 3e-13 | 5900:6112 | 5805:6257 |
| NZ_SZYE01000260.1 | Cellulomonas hominis CS1 NODE_260_length_4401_cov_37.584698 3e-13 | 3222:2993 | 2947:3281 |
| NZ_CACRYJ010000044.1 | Occultella aeris CIP 111667 8e-13 | 87072:87309 | 86965:87368 |
| NZ_FCOT01000005.1 | Cellulomonas timonensis SN7 8e-13 | 1245:1457 | 1158:1565 |
| NZ_CP041203.1 | Cellulomonas sp. Y8 2e-12 | 2496738:2496445 | 2496436:2496748 |
| NZ_AXCW01000386.1 | Actinotalea ferrariae CF5-4 contig413 6e-12 | 1866:2102 | 1747:2108 |
| NZ_WMHQ01000022.1 | Ruania sp. HY044 Scaffold19_1 6e-12 | 49410:49172 | 49123:49527 |
| NZ_JAHVCI010000089.1 | Cellulomonas sp. PS-H5 Scaffold89 1e-10 | 5401:5107 | 5098:5411 |
| NZ_JACSQQ010000011.1 | Oerskovia sp. Sa4CUA1 NODE_11_length_126116_cov_7.180047 4e-10 | 49912:49694 | 49679:50071 |
| NZ_CP042174.1 | Oerskovia sp. KBS0722 4e-10 | 1479075:1478881 | 1478744:1479234 |
| NZ_JAEINH010000013.1 | Sanguibacter sp. YZGR15 Scaffold13 1e-09 | 12283:12123 | 12109:12327 |
| NZ_JAGXJG010000016.1 | Cellulomonas sp. GbtcB1 B1_16 3e-09 | 64283:64526 | 64222:64535 |
| NZ_JACVQL010000221.1 | Actinotalea ferrariae SN-202-OC-R2 S_N-202-OC-R2_contig_221 3e-09 | 349:101 | 0:403 |
| NZ_ATWL01000009.1 | Ruania albidiflava DSM 18029 K338DRAFT_scaffold00009.9_C 8e-09 | 73366:73170 | 73046:73547 |
| NZ_BMEB01000002.1 | Cellulomonas carbonis CGMCC 1.10786 02 sequence 1e-06 | 716094:715906 | 715872:716147 |
| NZ_PDJG01000001.1 | Sanguibacter antarcticus DSM 18966 Ga0074699_11 1e-06 | 3323715:3323550 | 3323472:3323788 |
| NZ_MWLL01000270.1 | Kineosporia sp. R_H_3 ECOMGDLI_270 4e-06 | 16405:16254 | 16182:16534 |
| NZ_JAGEMK010000009.1 | Actinotalea sp. BY-33 NODE_9_length_165195_cov_75.684918 4e-06 | 135870:136059 | 135793:136075 |
| NZ_MAQA01000043.1 | Oerskovia enterophila DSM 43852 OERS_contig000043 4e-06 | 24987:24759 | 24696:25111 |
| NZ_BKBA01000008.1 | Knoellia locipacati NBRC 109775 08 sequence 1e-05 | 194880:195033 | 194793:195327 |
| NZ_PJNE01000001.1 | Phycicoccus duodecadis DSM 12806 Ga0074712_11 1e-05 | 1430606:1430442 | 1430059:1430684 |
| NZ_AXCZ01000210.1 | Cellulomonas bogoriensis 69B4 _ DSM 16987 69B4 contig268 3e-05 | 691:486 | 482:772 |
| NZ_JABBXA010000002.1 | Microbispora sp. H13382 NODE_2 3e-05 | 735969:735730 | 735601:736527 |
| NZ_LMSE01000004.1 | Tetrasphaera sp. Soil756 contig_4 8e-05 | 82612:82766 | 82534:83174 |
| NZ_JABBWU010000003.1 | Microbispora sp. H10836 NODE_3 8e-05 | 29120:28885 | 28753:29678 |
| NZ_VSFF01000005.1 | Actinomadura syzygii GKU157 NODE_5_length_594850_cov_52.741540 0.0002 | 285375:285218 | 285153:284533 |
| NZ_VFPO01000001.1 | Actinomadura hallensis DSM 45043 Ga0197561_11 0.0002 | 6427269:6427421 | 6428046:6427429 |
| NZ_ML769314.1 | Catellatospora paridis NEAU-CL2 Scaffold3 0.0002 | 815942:816011 | 0:0 |
| NZ_VPFI01000008.1 | Ruania sp. HY211 Scaffold8 0.0002 | 39919:39741 | 39621:40074 |
| NZ_VFMN01000001.1 | Lapillicoccus jejuensis DSM 18607 Ga0197574_11 0.0006 | 985421:985275 | 985112:985539 |
| NZ_LAXD01000001.1 | Carbonactinospora thermoautotrophica H1 H1_ 0.0006 | 447405:447250 | 447193:447704 |
| NZ_JABVEC010000050.1 | Actinomadura alba HBUM206468 contig50 2e-18 | 9782:9921 | 9046:10003 |
| NZ_UAPP01000009.1 | Actinomadura madurae NCTC11373 1e-14 | 925803:926001 | 925093:926007 |
| NZ_VJYI01000002.1 | Nonomuraea sp. SYSU D8015 Scaffold2_1 0.002 | 336992:337164 | 337953:337174 |
| NZ_JACHMV010000001.1 | Actinomadura catellatispora DSM 44772 Ga0373993_01 0.018 | 1001226:1001081 | 1000551:1001228 |
| NZ_QFXK01000034.1 | Kribbella monticola NEAU-SW521 scaffold41 0.013 | 277031:277118 | 277042:277204 |
| NZ_JAHDTF010000005.1 | Sphaerisporangium sp. H8589 Scaffold4_1 8e-10 | 224230:224367 | 223604:224409 |
| NZ_KB889692.1 | Embleya scabrispora DSM 41855 A3ICDRAFT_scaffold_45.46 0 | 76094:76522 | 76094:76522 |
| NZ_QOIL01000008.1 | Sphaerisporangium album CCTCC AA 208026 scaffold8 1e-13 | 224165:224008 | 223654:224970 |
| NZ_WNXX01000013.1 | Actinotalea caeni JCM 30447 Scaffold13_1 0.036 | 3365:3547 | 3134:3640 |
| NZ_JABWGO010000010.1 | Nonomuraea rhodomycinica TBRC6557 NODE_10_length_373004_cov_26.5151 0.036 | 40942:40790 | 40727:40332 |
| NZ_VSRQ01000002.1 | Actinomadura decatromicini CYP1-5 NODE_2 2e-69 | 466500:466345 | 466279:465659 |
| NZ_VSFG01000001.1 | Actinomadura chibensis JCM 14158 NODE_1_length_2620600_cov_56.617746 2e-62 | 1825282:1825437 | 1826123:1825503 |
| NZ_BCQS01000003.1 | Actinomadura latina NBRC 106108 1e-14 | 223004:222831 | 222785:223688 |
| NZ_JABXFD010000339.1 | Actinomadura sp. BRA 177 NODE_99_length_24498_cov_21.7503_ID_197 4e-14 | 10306:10161 | 10139:11032 |
| NZ_SMKT01000176.1 | Actinomadura sp. KC06 NODE_176_length_17143_cov_33.7064 8e-16 | 591:736 | 0:756 |
| NZ_BDDE01000028.1 | Actinomadura sp. K4S16 NBRC 110471 0.011 | 130412:130264 | 130255:129638 |
| NZ_SMKY01000224.1 | Actinomadura darangshiensis DSM 45941 NODE_224_length_14916_cov_21.3001 6e-11 | 598:742 | 0:755 |
| NZ_VCKW01000192.1 | Actinomadura sp. 14C53 NODE_192_length_16823_cov_13.467 6e-11 | 16180:16037 | 16014:16823 |
| NZ_WBMT01000003.1 | Actinomadura rudentiformis HMC1 Scaffold3 8e-16 | 501784:501928 | 500758:502079 |
| NZ_JABVEB010000001.1 | Actinomadura sp. HBU206391 contig1 6e-51 | 7725:7867 | 6973:7942 |
| NZ_JAAGLI010000611.1 | Actinomadura bangladeshensis SID10258 contig-6000018 2e-50 | 10785:10650 | 10635:10018 |
| NZ_BMRF01000012.1 | Actinomadura cremea JCM 3308 012 sequence 7e-50 | 191958:192090 | 191266:192133 |
| NZ_SMKU01000141.1 | Actinomadura rubrisoli H3C3 NODE_141_length_23355_cov_49.0153 8e-16 | 307:451 | 0:691 |
| NZ_WING01000001.1 | Actinomadura sp. J1-007 scf7180000000008 3e-15 | 3473974:3473826 | 3473740:3473243 |
| NZ_WOFH01000005.1 | Actinomadura litoris NEAU-AAG5 Scaffold5 2e-49 | 235701:235560 | 235518:236427 |
| NZ_FZOR01000042.1 | Actinomadura meyerae DSM 44715 2e-49 | 44140:44287 | 43375:44291 |
| NZ_BCQU01000050.1 | Actinomadura rubrobrunea NBRC 15275 1e-13 | 41859:41712 | 40337:43814 |
| NZ_RBWU01000001.1 | Actinomadura pelletieri DSM 43383 Ga0170373_101 6e-18 | 656959:657105 | 657740:657114 |
| NZ_SMJX01000229.1 | Actinomadura sp. KC216 NODE_229_length_13796_cov_15.0358 7e-17 | 563:708 | 0:730 |
| NZ_SMKK01000152.1 | Actinomadura sp. 7K507 NODE_152_length_23724_cov_20.9352 8e-49 | 23149:23013 | 22953:23724 |
| NZ_QVNQ01000001.1 | Actinomadura sp. LHW52907 scaffold1 4e-14 | 480223:480077 | 480068:479454 |
| NZ_VCKZ01000406.1 | Actinomadura geliboluensis A8036 NODE_478_length_7905_cov_6.08113 3e-48 | 6192:6339 | 5447:6343 |
| NZ_CAACUZ010000004.1 | Actinomadura formosensis isolate LMG 29178 4e-13 | 52729:52886 | 52022:52918 |
| NZ_JAGEOJ010000038.1 | Actinomadura barringtoniae GKU 128 38 1e-47 | 22080:22218 | 22975:22340 |
| NZ_BCQT01000002.1 | Actinomadura macra NBRC 14102 1e-47 | 74044:74182 | 73306:74182 |
| NZ_AULB01000016.1 | Actinomadura rifamycini DSM 43936 H505DRAFT_scaffold00013.13_C 4e-47 | 165895:166027 | 165174:166066 |
| NZ_JADOUA010000001.1 | Actinomadura viridis DSM 43175 Ga0310409_01 4e-47 | 7202461:7202320 | 7202187:7203292 |
| NZ_JACCBT010000001.1 | Actinomadura citrea DSM 43461 Ga0104564_01 4e-47 | 8930746:8930881 | 8929993:8930906 |
| NZ_JACHMQ010000001.1 | Actinomadura coerulea DSM 43675 Ga0104566_01 4e-47 | 5824882:5825027 | 5824127:5825041 |
| NZ_SMKH01000374.1 | Actinomadura sp. KC345 NODE_374_length_6538_cov_15.1859 2e-12 | 578:722 | 0:752 |
| NZ_JACCBA010000001.1 | Actinomadura luteofluorescens DSM 40398 Ga0104561_01 1e-46 | 4788140:4788002 | 4787973:4788898 |
| NZ_CP054932.1 | Actinomadura sp. NAK00032 4e-46 | 9214562:9214697 | 9213828:9214713 |
| NZ_CP044407.1 | Actinomadura sp. WMMB 499 WMMB499 4e-46 | 4838868:4838736 | 4838686:4839570 |
| NZ_FZNP01000002.1 | Actinomadura mexicana DSM 44485 1e-45 | 446683:446819 | 445935:447128 |
| NZ_BMRO01000027.1 | Actinomadura livida JCM 3387 27 sequence 6e-11 | 15354:15497 | 16162:15545 |
| NZ_RFFG01000001.1 | Actinomadura harenae NEAU-Ht49 Scaffold1 2e-44 | 71397:71535 | 70605:71693 |
| NZ_SMKB01000257.1 | Actinomadura sp. 7K534 NODE_257_length_9655_cov_74.8759 2e-44 | 2126:2265 | 2916:2299 |
| NZ_SMLC01000058.1 | Actinomadura sp. 6K520 NODE_58_length_40705_cov_69.4341 2e-44 | 6940:7082 | 7734:7117 |
| NZ_SMKM01000061.1 | Actinomadura sp. GC306 NODE_61_length_27984_cov_19.0694 2e-44 | 22235:22095 | 21965:23005 |
| NZ_JAIBOA010000006.1 | Actinomadura sp. PM05-2 6 2e-44 | 58228:58087 | 58032:58892 |
| NZ_BCRO01000051.1 | Actinomadura hibisca NBRC 15177 9e-09 | 44674:44523 | 44503:45357 |
| NZ_BOOS01000024.1 | Sphaerisporangium krabiense NBRC 107571 024 sequence 6e-05 | 96125:95980 | 95706:96990 |
| NZ_CAACVB010000027.1 | Actinomadura roseirufa isolate LMG 30035 2e-17 | 1068:1211 | 1869:1252 |
| NZ_POUA01000698.1 | Spongiactinospora gelatinilytica 7K107 NODE_698_length_1599_cov_33.1841 2e-18 | 467:614 | 0:723 |
| NZ_QTTT01000001.1 | Thermomonospora umbrina DSM 43927 Ga0197485_11 2e-16 | 2820204:2820346 | 2821626:2820772 |
| NZ_WBMR01000097.1 | Actinomadura montaniterrae CYP1-1B Scaffold97 2e-11 | 14663:14519 | 14401:15464 |
| NZ_QURH01000200.1 | Actinomadura logoneensis NEAU-G17 scaffold180 3e-42 | 25211:25345 | 24429:26196 |
| NZ_QMEY01000001.1 | Spongiactinospora rosea LHW63015 scaffold1 1e-19 | 434970:434823 | 434714:433923 |
| NZ_JADBDZ010000001.1 | Actinomadura algeriensis DSM 46744 Ga0415221_01 3e-15 | 2246584:2246728 | 2245883:2246785 |
| NZ_QZEY01000031.1 | Bailinhaonella thermotolerans YIM 75507 Scaffold31 4e-13 | 38998:39142 | 38817:39410 |
| NZ_JACJIA010000002.1 | Actinomadura namibiensis DSM 44197 Ga0415225_02 3e-41 | 816419:816280 | 816269:817106 |
| NZ_JAGEPF010000033.1 | Actinomadura sp. LCR2-06 NODE_33_length_125903_cov_25.052832 3e-41 | 42791:42652 | 42588:43578 |
| NZ_JABMCA010000010.1 | Actinomadura sp. RB99 amrb99_10 3e-41 | 111922:111781 | 111708:112709 |
| NZ_JOFJ01000002.1 | Spirillospora albida NRRL B-3350 contig2.1 2e-12 | 129489:129344 | 129308:130221 |
| NZ_FNCN01000004.1 | Sinosporangium album CPCC 201354 2e-17 | 96498:96357 | 96253:97339 |
| NZ_BCQR01000025.1 | Actinomadura kijaniata NBRC 14229 4e-40 | 78079:78218 | 77392:78228 |
| NZ_CP053892.1 | Actinomadura verrucosospora NRRLB18236 3e-09 | 6285420:6285260 | 6285199:6286224 |
| NZ_BOOV01000022.1 | Sphaerisporangium siamense NBRC 107570 22 sequence 0.0007 | 185649:185504 | 185229:186133 |
| NZ_WBMS02000059.1 | Actinomadura physcomitrii LD22 Scaffold59 4e-14 | 26096:26242 | 25397:26275 |
| NZ_JAGEOK010000004.1 | Actinomadura nitritigenes L46 NODE_4_length_389425_cov_29.990760 4e-14 | 106459:106605 | 105675:106697 |
| NZ_BOOC01000005.1 | Microbispora corallina NBRC 16416 005 sequence 1e-13 | 438352:438500 | 437795:438535 |
| NZ_BOOW01000025.1 | Sinosporangium siamense NBRC 109515 25 sequence 6e-38 | 6003:6143 | 7016:6231 |
| NZ_JABBWZ010000020.1 | Microbispora sp. H10949 NODE_20 6e-38 | 56500:56655 | 55840:56772 |
| NZ_FNKK01000002.1 | Thermostaphylospora chromogena DSM 43794 3e-08 | 2063391:2063253 | 2063163:2064026 |
| NZ_BBYM01000005.1 | Microtetraspora niveoalba NBRC 15239 2e-12 | 108295:108440 | 107648:108616 |
| NZ_BOOJ01000014.1 | Planobispora siamensis NBRC 107568 014 sequence 1e-19 | 22306:22162 | 22088:22917 |
| NZ_JADBGC010000002.1 | Microbispora sitophila NEAU-D428 Scaffold2 1e-06 | 454770:454622 | 454436:455374 |
| NZ_FNVT01000004.1 | Nonomuraea solani CGMCC 4.7037 7e-37 | 251445:251556 | 250449:251650 |
| NZ_JADG01000007.1 | Actinomadura oligospora ATCC 43269 P696DRAFT_scaffold00005.5_C 7e-37 | 203800:203934 | 203011:204104 |
| NZ_JPMW01000001.1 | Planobispora rosea ATCC 53733 scaffold00001 7e-17 | 322151:322006 | 321751:322793 |
| NZ_LAVL01000007.1 | Nonomuraea sp. SBT364 scaffold7_size127390 7e-37 | 5315:5426 | 4741:5453 |
| NZ_BOOH01000058.1 | Planobispora longispora NBRC 13918 058 sequence 2e-16 | 71477:71332 | 71065:72285 |
| NZ_WVUI01000002.1 | Microbispora triticiradicis MPMI5 NODE_2_length_68922_cov_19.2235 6e-05 | 2415:2563 | 1775:2698 |
| NZ_JACHJJ010000001.1 | Planomonospora venezuelensis CECT 3303 Ga0436949_01 4e-20 | 347689:347544 | 347416:348293 |
| NZ_BOOR01000010.1 | Planotetraspora thailandica NBRC 104271 010 sequence 2e-12 | 274466:274616 | 273880:274769 |
| NZ_BOOB01000018.1 | Microbispora amethystogenes NBRC 101907 018 sequence 3e-36 | 43125:42968 | 42892:43793 |
| NZ_VFOZ01000001.1 | Actinoallomurus bryophytorum DSM 102200 Ga0264381_11 3e-36 | 3151328:3151461 | 3152287:3151502 |
| NZ_JNZQ01000005.1 | Microbispora rosea subsp. nonnitritogenes NRRL B-2631 contig5.1 9e-09 | 81060:80918 | 80793:81663 |
| NZ_BOOF01000029.1 | Microbispora siamensis NBRC 104113 029 sequence 1e-07 | 23708:23560 | 23370:24312 |
| NZ_FNVO01000009.1 | Thermomonospora echinospora DSM 43163 2e-17 | 299744:299889 | 298827:300106 |
| NZ_JACHIN010000001.1 | Nonomuraea endophytica DSM 45385 Ga0415315_01 9e-36 | 1094284:1094421 | 1093747:1094422 |
| NZ_MWJN01000008.1 | Microbispora sp. GKU 823 contig8 1e-07 | 57444:57592 | 56838:57782 |
| NZ_JABTEZ010000001.1 | Planomonospora sp. ID67723 NODE_1_length_2703498_cov_358.854063 9e-22 | 1214699:1214554 | 1214384:1215321 |
| NZ_BMNK01000012.1 | Nonomuraea glycinis CGMCC 4.7430 12 sequence 9e-36 | 51163:51026 | 51023:51716 |
| NZ_JAHDTI010000022.1 | Nonomuraea sp. H16431 Scaffold20_1 9e-36 | 4957:4846 | 4817:5426 |
| NZ_QNFZ01000479.1 | Nonomuraea lactucae NEAU-YG30 C3289 3e-35 | 707:569 | 506:1254 |
| NZ_JAFCNB010000018.1 | Microbispora sp. RL4-1S NODE_18_length_143829_cov_35.051565 2e-12 | 25767:25612 | 25500:26446 |
| NZ_BOOK01000025.1 | Planobispora takensis NBRC 109077 025 sequence 1e-19 | 5797:5941 | 5148:6138 |
| NZ_SMKP01000110.1 | Nonomuraea diastatica KC712 NODE_110_length_34550_cov_32.526 1e-34 | 33747:33637 | 33575:34550 |
| NZ_FTNI01000026.1 | Microbispora rosea ATCC 12950 1e-34 | 111723:111859 | 111107:111977 |
| NZ_BMRC01000004.1 | Nonomuraea spiralis JCM 3286 004 sequence 1e-34 | 119256:119401 | 118638:119408 |
| NZ_BOOT01000044.1 | Sphaerisporangium melleum NBRC 107356 044 sequence 6e-05 | 32560:32424 | 32106:33242 |
| NZ_JABWGN010000007.1 | Nonomuraea montanisoli SMC 257 NODE_7_length_551020_cov_15.5127 1e-34 | 245158:245022 | 244944:244549 |
| NZ_JACHIU010000001.1 | Sphaerisporangium rubeum DSM 44936 Ga0436920_01 3e-09 | 905163:905019 | 904693:906338 |
| NZ_JABBWX010000002.1 | Microbispora sp. H10830 NODE_2 9e-09 | 519682:519535 | 519434:520292 |
| NZ_BOOQ01000010.1 | Planotetraspora silvatica NBRC 100141 010 sequence 4e-34 | 56314:56166 | 56072:56941 |
| NZ_BONV01000004.1 | Planotetraspora kaengkrachanensis NBRC 104272 04 sequence 4e-07 | 86115:85965 | 85866:86733 |
| NZ_BBYK01000081.1 | Microtetraspora fusca NBRC 13915 2e-11 | 21803:21948 | 21189:22019 |
| NZ_JOAG01000032.1 | Nonomuraea candida NRRL B-24552 contig30.1 4e-34 | 116405:116516 | 115316:116791 |
| NZ_JACHMI010000001.1 | Nonomuraea rubra DSM 43768 Ga0374023_01 4e-34 | 12105791:12105902 | 12106713:12105946 |
| NZ_JACHJB010000001.1 | Nonomuraea muscovyensis DSM 45913 Ga0436939_01 4e-34 | 3205671:3205534 | 3205498:3206433 |
| NZ_BOOM01000056.1 | Planomonospora parontospora subsp. antibiotica NBRC 15869 056 sequence 2e-18 | 48556:48410 | 48243:49153 |
| NZ_JABCPZ010000379.1 | Nonomuraea sp. NN258 NODE_379_length_5909_cov_16.307679 4e-34 | 1106:1217 | 1698:1258 |
| NZ_JACHGN010000019.1 | Thermocatellispora tengchongensis DSM 45615 Ga0415383_19 4e-34 | 89177:89040 | 88449:90050 |
| NZ_JAHKRM010000022.1 | Nonomuraea guangzhouensis CGMCC 4.7101 NODE_22 1e-06 | 18112:18258 | 17337:18291 |
| NZ_BMNH01000008.1 | Nonomuraea cavernae CGMCC 4.7368 08 sequence 1e-33 | 38112:37970 | 37959:38781 |
| NZ_JAHKRL010000466.1 | Nonomuraea rhizosphaerae CGMCC 4.7431 NODE_2118 1e-33 | 23520:23630 | 22845:23671 |
| NZ_BMPY01000012.1 | Microbispora rosea subsp. aerata JCM 3076 012 sequence 1e-06 | 61628:61480 | 61068:62232 |
| NZ_VRLV01000005.1 | Nonomuraea typhae p1410 Scaffold5 1e-33 | 370777:370638 | 370614:371310 |
| NZ_VIRM01000001.1 | Microbispora hainanensis DSM 45428 1 4e-07 | 193539:193397 | 193156:194142 |
| NZ_SMKO01000012.1 | Nonomuraea deserti KC310 NODE_12_length_114270_cov_59.3796 1e-33 | 10638:10748 | 9932:10808 |
| NZ_SMJZ01000104.1 | Nonomuraea longispora KC201 NODE_104_length_29718_cov_20.4514 1e-33 | 17012:16902 | 16841:17712 |
| NZ_JADBEF010000001.1 | Nonomuraea africana DSM 43748 Ga0415182_01 3e-08 | 7875966:7875833 | 7875830:7876425 |
| NZ_VJVX01000010.1 | Microbispora sp. CSR-4 BHAGLCML_10 1e-06 | 30611:30453 | 30148:31211 |
| NZ_VDMA02000004.1 | Microbispora catharanthi CR1-09 NODE_4_length_486820_cov_45.204102 3e-09 | 367737:367590 | 367489:368343 |
| NZ_QHHZ01000017.1 | Nonomuraea sp. WAC 01424 AA000381-17_WAC01424 5e-33 | 188479:188624 | 187857:188631 |
| NZ_KI866523.1 | Microbispora sp. ATCC PTA-5024 scaffold00002 6e-11 | 1267485:1267339 | 1267304:1268042 |
| NZ_JADOGI010000261.1 | Nonomuraea cypriaca K274 K274_contig_261 5e-33 | 5637:5748 | 5389:5916 |
| NZ_VCKY01000086.1 | Nonomuraea turkmeniaca DSM 43926 NODE_86_length_39689_cov_15.4278 5e-33 | 4058:4169 | 3409:4209 |
| NZ_SAUN01000001.1 | Nonomuraea polychroma DSM 43925 Ga0197504_11 5e-33 | 9905380:9905269 | 9905229:9906021 |
| NZ_JABBWV010000007.1 | Microbispora sp. H11081 NODE_7 0.0002 | 249980:249833 | 249765:250571 |
| NZ_JAHKRO010000001.1 | Nonomuraea ceibae KCTC 39826 NODE_1 3e-09 | 321775:321644 | 321636:322689 |
| NZ_FNDJ01000027.1 | Nonomuraea jiangxiensis CGMCC 4.6533 2e-32 | 53938:53828 | 53786:52938 |
| NZ_JACDUR010000003.1 | Nonomuraea soli DSM 45533 Ga0415313_03 0.0002 | 137582:137721 | 136983:137771 |
| NZ_BBZG01000002.1 | Nonomuraea pusilla NBRC 110462 2e-32 | 547713:547603 | 547487:548280 |
| NZ_BOOU01000090.1 | Sphaerisporangium rufum NBRC 109079 090 sequence 5e-06 | 17882:17737 | 16849:18538 |
| NZ_PVNG01000008.1 | Nonomuraea fuscirosea CGMCC 4.7104 Ga0171605_108 6e-32 | 19667:19557 | 19471:18692 |
| NZ_JACHMB010000001.1 | Nonomuraea jabiensis DSM 45507 Ga0374001_01 6e-32 | 3426753:3426642 | 3426567:3425797 |
| NZ_JAHKRN010000011.1 | Nonomuraea harbinensis CGMCC 4.7106 NODE_11 2e-31 | 19857:19746 | 19715:20426 |
| NZ_BOOP01000011.1 | Planotetraspora phitsanulokensis NBRC 104273 11 sequence 2e-31 | 214358:214496 | 213742:214565 |
| NZ_BOOL01000046.1 | Planomonospora parontospora subsp. parontospora NBRC 13880 046 sequence 2e-18 | 101288:101434 | 100653:101593 |
| NZ_SMKQ01000032.1 | Nonomuraea terrae CH32 NODE_32_length_56269_cov_16.4615 2e-31 | 53875:53737 | 53041:55159 |
| NZ_VCJS01000006.1 | Nonomuraea sp. 160415 NODE_6_length_145743_cov_23.9232 2e-31 | 436:547 | 0:586 |
| NZ_RRYT01000022.1 | Thermomonospora catenispora 3-22-3 Scaffold22 2e-31 | 19811:19676 | 19312:21014 |
| NZ_VFPQ01000001.1 | Thermopolyspora flexuosa DSM 43186 Ga0197564_11 6e-11 | 4816301:4816475 | 4815909:4817163 |
| NZ_JAATEP010000010.1 | Nonomuraea sp. FMUSA5-5 10 2e-31 | 112664:112777 | 113650:112823 |
| NZ_BOOA01000035.1 | Acrocarpospora phusangensis NBRC 108782 035 sequence 8e-10 | 19639:19495 | 19077:20502 |
| NZ_JADBEK010000001.1 | Nonomuraea angiospora DSM 43173 Ga0415180_01 7e-31 | 8211244:8211152 | 8211058:8210276 |
| NZ_KZ559469.1 | Nonomuraea indica DRQ-2 Scaffold4 2e-30 | 596199:596336 | 595575:596396 |
| NZ_POUD01000441.1 | Nonomuraea sp. KC333 NODE_441_length_4685_cov_36.5461 2e-30 | 2172:2283 | 1059:3447 |
| NZ_BLAE01000009.1 | Acrocarpospora macrocephala NBRC 16266 009 sequence 2e-11 | 210887:211031 | 209905:211637 |
| NZ_JACXRZ010000005.1 | Microbispora camponoti 2C-HV3 Scaffold4_1 1e-06 | 279272:279422 | 278661:279607 |
| NZ_SMLD01000160.1 | Nonomuraea mesophila 6K102 NODE_160_length_21776_cov_39.2184 3e-29 | 21379:21269 | 21180:21776 |
| NZ_FNFB01000024.1 | Nonomuraea maritima CGMCC 4.5681 4e-07 | 89084:89216 | 88491:90165 |
| NZ_BBYJ01000017.1 | Microtetraspora malaysiensis NBRC 100735 2e-11 | 346694:346550 | 346436:347313 |
| NZ_KZ084324.1 | Thermoactinospora rubra YIM 77501 Scaffold1 1e-28 | 374537:374444 | 374337:375164 |
| NZ_CP068985.1 | Nonomuraea coxensis DSM 45129 1e-28 | 350959:350848 | 350752:350966 |
| NZ_BLAF01000052.1 | Acrocarpospora pleiomorpha NBRC 16267 052 sequence 2e-11 | 21806:21950 | 21102:22708 |
| NZ_BBXD01000008.1 | Herbidospora mongoliensis NBRC 105882 6e-18 | 574036:573890 | 573871:574594 |
| NZ_BCBX01000032.1 | Microbispora sp. GMKU363 GMKU 363 5e-06 | 134331:134191 | 134134:135132 |
| NZ_JAHCST010000003.1 | Acrocarpospora sp. H8750 NODE_3 6e-11 | 326:182 | 0:0 |
| NZ_WXEW01000003.1 | Herbidospora solisilvae NEAU-GS84 Scaffold3 8e-16 | 519497:519352 | 519333:520044 |
| NZ_BBXG01000028.1 | Herbidospora cretacea NBRC 15474 3e-15 | 13813:13668 | 13648:14373 |
| NZ_JABTEX010000001.1 | Planomonospora sp. ID82291 NODE_1_length_2453277_cov_626.432137 1e-13 | 1653601:1653746 | 1652926:1653919 |
| NZ_BBXE01000045.1 | Herbidospora yilanensis NBRC 106371 2e-17 | 1008:862 | 843:1552 |
| NZ_BBXC01000017.1 | Herbidospora sakaeratensis NBRC 102641 2e-18 | 68797:68651 | 68632:69345 |
| NZ_BOOG01000021.1 | Sphaerimonospora thailandensis NBRC 107569 021 sequence 2e-25 | 38549:38687 | 37710:38698 |
| NZ_BBXF01000002.1 | Herbidospora daliensis NBRC 106372 2e-16 | 997705:997560 | 997541:998253 |
| NZ_SZQA01000005.1 | Herbidospora galbida NEAU-GS14 Scaffold5 8e-16 | 163470:163616 | 162921:163635 |
| NZ_QLYX01000005.1 | Actinomadura craniellae LHW63021 contig5 3e-23 | 384477:384333 | 384328:385106 |
| NZ_JACHJU010000001.1 | Streptosporangium album DSM 43023 Ga0436960_01 2e-10 | 3299416:3299269 | 3299083:3300052 |
| NZ_JOEQ01000043.1 | Streptosporangium amethystogenes NRRL B-2639 contig43.1 2e-18 | 44720:44574 | 44492:45466 |
| NZ_JACHMP010000001.1 | Streptosporangium becharense DSM 46887 Ga0374018_01 2e-11 | 3515831:3515977 | 3515274:3516169 |
| NZ_PVZV01000001.1 | Actinocorallia populi A251 contig1 6e-19 | 845583:845475 | 845434:846082 |
| NZ_FRCS01000002.1 | Cryptosporangium aurantiacum DSM 46144 2e-18 | 664775:664912 | 664645:665366 |
| NZ_FOQY01000004.1 | Streptosporangium canum CGMCC 4.2126 2e-12 | 441242:441096 | 440814:442041 |
| NZ_NGFP01000006.1 | Streptosporangium minutum M26 scaffold6.1 6e-11 | 17515:17661 | 16869:17960 |
| NZ_JACHJP010000001.1 | Streptosporangium saharense CECT 8840 Ga0436955_01 5e-06 | 172756:172602 | 172571:171828 |
| NZ_JOEP01000051.1 | Streptosporangium roseum NRRL B-2638 contig51.1 6e-11 | 34122:33976 | 33731:35081 |
| NZ_BMPI01000022.1 | Dactylosporangium sucinum JCM 19831 022 sequence 5e-25 | 23393:23241 | 23146:23481 |
| NZ_BNBB01000010.1 | Streptosporangium violaceochromogenes JCM 3281 010 sequence 1e-14 | 21181:21332 | 20497:21546 |
| NZ_BOON01000072.1 | Planosporangium mesophilum NBRC 109066 072 sequence 1e-33 | 30024:30228 | 29904:30394 |
| NZ_BONC01000027.1 | Asanoa iriomotensis NBRC 100142 027 sequence 5e-14 | 99180:99071 | 99068:99287 |
| NZ_KK073874.1 | Cryptosporangium arvum DSM 44712 CryarDRAFT_CAH.1 3e-09 | 326443:326309 | 326168:326548 |
| NZ_VIRS01000011.1 | Cryptosporangium phraense A-T 5661 11 6e-13 | 176547:176406 | 175957:176639 |
| NZ_BONE01000027.1 | Asanoa siamensis NBRC 107932 027 sequence 7e-12 | 13413:13298 | 13295:13525 |
| NZ_BLPF01000001.1 | Phytohabitans houttuyneae NBRC 108639 1 sequence 1e-52 | 3650949:3651139 | 3650899:3651159 |
| NZ_FNQB01000001.1 | Asanoa ishikariensis DSM 44718 2e-11 | 738028:737914 | 737911:738200 |
| NZ_BONZ01000006.1 | Rugosimonospora africana NBRC 104875 006 sequence 7e-36 | 255688:255873 | 255626:255952 |
| NZ_RJKE01000001.1 | Actinocorallia herbida DSM 44254 Ga0197506_11 8e-11 | 9364105:9363987 | 9363920:9364585 |
| NZ_FNPH01000011.1 | Micromonospora pattaloongensis DSM 45245 0 | 42201:42561 | 42200:42562 |
| NZ_BLPG01000001.1 | Phytohabitans rumicis NBRC 108638 1 sequence 3e-53 | 3976335:3976527 | 3976285:3976539 |
| NZ_BBQH01000015.1 | Micromonospora sp. NBRC 107566 2e-37 | 127855:127693 | 127619:127959 |
| NZ_KB913029.1 | Sporichthya polymorpha DSM 43042 SpopoDRAFT_scaffold1.1 4e-08 | 1285567:1285708 | 1285354:1285776 |
| NZ_JADMLH010000005.1 | Pseudosporangium sp. NEAU-24 Scaffold5 2e-29 | 316496:316660 | 316378:316737 |
| NZ_JACCCC010000001.1 | Spinactinospora alkalitolerans CXB654 Ga0415162_01 4e-08 | 24564:24671 | 24046:24756 |
| NZ_BMQJ01000012.1 | Streptosporangium pseudovulgare JCM 3115 12 sequence 4e-13 | 81213:81355 | 80461:81617 |
| NZ_BCRK01000050.1 | Nocardiopsis trehalosi NBRC 14201 5e-07 | 9331:9218 | 9031:10408 |
| NZ_QUMQ01000001.1 | Asanoa ferruginea DSM 44099 Ga0197490_11 5e-07 | 1724897:1724939 | 1724767:1725028 |
| NZ_KB903835.1 | Longispora albida DSM 44784 A3G1DRAFT_scaffold_5.6 2e-12 | 385842:385720 | 385674:386001 |
| NZ_BOMK01000039.1 | Actinoplanes digitatis NBRC 12512 039 sequence 7e-42 | 52754:52943 | 52668:52950 |
| NZ_JACHDB010000001.1 | Nocardiopsis composta DSM 44551 Ga0415200_01 2e-05 | 4371122:4371280 | 4370697:4371841 |
| NZ_ANBC01000894.1 | Nocardiopsis lucentensis DSM 44048 contig_894 2e-05 | 907:971 | 207:1123 |
| NZ_JACHJT010000001.1 | Lipingzhangella halophila DSM 102030 Ga0436959_01 2e-05 | 5916508:5916352 | 5916221:5917131 |
| NZ_LMSS01000002.1 | Phycicoccus sp. Soil802 contig_2 1e-07 | 561526:561631 | 561402:562122 |
| NZ_LMCM01000010.1 | Phycicoccus sp. Root101 contig_7 2e-06 | 354170:354297 | 353982:354682 |
| NZ_SNXZ01000001.1 | Labedaea rhizosphaerae DSM 45361 Ga0310470_101 3e-06 | 792481:792387 | 792145:792838 |
| NZ_CP074132.1 | Nocardiopsis sp. HDS12 0.001 | 15176:15100 | 14832:15875 |
| NZ_LEKI01000007.1 | Nocardiopsis sp. RV163 contig7_size78222 0.001 | 23427:23351 | 23085:24122 |
| NZ_RCZM01000005.1 | Pedococcus bigeumensis S9.3A scaffold5.1 2e-05 | 267590:267443 | 267027:267700 |
| NZ_QEIO01000039.1 | Marinitenerispora sediminis TPS16 scaffold_38 0.003 | 13402:13560 | 14468:13659 |
| NZ_LMER01000020.1 | Angustibacter sp. Root456 contig_9 0.003 | 143346:143437 | 142908:143504 |
| NZ_BONI01000077.1 | Catellatospora coxensis NBRC 107359 077 sequence 1e-07 | 7173:7015 | 6865:7309 |
| NZ_VOHR01000509.1 | Segeticoccus rhizosphaerae YJ01 509 8e-11 | 624:732 | 511:1144 |
| NZ_JRUV01000278.1 | Thermobifida halotolerans DSM 44931 contig278 0.012 | 976:1074 | 0:1257 |
| NZ_VFOQ01000002.1 | Oryzihumus leptocrescens DSM 18082 Ga0197546_12 0.0002 | 438295:438418 | 438197:438833 |
| NZ_JACHMN010000002.1 | Allocatelliglobosispora scoriae DSM 45362 Ga0373994_02 2e-24 | 3897830:3897678 | 3897563:3897952 |
| NZ_VTZW01000002.1 | Streptomonospora sp. PA3 scaffold14.1 0.012 | 11913:11795 | 11620:12734 |
| NZ_BOMS01000051.1 | Actinoplanes palleronii NBRC 14916 051 sequence 3e-15 | 93083:93014 | 92838:93342 |
| NZ_QVAI01000005.1 | Jiangella endophytica KE2-3 5 0.012 | 3259:3352 | 3119:3633 |
| NZ_MSIE01000036.1 | Actinophytocola xanthii 11-183 contig36 9e-06 | 27808:27939 | 27551:28166 |
| NZ_SHKY01000001.1 | Krasilnikovia cinnamomea DSM 45162 Ga0197533_11 4e-32 | 3676716:3676528 | 3676412:3676785 |
| NZ_CABEGA010000065.1 | Cellulomonas hominis IHUMI-CSURP8420 3e-08 | 3206:3089 | 3015:3302 |
| NZ_CH672413.1 | Janibacter sp. HTCC2649 scf_1099316001559 0.01 | 2009680:2009570 | 2009210:2009778 |
| NZ_VWVS01000024.1 | Nocardiopsis deserti H13 contig024 0.04 | 9596:9520 | 9293:10292 |
| NZ_AVPL01000058.1 | Knoellia aerolata DSM 18566 contig69 2e-06 | 2353:2246 | 1896:2440 |
| NZ_JZKF01000005.1 | Actinoplanes rectilineatus NRRL B-16090 contig-5 2e-29 | 91459:91291 | 91271:91576 |
| NZ_JACHMY010000001.1 | Kribbella italica DSM 28967 Ga0415174_01 0.04 | 7010082:7010017 | 7009997:7010279 |
| NZ_WOTN01000004.1 | Fodinicola acaciae GKU 173 4 4e-07 | 114569:114719 | 114456:114734 |
| NZ_LR134501.1 | Nocardiopsis dassonvillei NCTC10488 1 0.04 | 4300425:4300501 | 4299702:4300776 |
| NZ_LOJP01000001.1 | Actinoplanes sp. TFC3 Actinoplanes_sp._TFC3_1 0.04 | 372202:372159 | 372072:372315 |
| NZ_BNBD01000012.1 | Streptomyces mashuensis JCM 4059 12 sequence 0.04 | 71441:71404 | 71305:71613 |
| NZ_FUWS01000013.1 | Marinactinospora thermotolerans DSM 45154 0.04 | 51575:51464 | 51286:52030 |
| NZ_JACCAB010000001.1 | Pedococcus badiiscoriae DSM 23987 Ga0104601_01 0.04 | 1123687:1123598 | 1123212:1123816 |
| NZ_JAAGLV010000016.1 | Streptomyces sp. SID13031 NODE_16_length_219598_cov_96.241_ID_310 0.04 | 144778:144843 | 144571:144867 |
| NZ_QPNC01000031.1 | Nocardiopsis sp. FIRDI 009 contig_31 0.04 | 99602:99665 | 98891:99829 |
| NZ_CP070961.1 | Nocardioides sp. zg-1228 0.04 | 336275:336232 | 336183:336441 |
| NZ_RQIK01000001.1 | Cellulosimicrobium cellulans ATCC 21606 Scaffold1_1 8e-171 | 149046:148634 | 148633:149047 |
| NZ_CAOI01000235.1 | Cellulosimicrobium cellulans LMG 16121 6e-170 | 1777:1365 | 1364:1778 |
| NZ_CP053419.1 | Cellulosimicrobium sp. 72-3 5e-169 | 762386:762798 | 762385:762799 |
| NZ_VEBQ01000001.1 | Cellulosimicrobium sp. TH-20 DE0282 NODE_1_length_969071_cov_35.900513 5e-165 | 661669:662082 | 661668:662083 |
| NZ_CP017660.1 | Cellulosimicrobium sp. JZ28 6e-162 | 300554:300968 | 300553:300969 |
| NZ_SOZH01000008.1 | Cellulosimicrobium funkei JCM 14302 contig8 2e-161 | 296018:295605 | 295604:296019 |
| NZ_JAACJC010000003.1 | Cellulosimicrobium sp. SL-1 NODE_3 2e-161 | 163086:162673 | 162672:163087 |
| NZ_NEDO01000001.1 | Cellulosimicrobium sp. KWT-B NODE001_cov_66.1082 2e-161 | 707373:706960 | 706959:707374 |
| NZ_JABAGH010000004.1 | Cellulosimicrobium aquatile WB02_D5_03 Contig_4 1e-160 | 279381:279794 | 279380:279795 |
| NZ_FTMI01000001.1 | Cellulosimicrobium aquatile 3bp 1e-159 | 318921:318508 | 318507:318922 |
| NZ_CP072387.1 | Cellulosimicrobium cellulans ORNL-0100 1e-159 | 2791366:2790953 | 2790952:2791367 |
| NZ_VEIN01000005.1 | Cellulosimicrobium sp. TH-20 DE0020 NODE_5_length_394865_cov_11.322454 1e-159 | 155677:155264 | 155232:155678 |
| NZ_JAAMSN010000214.1 | Cellulosimicrobium cellulans 1B1-1 contig00214 5e-157 | 1912:1500 | 1499:1913 |
| NZ_VEDT01000001.1 | Cellulosimicrobium sp. TH-20 DE0194 NODE_1_length_619924_cov_24.826351 2e-153 | 295049:295462 | 295048:295463 |
| NZ_LUAZ01000052.1 | Cellulosimicrobium sp. I38E scaffold51.1 6e-65 | 2191:1885 | 1847:2221 |
| NZ_JAFGYF010000001.1 | Cellulosimicrobium cellulans MP1 contig1 1e-63 | 1006526:1006838 | 1006496:1006892 |
| NZ_BJNZ01000010.1 | Cellulosimicrobium cellulans NBRC 15516 10 sequence 1e-62 | 109181:108875 | 108838:109211 |
| NZ_JNBQ01000002.1 | Cellulosimicrobium funkei U11 contig00002 2e-60 | 136166:136471 | 136136:136508 |
| NZ_WMKA01000026.1 | Cellulosimicrobium composti BIT-GX5 Scaffold26 2e-53 | 23156:23495 | 23137:23521 |
| NZ_LWGL01000159.1 | Cellulosimicrobium cellulans JZ5 J5_S3.NODE_159 2e-33 | 1736:1508 | 1464:1749 |
| NZ_PDJJ01000001.1 | Isoptericola jiangsuensis DSM 21863 Ga0074734_11 8e-23 | 569833:570077 | 569784:570107 |
| NZ_CP045529.1 | Luteimicrobium xylanilyticum HY-24 5e-10 | 559484:559705 | 559481:559803 |
| NZ_LRIE01000067.1 | Oerskovia enterophila VJag OJAG_contig000067 9e-08 | 12008:12146 | 11956:12371 |
| NZ_JACSQE010000017.1 | Oerskovia sp. Sa2CUA8 NODE_17_length_86765_cov_18.965924 2e-07 | 53601:53738 | 53550:53950 |
| NZ_JAFBBV010000001.1 | Oerskovia paurometabola DSM 14281 Ga0451129_01 2e-06 | 825174:825311 | 825123:825523 |
| NZ_LMIL01000008.1 | Oerskovia sp. Root22 contig_3 2e-07 | 59736:59839 | 59610:60077 |
| NZ_LMJG01000012.1 | Oerskovia sp. Root918 contig_3 0.0008 | 53012:53110 | 52786:53240 |
| NZ_SDJR01000007.1 | Oerskovia turbata JCM3160 contig7 2e-06 | 40333:40436 | 40295:40701 |
| NZ_JAFBBO010000001.1 | Oerskovia jenensis DSM 46000 Ga0451159_01 2e-06 | 1831041:1830940 | 1830688:1831176 |
| NZ_JACSQF010000002.1 | Oerskovia sp. Sa2CUA9 NODE_2_length_366962_cov_19.007956 5e-06 | 49322:49460 | 49270:49699 |
| NZ_KB894408.1 | Actinokineospora enzanensis DSM 44649 C503DRAFT_scaffold00006.6 4e-05 | 98121:97929 | 0:0 |
| NZ_LN651328.1 | Mobilicoccus massiliensis SIT2 4e-05 | 221348:221493 | 220754:221742 |
| NZ_VFOA01000001.1 | Cellulomonas sp. SLBN-39 Ga0314631_11 0.006 | 3144870:3145035 | 3144486:3145126 |
| NZ_CP041244.1 | Actinomadura sp. WMMA1423 0.018 | 8504091:8504208 | 8503981:8504211 |
| NZ_JACSPN010000031.1 | Oerskovia sp. Sa1BUA8 NODE_31_length_44439_cov_31.347753 0.018 | 38312:38430 | 38197:38511 |
| NZ_AXCY01000025.1 | Cellulomonas carbonis T26 contig37 0.0003 | 35993:35773 | 35515:36184 |
| NZ_CP081862.1 | Cellulomonas sp. C5510 0.006 | 345059:345178 | 344918:345225 |
| NZ_CAACUY010000002.1 | Actinomadura fibrosa isolate LMG 29177 2e-17 | 134959:134813 | 134811:134191 |
| NZ_WEGH01000002.1 | Actinomadura macrotermitis RB68 ARB68_2 2e-18 | 1239563:1239419 | 1239415:1238795 |
| NZ_SGXJ01000004.1 | Kribbella sp. VKM Ac-2569 Ga0310567_104 8e-05 | 769890:769801 | 769782:770066 |
| NZ_AQUZ01000077.1 | Kribbella catacumbae DSM 19601 A3ESDRAFT_scaffold_76.77_C 8e-05 | 21123:21191 | 20914:21197 |
| NZ_VFMM01000001.1 | Kribbella jejuensis DSM 17305 Ga0197573_11 0.039 | 3684085:3684018 | 3684001:3684278 |
| NZ_SNWS01000008.1 | Kribbella sp. VKM Ac-2571 Ga0310569_108 0.039 | 59575:59664 | 59399:59671 |
| NZ_JACBZI010000001.1 | Nocardioides marinus DSM 18248 Ga0104575_01 0.039 | 49876:49827 | 49788:50144 |
| NZ_LNTD01000076.1 | Cellulomonas sp. B6 contig_167 0.039 | 20213:20357 | 20092:20417 |
| NZ_JABEPQ010000003.1 | Knoellia sp. DB2414S contig3 1e-07 | 91513:91396 | 91030:91605 |
| NZ_SAYU02000026.1 | Phycicoccus flavus CMS6Z-2 Scaffold26 2e-05 | 20697:20803 | 20590:21242 |
| NZ_BMEA01000001.1 | Knoellia flava CGMCC 1.10749 1 sequence 7e-05 | 1472305:1472198 | 1471853:1472393 |
| NZ_LMSC01000006.1 | Phycicoccus sp. Soil748 contig_3 7e-05 | 416874:417007 | 416698:417433 |
| NZ_AQXW01000004.1 | Demetria terragena DSM 11295 F562DRAFT_scaffold00001.1_C 6e-22 | 1596695:1596602 | 1596217:1596808 |
| NZ_VFOK01000002.1 | Barrientosiimonas humi DSM 24617 Ga0264280_12 0.003 | 255454:255548 | 255319:256109 |
| NZ_QLVD01000023.1 | Ornithinimicrobium murale DSM 22056 s22056_Contig2 2e-08 | 412333:412249 | 411805:412432 |
| NZ_AVPJ01000008.1 | Knoellia sinensis KCTC 19936 contig3 0.01 | 73368:73261 | 72887:73456 |
| NZ_AVPK01000003.1 | Knoellia subterranea KCTC 19937 contig4 0.01 | 100135:100242 | 100049:100595 |
| NZ_HF570958.1 | Tetrasphaera japonica T1-X7 0.01 | 3440102:3440061 | 3439453:3440226 |
| NC_014830.1 | Intrasporangium calvum DSM 43043 0.01 | 559523:559610 | 559366:559929 |
| NZ_CP011112.1 | Luteipulveratus mongoliensis MN07-A0370 0.037 | 4903192:4903102 | 4902496:4903393 |
| NZ_QHHV01000056.1 | Actinoplanes sp. ATCC 53533 AA000006-56_GDW00994 5e-50 | 4299:4081 | 4080:4378 |
| NZ_AP022871.1 | Phytohabitans suffuscus NBRC 105367 3e-46 | 4056349:4056159 | 4056138:4055653 |
| NZ_BOQL01000071.1 | Actinoplanes auranticolor NBRC 12245 071 sequence 1e-45 | 64912:65100 | 64830:65112 |
| NZ_BMMX01000014.1 | Mangrovihabitans endophyticus CGMCC 4.7299 014 sequence 2e-42 | 31818:31991 | 31690:32117 |
| NZ_BOMQ01000063.1 | Actinoplanes nipponensis NBRC 14063 063 sequence 3e-40 | 159677:159898 | 159600:159935 |
| NZ_BOMF01000089.1 | Actinoplanes capillaceus NBRC 16408 089 sequence 7e-36 | 48669:48470 | 48450:48940 |
| NZ_BOME01000063.1 | Actinoplanes campanulatus NBRC 12511 063 sequence 8e-35 | 29918:30117 | 29633:30137 |
| NZ_JAGFNS010000017.1 | Actinoplanes sp. NEAU-H7 Scaffold17 8e-35 | 82052:82251 | 81876:82270 |
| NZ_JAIWNC010000011.1 | Wangella sp. NEAU-J3 Scaffold10_1 3e-34 | 197154:197375 | 197095:197395 |
| NZ_JAHXZI010000016.1 | Actinoplanes hulinensis NEAU-M9 Scaffold16 4e-33 | 152165:152364 | 151981:152384 |
| NZ_BOMP01000038.1 | Actinoplanes lobatus NBRC 12513 038 sequence 4e-33 | 92060:91861 | 91840:92261 |
| NZ_PVZG01000006.1 | Pseudosporangium ferrugineum DSM 45348 Ga0181035_106 2e-31 | 173733:173531 | 173452:173844 |
| NZ_CP061913.1 | Dactylosporangium vinaceum NRRL B-16297 2e-31 | 229002:229157 | 228926:229204 |
| NZ_BOMO01000150.1 | Actinoplanes italicus NBRC 13911 150 sequence 2e-30 | 28418:28615 | 28197:28629 |
| NZ_BONA01000066.1 | Actinoplanes xinjiangensis NBRC 106528 066 sequence 6e-30 | 93838:93640 | 93512:94165 |
| NZ_FONV01000014.1 | Actinoplanes philippinensis DSM 43019 8e-29 | 132229:132030 | 132024:132439 |
| NZ_BOMG01000084.1 | Actinoplanes couchii NBRC 106145 084 sequence 9e-28 | 61260:61460 | 61017:61488 |
| NZ_BOMY01000018.1 | Actinoplanes tereljensis NBRC 105297 18 sequence 9e-28 | 61543:61706 | 61380:61710 |
| NZ_BOOY01000022.1 | Spirilliplanes yamanashiensis NBRC 15828 22 sequence 3e-27 | 122336:122146 | 122110:122406 |
| NZ_JOJL01000028.1 | Actinoplanes subtropicus NRRL B-24665 contig28.1 4e-26 | 180310:180472 | 179889:180607 |
| NZ_BOQN01000029.1 | Actinoplanes toevensis NBRC 105298 029 sequence 4e-26 | 20377:20533 | 20212:20537 |
| NZ_BOMM01000051.1 | Actinoplanes ferrugineus NBRC 15555 051 sequence 4e-26 | 111713:111869 | 111525:111873 |
| NZ_RJKL01000001.1 | Couchioplanes caeruleus DSM 43634 Ga0197494_11 4e-26 | 3955871:3956031 | 3955747:3956032 |
| NZ_BOQP01000052.1 | Actinoplanes consettensis NBRC 14913 052 sequence 1e-25 | 123381:123581 | 123321:123673 |
| NZ_BOMN01000031.1 | Actinoplanes humidus NBRC 14915 031 sequence 1e-25 | 93811:93591 | 93538:93851 |
| NZ_BOMZ01000069.1 | Actinoplanes utahensis NBRC 13244 069 sequence 5e-25 | 65806:65654 | 65614:65903 |
| NZ_JADQTO010000014.1 | Actinoplanes sp. NEAU-A11 Scaffold14 5e-25 | 125148:125349 | 124943:125368 |
| NZ_QLMJ01000014.1 | Actinoplanes lutulentus CGMCC 4.7090 Ga0171608_114 2e-24 | 131845:131645 | 131626:131907 |
| NZ_KB903320.1 | Actinoplanes globisporus DSM 43857 A3CQDRAFT_scaffold_32.33 6e-24 | 101289:101446 | 100998:101565 |
| NZ_JABBNC010000019.1 | Actinoplanes sp. TBRC 11911 19 3e-22 | 163262:163096 | 162893:163400 |
| NZ_BOML01000038.1 | Actinoplanes durhamensis NBRC 14914 038 sequence 9e-22 | 231225:231069 | 230957:231670 |
| NZ_BMQB01000005.1 | Pilimelia anulata JCM 3090 05 sequence 3e-21 | 244570:244408 | 244408:244887 |
| NZ_BOPH01000018.1 | Virgisporangium ochraceum NBRC 16418 018 sequence 1e-20 | 83307:83160 | 82958:83343 |
| NZ_BOMV01000060.1 | Actinoplanes rishiriensis NBRC 108556 060 sequence 5e-19 | 159914:159755 | 159575:160546 |
| NZ_AP023356.1 | Actinoplanes ianthinogenes NBRC 13996 5e-19 | 5006351:5006269 | 5005913:5007244 |
| NZ_JAHKKG010000009.1 | Actinoplanes bogorensis NBRC 110975 NODE_9_length_454185_cov_36.317946 1e-06 | 337074:337149 | 336627:337498 |
| NZ_BOMD01000068.1 | Actinoplanes brasiliensis NBRC 13938 068 sequence 6e-05 | 7925:7853 | 7822:8222 |
| NZ_BOMH01000048.1 | Actinoplanes cyaneus NBRC 14990 048 sequence 4e-07 | 109207:109286 | 109812:109333 |
| NZ_BMQC01000003.1 | Pilimelia terevasa JCM 3091 03 sequence 2e-17 | 135133:135290 | 135026:135357 |
| NZ_JAHWFK010000034.1 | Actinoplanes sp. TRM66264-DLM contig34 0.003 | 171320:171392 | 171039:171396 |
| NZ_BOMI01000130.1 | Actinoplanes deccanensis NBRC 13994 130 sequence 2e-05 | 250:175 | 0:0 |
| NZ_BOMW01000026.1 | Actinoplanes siamensis NBRC 109076 026 sequence 2e-16 | 51114:51012 | 50699:51516 |
| NZ_AP024745.1 | Actinoplanes sp. L3-i22 5e-06 | 290168:290088 | 290066:289587 |
| NZ_JAENHO010000016.1 | Actinoplanes lichenicola LDG1-01 NODE_16_length_210066_cov_35.124050 0.0007 | 146358:146278 | 146264:146765 |
| NZ_JAENHP010000006.1 | Actinoplanes ovalisporus LDG1-06 NODE_6_length_459480_cov_30.693412 2e-16 | 168191:168129 | 167877:168493 |
| NZ_AWOO02000007.1 | Actinomadura madurae LIID-AJ290 contig00007 8e-16 | 167833:167663 | 167657:168571 |
| NZ_SMJW01000002.1 | Actinomadura bangladeshensis DSM 45347 NODE_2_length_108484_cov_35.3617 3e-15 | 550:695 | 0:717 |
| NZ_FZNR01000011.1 | Actinoplanes regularis DSM 43151 1e-07 | 128299:128218 | 128190:128660 |
| NZ_BOPG01000070.1 | Virgisporangium aurantiacum NBRC 16421 070 sequence 1e-14 | 7580:7432 | 7420:7676 |
| NZ_BOPF01000014.1 | Virgisporangium aliadipatigenens NBRC 105644 014 sequence 1e-14 | 250893:250753 | 250720:250934 |
| NZ_JNXY01000005.1 | Catenuloplanes japonicus NRRL B-16061 contig5.1 4e-14 | 149426:149568 | 149326:149653 |
| NZ_BOMB01000025.1 | Actinocatenispora rupis NBRC 107355 25 sequence 4e-14 | 111182:111050 | 110526:111457 |
| NZ_JACIBV010000001.1 | Nonomuraea dietziae DSM 44320 Ga0374022_01 4e-14 | 5263847:5263994 | 5263404:5263996 |
| NZ_RJAC01000002.1 | Actinoplanes sp. LAM7112 C_Contig2 1e-13 | 748433:748495 | 748123:748644 |
| NZ_ML769318.1 | Catellatospora paridis NEAU-CL2 Scaffold9 2e-12 | 112516:112356 | 112353:112652 |
| NZ_BLAD01000085.1 | Acrocarpospora corrugata NBRC 13972 085 sequence 2e-11 | 60841:60697 | 60648:61472 |
| NZ_JAHRCY010000007.1 | Catellatospora sp. NEAU-YM18 Scaffold7 6e-11 | 98769:98926 | 98615:99234 |
| NZ_BNEM01000002.1 | Catellatospora sp. TT07R-123 2 sequence 6e-11 | 4292894:4293051 | 4292741:4293515 |
| NZ_QJUG01000228.1 | Allorhizocola rhizosphaerae CPCC 204380 contig228 2e-10 | 4670:4523 | 4142:5186 |
| NZ_VIQO01000013.1 | Catellatospora sichuanensis H14505 Scaffold9_1 3e-09 | 91994:92151 | 91858:92158 |
| NZ_JAAOTI010000010.1 | Catellatospora chokoriensis 2-251 NODE_10 3e-09 | 89108:88951 | 88946:89244 |
| NZ_JAAOTJ010000004.1 | Catellatospora methionotrophica IMSNU 22006 NODE_4 3e-09 | 352877:352719 | 352593:353012 |
| NZ_BONH01000022.1 | Catellatospora citrea NBRC 14495 022 sequence 3e-08 | 89924:90081 | 89788:90087 |
| NZ_VCKX01000439.1 | Nonomuraea zeae DSM 100528 NODE_439_length_7696_cov_29.2159 3e-08 | 1039:1174 | 0:1182 |
| NZ_ML769302.1 | Catellatospora vulcania NEAU-JM1 Scaffold3 1e-07 | 189448:189600 | 189307:189605 |
| NZ_BOOO01000020.1 | Planotetraspora mira NBRC 15435 020 sequence 4e-07 | 242678:242825 | 242068:242936 |
| NZ_BOOD01000006.1 | Microbispora rosea subsp. rosea NBRC 14044 006 sequence 4e-07 | 273895:274037 | 273292:274162 |
| NZ_JAIWNB010000048.1 | Nonomuraea sp. NEAU-L178 Scaffold48 1e-06 | 33129:32983 | 32852:33834 |
| NZ_KB903293.1 | Catelliglobosispora koreensis DSM 44566 A3E1DRAFT_scaffold_9.10 5e-06 | 315055:315219 | 314854:315325 |
| NZ_AUAX01000003.1 | Hamadaea tsunoensis DSM 44101 G401DRAFT_scaffold00003.3_C 5e-06 | 370845:370690 | 370603:370936 |
| NZ_BONY01000143.1 | Rhizocola hellebori NBRC 109834 143 sequence 5e-06 | 12922:13054 | 12777:13100 |
| NZ_FOHX01000017.1 | Nonomuraea wenchangensis CGMCC 4.5598 5e-06 | 184370:184458 | 183749:184578 |
| NZ_SLWM01000007.1 | Kribbella sp. VKM Ac-2538 Ga0310562_107 2e-05 | 147268:147340 | 147077:147405 |
| NZ_KB894418.1 | Actinokineospora enzanensis DSM 44649 C503DRAFT_scaffold00016.16 9e-06 | 78126:77981 | 77717:78502 |
| NZ_JADBEA010000001.1 | Actinophytocola algeriensis DSM 46746 Ga0415166_01 0.0004 | 7296075:7296202 | 7295684:7296429 |
| NZ_VANP01000001.1 | Microbispora fusca NEAU-HEGS1-5 Scaffold1 0.0002 | 373038:373165 | 372395:373335 |
| NZ_SOCP01000022.1 | Actinophytocola oryzae DSM 45499 Ga0181036_122 8e-07 | 49761:49922 | 49268:50073 |
| NZ_PTIX01000012.1 | Actinokineospora auranticolor YU 961-1 Ga0180963_112 9e-06 | 47024:46879 | 46672:47313 |
| NZ_FNON01000002.1 | Amycolatopsis xylanica CPCC 202699 7e-14 | 430578:430306 | 430158:431047 |
| NZ_JAEIOJ010000015.1 | Umezawaea sp. REN6 NODE_15_length_111047_cov_230.772092 3e-18 | 47802:47558 | 47451:47908 |
| NZ_ATYT01000004.1 | Thermocrispum municipale DSM 44069 YWMDRAFT_scaffold_2.3_C 3e-06 | 310411:310249 | 310036:310695 |
| NZ_SMKA01000192.1 | Kribbella albertanoniae JCM 30547 NODE_192_length_18217_cov_30.6336 0.003 | 17031:16877 | 16844:17151 |
| NZ_JACHKF010000001.1 | Kribbella sandramycini DSM 15626 Ga0310417_01 0.003 | 1745501:1745438 | 1745410:1745702 |
| NZ_MTQN01000012.1 | Kribbella sp. ALI-6-A Contig16 0.003 | 676003:676064 | 675807:676088 |
| NZ_JAFJMJ010000001.1 | Amycolatopsis sp. 195334CR 1 9e-19 | 1949773:1949513 | 1949426:1950093 |
| NZ_FOKG01000004.1 | Amycolatopsis marina CGMCC 4.3568 3e-11 | 342345:342186 | 341917:342646 |
| NZ_MSIF01000014.1 | Actinophytocola xinjiangensis CGMCC 4.4663 contig14 0.009 | 69499:69444 | 69009:69697 |
| NZ_KI632509.1 | Haloechinothrix halophila YIM 93223 AmyhaDRAFT_Scaffold1.1 1e-11 | 1112802:1112958 | 1112446:1113193 |
| NZ_CP012752.1 | Kibdelosporangium phytohabitans KLBMP1111 0.009 | 375328:375278 | 374633:375556 |
| NZ_KE386604.1 | Thermocrispum agreste DSM 44070 YWYDRAFT_scaffold_1.2 2e-08 | 391827:392081 | 391547:392179 |
| NZ_FOGI01000007.1 | Actinokineospora terrae DSM 44260 2e-07 | 26027:26172 | 25727:26377 |
| NZ_SJKB01000009.1 | Kribbella pittospori NRRL B-24813 NODE_9_length_437662_cov_17.7996_ID_4276 0.031 | 164134:164090 | 164062:164322 |
| NZ_SLXQ01000001.1 | Tamaricihabitans halophyticus DSM 45765 Ga0310474_101 1e-09 | 838339:838177 | 837950:838487 |
| NZ_LAIR01000002.1 | Luteipulveratus halotolerans C296001 unitig_0_quiver_44 1e-31 | 214384:214478 | 214273:214960 |
| NZ_JABENB010000001.1 | Flexivirga aerilata ID2601S contig1 2e-15 | 396515:396426 | 395992:396637 |
| NZ_BMHI01000002.1 | Flexivirga endophytica CGMCC 1.15085 02 sequence 9e-13 | 643724:643813 | 643579:644378 |
| NZ_VIVQ01000002.1 | Rudaeicoccus suwonensis DSM 19560 Ga0104582_12 1e-12 | 81569:81476 | 81004:81689 |
| NZ_FOHB01000005.1 | Pedococcus cremeus CGMCC 1.6963 4e-11 | 87452:87368 | 86967:87540 |
| NZ_RJJQ01000023.1 | Flexivirga caeni BO-16 contig_23 4e-10 | 32924:33013 | 32804:33461 |
| NZ_JACHVQ010000002.1 | Flexivirga oryzae DSM 105369 Ga0310456_02 2e-09 | 431554:431464 | 431007:431677 |
| NZ_JAFDVE010000014.1 | Phycicoccus sp. CSK15P-2 Scaffold7_1 2e-08 | 202188:202270 | 202071:202717 |
| NZ_VCQV01000029.1 | Leekyejoonella antrihumi C5-26 contig29 2e-08 | 28963:28872 | 28450:29094 |
| NZ_VFOP01000001.1 | Ornithinicoccus hortensis DSM 12335 Ga0197519_11 7e-08 | 3675800:3675722 | 3675260:3675853 |
| NZ_JAAOIV010000010.1 | Metallococcus carri DB0510 contig10 2e-07 | 165105:165194 | 165053:165578 |
| NZ_LMSA01000002.1 | Knoellia sp. Soil729 contig_2 8e-07 | 708274:708187 | 707841:708379 |
| NZ_WIQI01000171.1 | Ornithinicoccus halotolerans EGI 80423 EGI_scaffold171 3e-06 | 795:847 | 754:1182 |
| NZ_JOEE01000001.1 | Phycicoccus jejuensis NRRL B-24460 contig1.1 3e-06 | 465807:465892 | 465704:466639 |
| NZ_JAHHWP010000025.1 | Phycicoccus sp. KQZ13P-1 Scaffold17_1 3e-06 | 18272:18187 | 17520:18374 |
| NZ_SEIP01000002.1 | Yimella sp. RIT 621 RIT621 2 4e-05 | 19602:19514 | 19140:19646 |
| NZ_JAFDVD010000014.1 | Phycicoccus sp. MQZ13P-5 Scaffold7_2 1e-05 | 85033:84947 | 84403:85166 |
| NZ_BCNQ01000005.1 | Piscicoccus intestinalis NBRC 104926 0.0001 | 10859:10775 | 10026:11064 |
| NZ_QDDJ01000001.1 | Ornithinimicrobium cavernae KCTC 49018 s.30183_Contig1 4e-05 | 69514:69471 | 68975:69593 |
| NZ_PVTI01000009.1 | Knoellia remsis ATCC BAA-1496 Ga0104721_109 0.001 | 74117:74169 | 74051:74575 |
| NZ_QTUA01000001.1 | Calidifontibacter indicus DSM 22967 Ga0197481_11 2e-09 | 976766:976853 | 976721:977292 |
| NZ_VFMO01000001.1 | Yimella lutea DSM 19828 Ga0197575_11 8e-07 | 791229:791317 | 791185:791690 |
| NZ_BAFE01000011.1 | Mobilicoccus pelagius NBRC 104925 0.0001 | 1292:1331 | 1112:2185 |
| NZ_VFQF01000003.1 | Humibacillus xanthopallidus DSM 21776 Ga0197590_13 0.018 | 927059:927140 | 926861:927586 |
| NZ_SWMA01000004.1 | Ornithinicoccus soli XNB-1 WT-5_scaffold4 8e-07 | 132166:132255 | 132037:132670 |
| NZ_JACVMY010000002.1 | Yimella sp. cx-51 Scaffold2_1 3e-06 | 284908:284820 | 284423:285024 |
| NZ_JAGEKR010000001.1 | Allobranchiibius sp. CTAmp26 CTAmp26_contig_1 9e-07 | 285104:285009 | 284654:285203 |
| NZ_JACCFW010000001.1 | Allobranchiibius huperziae DSM 29531 Ga0415215_01 0.0001 | 379418:379515 | 379320:379881 |
| NZ_JAGEKN010000005.1 | Allobranchiibius sp. GilTou38 GilTou38_contig_5 0.0001 | 283651:283554 | 283180:283749 |
| NZ_QEOM01000005.1 | Williamsia marianensis DSM 44944 Ga0215645_105 0 | 145043:145527 | 145042:145528 |
| NZ_PEBD01000004.1 | Williamsia marianensis BULT 1.1 Contig4 0 | 204894:204417 | 204389:204895 |
| NZ_RBKV01000001.1 | Williamsia muralis DSM 44343 Ga0197507_11 0 | 4418484:4418944 | 4418483:4418950 |
| NZ_BDAP01000001.1 | Williamsia muralis NBRC 105860 2e-07 | 686868:686827 | 686825:687292 |
| NZ_AYTE01000013.1 | Williamsia sp. D3 Contig0020 0 | 91818:92276 | 91817:92285 |
| NZ_MJEJ02000053.1 | Williamsia sp. 1138 288 7e-126 | 168205:167806 | 167760:168238 |
| NZ_QJSP01000007.1 | Williamsia limnetica DSM 45521 Ga0244495_107 9e-125 | 142903:143304 | 142874:143387 |
| NZ_JAFRDR010000017.1 | Williamsia sp. C17 Scaffold17_1 9e-125 | 42302:41960 | 41910:42391 |
| NZ_MJEI01000104.1 | Williamsia sp. 1135 contig603 2e-119 | 39073:38731 | 38681:39163 |
| NZ_JAHCSG010000003.1 | Williamsia sp. CHRR-6 contig00003 8e-37 | 298669:298965 | 298653:299017 |
| NZ_BMCS01000001.1 | Williamsia phyllosphaerae CCM 7855 1 sequence 1e-34 | 330162:330462 | 330101:330558 |
| NZ_JXYP01000010.1 | Williamsia herbipolensis ARP1 contig5 1e-28 | 170913:170616 | 170527:170973 |
| NZ_LMPL01000001.1 | Williamsia sp. Leaf354 contig_1 1e-28 | 879311:879014 | 878926:879380 |
| NZ_JACBYW010000007.1 | Actinopolyspora biskrensis CECT 8576 Ga0372422_07 0 | 209700:210354 | 209678:210355 |
| NZ_AQUI01000002.1 | Actinopolyspora halophila DSM 43834 ActhaDRAFT_contig1.1_C 0 | 453718:454375 | 453696:454379 |
| NZ_FNKO01000002.1 | Actinopolyspora saharensis DSM 45459 0 | 1196359:1195829 | 1195716:1196381 |
| NZ_JAANTE010000011.1 | Actinopolyspora sp. BKK2 NODE_11_length_185776_cov_67.838491 0 | 58658:58096 | 57996:58680 |
| NZ_PVSR01000015.1 | Actinopolyspora mortivallis M5A NODE_15_length_74256_cov_26.5436_ID_2083 2e-95 | 66122:66399 | 65882:66457 |
| NZ_KB913024.1 | Actinopolyspora mortivallis DSM 44261 HS-1 ActmoDRAFT_scaffold1.1 9e-95 | 3600743:3600465 | 3600409:3600983 |
| NZ_FPAT01000012.1 | Actinopolyspora righensis DSM 45501 4e-80 | 62329:62077 | 61928:62560 |
| NZ_FNFM01000006.1 | Actinopolyspora mzabensis DSM 45460 4e-80 | 315805:316057 | 315534:316204 |
| NZ_FNJR01000017.1 | Actinopolyspora xinjiangensis DSM 46732 2e-76 | 26702:26978 | 26321:27070 |
| NZ_FOMZ01000004.1 | Actinopolyspora alba DSM 45004 3e-75 | 328998:329248 | 328768:329414 |
| NZ_FOME01000010.1 | Saccharopolyspora kobensis CGMCC 4.3529 2e-45 | 43270:42983 | 42899:43476 |
| NZ_VWPH01000012.1 | Saccharopolyspora hirsuta VKM Ac-666 n12 8e-45 | 241318:241026 | 240943:241466 |
| NZ_RBXX01000002.1 | Saccharopolyspora antimicrobica DSM 45119 Ga0074777_12 1e-42 | 2302737:2302450 | 2302367:2302944 |
| NZ_VFPI01000007.1 | Halopolyspora algeriensis DSM 46680 Ga0314185_17 5e-41 | 98079:98357 | 97961:98443 |
| NZ_FOZX01000002.1 | Saccharopolyspora flava DSM 44771 2e-40 | 758839:758579 | 758533:759060 |
| NZ_RSAA01000009.1 | Saccharopolyspora rhizosphaerae H219 scaffold9_cov25 2e-39 | 10893:11180 | 10740:11224 |
| NZ_JACCFJ010000001.1 | Saccharopolyspora hordei DSM 44065 Ga0197497_01 2e-39 | 406926:406640 | 406575:407202 |
| NZ_SMKV01000036.1 | Saccharopolyspora aridisoli 16K404 NODE_36_length_54625_cov_61.1191 2e-39 | 43588:43341 | 43297:43851 |
| NZ_SMKW01000013.1 | Saccharopolyspora elongata 7K502 NODE_13_length_153421_cov_107.977 2e-39 | 15311:15588 | 15031:15684 |
| NZ_SMKS01000078.1 | Saccharopolyspora terrae 16K309 NODE_78_length_24311_cov_47.6441 7e-39 | 13355:13110 | 13066:13621 |
| NZ_JAGPXE010000016.1 | Saccharopolyspora endophytica KCTC 19397 Saccharopolyspora_endophytica_contig_16 3e-37 | 166924:166679 | 166635:167148 |
| NZ_SMLA01000023.1 | Saccharopolyspora karakumensis 5K548 NODE_23_length_83782_cov_27.5581 3e-37 | 74398:74153 | 74109:74643 |
| NZ_CP054839.1 | Saccharopolyspora erythraea SCSIO 07745 3e-37 | 400239:400515 | 399868:400593 |
| NC_009142.1 | Saccharopolyspora erythraea NRRL 2338 1e-36 | 402098:402348 | 401079:402437 |
| NZ_JACHIW010000001.1 | Saccharopolyspora phatthalungensis DSM 45584 Ga0436922_01 1e-35 | 1705494:1705218 | 1705111:1705776 |
| NZ_BMMT01000001.1 | Saccharopolyspora subtropica CGMCC 4.7206 01 sequence 5e-34 | 9945:10222 | 9657:10298 |
| NZ_FNOK01000020.1 | Saccharopolyspora shandongensis CGMCC 4.3530 5e-34 | 135242:134974 | 134892:135532 |
| NZ_JACGWZ010000008.1 | Saccharopolyspora lacisalsi DSM 45975 Ga0197584_08 3e-30 | 3786:3509 | 3462:3984 |
| NZ_JACHIV010000001.1 | Saccharopolyspora gloriosae DSM 45582 Ga0436921_01 4e-29 | 6187801:6187547 | 6187488:6188165 |
| NZ_FQVN01000001.1 | Streptoalloteichus hindustanus DSM 44523 8e-26 | 946809:947085 | 946511:947214 |
| NZ_JADDUE010000001.1 | Saccharopolyspora sp. HNM0986 NODE_1_length_979558_cov_78.761708_cov_78.761708 9e-25 | 444416:444670 | 444090:444745 |
| NZ_JAIUKC010000139.1 | Saccharopolyspora sp. 7B contig_00139 1e-23 | 22332:22589 | 22109:22659 |
| NZ_SGWQ01000003.1 | Herbihabitans rhizosphaerae DSM 101727 Ga0310478_103 1e-22 | 595309:595052 | 594902:595529 |
| NZ_JNVU01000037.1 | Saccharopolyspora rectivirgula DSM 43113 contig00038 2e-21 | 221665:221887 | 221361:221938 |
| NZ_JAHXGQ010000006.1 | Saccharothrix sp. SC076 6 2e-20 | 424407:424626 | 424101:424757 |
| NZ_RBXR01000001.1 | Saccharothrix variisporea DSM 43911 Ga0197496_11 7e-20 | 4974622:4974383 | 4974272:4974786 |
| NZ_JNXC01000026.1 | Saccharothrix sp. NRRL B-16314 contig26.1 2e-19 | 23390:23151 | 23040:23490 |
| NZ_JAFBCL010000001.1 | Saccharothrix algeriensis DSM 44581 Ga0451104_01 9e-19 | 3017689:3017450 | 3017339:3017874 |
| NZ_JACHMO010000001.1 | Saccharothrix ecbatanensis DSM 45486 Ga0374009_01 1e-17 | 7778383:7778622 | 7778017:7778733 |
| NZ_CP073318.1 | Kutzneria sp. CA-103260 1e-17 | 8489695:8489458 | 8489363:8489901 |
| NZ_LGED01000057.1 | Saccharothrix sp. NRRL B-16348 P442contig15.1 1e-16 | 23426:23665 | 23306:23776 |
| NZ_KK037166.1 | Kutzneria sp. 744 supercont1.1 1e-16 | 5549385:5549172 | 5549053:5549594 |
| NZ_JACHMH010000001.1 | Crossiella cryophila DSM 44230 Ga0197469_01 1e-16 | 1454168:1454440 | 1454055:1454537 |
| NZ_VFPP01000001.1 | Saccharothrix saharensis DSM 45456 Ga0264273_11 2e-15 | 4525735:4525496 | 4525385:4526087 |
| NZ_JACHIR010000001.1 | Kutzneria kofuensis DSM 43851 Ga0436916_01 2e-15 | 3843611:3843392 | 3843280:3843808 |
| NZ_QUNO01000004.1 | Kutzneria buriramensis DSM 45791 Ga0104555_104 5e-15 | 445053:445278 | 444834:445373 |
| NZ_PVNH01000002.1 | Prauserella shujinwangii CGMCC 4.7125 Ga0171606_102 5e-15 | 683515:683261 | 683102:683675 |
| NZ_CP015163.1 | Amycolatopsis albispora WP1 . 5e-15 | 5804036:5803776 | 5803687:5804356 |
| NZ_LWLC01000004.1 | Saccharothrix sp. CB00851 scaffold12 5e-15 | 39493:39684 | 39159:39795 |
| NZ_MUMH01000080.1 | Allokutzneria sp. NRRL B-24872 NRRL_B-24872_contig_80 5e-15 | 7821:7546 | 7447:8206 |
| NZ_RBXO01000001.1 | Saccharothrix australiensis DSM 43800 Ga0197483_11 2e-14 | 638526:638738 | 638300:638876 |
| NZ_VUOB01000022.1 | Goodfellowiella sp. AN110305 contig_22 2e-14 | 136481:136761 | 136167:136899 |
| NZ_VKAB01000017.1 | Saccharothrix deserti BMP B8144 Scaffold12_1 7e-14 | 66350:66591 | 65988:66702 |
| NZ_PYAX01000005.1 | Saccharothrix carnea CGMCC 4.7097 Ga0171604_105 2e-13 | 613120:613360 | 612834:613471 |
| NZ_JNYZ01000021.1 | Amycolatopsis jejuensis NRRL B-24427 contig21.1 2e-13 | 108843:108683 | 108471:109058 |
| NZ_JAGGMS010000001.1 | Amycolatopsis magusensis DSM 45510 Ga0197551_01 2e-13 | 857617:857353 | 857269:857934 |
| NZ_JAAOYM010000001.1 | Saccharomonospora amisosensis DSM 45685 Ga0310452_01 8e-13 | 389948:390133 | 389793:390324 |
| NZ_JADQDF010000001.1 | Pseudonocardia oceani KRD185 1 8e-13 | 2621287:2621480 | 2621183:2621516 |
| NZ_MTQO01000048.1 | Actinosynnema sp. ALI-1.44 Contig52 8e-13 | 436798:436551 | 436415:437220 |
| NZ_JAAATY010000009.1 | Kibdelosporangium persicum 4NS15 _09 sequence 3e-12 | 152683:152522 | 152292:153462 |
| NZ_VIWU01000001.1 | Pseudonocardia hierapolitana DSM 45671 Ga0197558_11 3e-12 | 3013592:3013782 | 3013402:3013863 |
| NZ_JACHMG010000001.1 | Amycolatopsis jiangsuensis DSM 45859 Ga0104640_01 3e-12 | 5701174:5701448 | 5702348:5701563 |
| NZ_MTQP01000068.1 | Saccharothrix sp. ALI-22-I Contig72 3e-12 | 184836:184601 | 184485:185199 |
| NZ_JAGINV010000001.1 | Saccharothrix coeruleofusca DSM 43679 Ga0451103_01 3e-12 | 7005573:7005810 | 7005213:7005910 |
| NZ_CP053564.1 | Pseudonocardia broussonetiae Gen 01 3e-11 | 1179733:1179916 | 1179616:1179960 |
| NZ_PDJK01000002.1 | Amycolatopsis sulphurea DSM 46092 Ga0074782_12 3e-11 | 2882430:2882705 | 2881538:2882821 |
| NZ_QHKI01000047.1 | Kibdelosporangium aridum A82846 AA000374-47_GDW01298 3e-11 | 20039:19860 | 19628:20441 |
| NZ_CP080521.1 | Pseudonocardia sp. DSM 110487 3e-11 | 480820:480630 | 480549:480918 |
| NZ_VFPH01000003.1 | Pseudonocardia cypriaca DSM 45511 Ga0310437_13 1e-10 | 1535043:1534868 | 1534770:1535136 |
| NZ_CP007155.1 | Kutzneria albida DSM 43870 1e-10 | 328075:328261 | 327985:328452 |
| NZ_CM001439.1 | Saccharomonospora marina XMU15 1e-10 | 5198969:5198788 | 5198593:5199200 |
| NZ_CP069526.1 | Amycolatopsis sp. FDAARGOS 1241 FDAARGOS_1241 1e-10 | 44588:44768 | 44421:44979 |
| NZ_CP080519.1 | Amycolatopsis sp. DSM 110486 1e-10 | 3052843:3052663 | 3052451:3052995 |
| NZ_VFPA01000004.1 | Pseudonocardia kunmingensis DSM 45301 Ga0264272_104 1e-10 | 656456:656647 | 656361:656738 |
| NZ_JAGIOO010000001.1 | Crossiella equi DSM 44580 Ga0451160_01 1e-10 | 4557952:4557749 | 4557567:4558066 |
| NZ_CM001440.1 | Saccharomonospora cyanea NA-134 1e-10 | 4946163:4945880 | 4945848:4946300 |
| NZ_FOEF01000023.1 | Amycolatopsis saalfeldensis DSM 44993 4e-10 | 8973:8792 | 8581:9576 |
| NZ_FWXV01000002.1 | Kibdelosporangium aridum DSM 43828 4e-10 | 1798389:1798546 | 1798086:1798778 |
| NZ_JAEHOB010000004.1 | Amycolatopsis sp. YIM S01255 Scaffold4 4e-10 | 491671:491403 | 491217:491972 |
| NZ_SDLT01000007.1 | Amycolatopsis nivea CFH S0261 Scaffold7 4e-10 | 217235:217056 | 216854:217486 |
| NZ_BMMK01000019.1 | Longimycelium tulufanense CGMCC 4.5737 019 sequence 4e-10 | 68180:67945 | 67873:68749 |
| NZ_CP034550.1 | Saccharothrix syringae NRRL B-16468 4e-10 | 317685:317924 | 317453:318024 |
| NZ_CP024894.1 | Amycolatopsis sp. AA4 4e-10 | 7907045:7906866 | 7906664:7907296 |
| NZ_QZFV01000063.1 | Amycolatopsis panacis YIM PH21725 NODE_18 1e-09 | 46:269 | 0:385 |
| NZ_JACHJN010000004.1 | Saccharothrix tamanrassetensis CECT 8640 Ga0436953_04 1e-09 | 698895:698682 | 698543:699412 |
| NZ_FOWW01000016.1 | Amycolatopsis arida CGMCC 4.5579 1e-09 | 11838:11685 | 11428:13011 |
| NZ_NKYE01000005.1 | Amycolatopsis antarctica AU-G6 NODE_5_length_372561_cov_254.23 1e-09 | 338753:338983 | 338378:339132 |
| NZ_NKYF01000003.1 | Pseudonocardia sp. MH-G8 Scaffold3 1e-09 | 4160:4354 | 4059:4445 |
| NZ_BJNG01000040.1 | Pseudonocardia hydrocarbonoxydans NBRC 14498 40 sequence 5e-09 | 55469:55660 | 55350:55696 |
| NZ_QLTT01000012.1 | Lentzea atacamensis DSM 45479 Ga0215649_112 5e-09 | 199481:199336 | 199046:199677 |
| NZ_FNBE01000012.1 | Pseudonocardia oroxyli CGMCC 4.3143 5e-09 | 97813:98062 | 97765:98147 |
| NZ_JAGINW010000001.1 | Kibdelosporangium banguiense DSM 46670 Ga0451105_01 5e-09 | 1896160:1895979 | 1895747:1896424 |
| NZ_CP076538.1 | Amycolatopsis sp. YIM 96748 5e-09 | 583519:583768 | 583049:583949 |
| NZ_CP060131.1 | Pseudonocardia sp. CGMCC 4.1532 5e-09 | 1257892:1258083 | 1257783:1258119 |
| NZ_CABVGP010000003.1 | Amycolatopsis sp. A23 isolate AA231_1 5e-09 | 350684:350524 | 350302:351014 |
| NZ_FZNW01000012.1 | Haloechinothrix alba DSM 45207 5e-09 | 115937:115664 | 115559:115959 |
| NZ_VFML01000001.1 | Amycolatopsis cihanbeyliensis DSM 45679 Ga0197559_11 5e-09 | 4640283:4640539 | 4639900:4640720 |
| NZ_FMZZ01000019.1 | Actinokineospora iranica IBRC-M 10403 2e-08 | 11489:11727 | 11159:11875 |
| NZ_SLWS01000001.1 | Actinocrispum wychmicini DSM 45934 Ga0310477_101 2e-08 | 58670:58516 | 58286:58990 |
| NZ_JAANOU010000001.1 | Amycolatopsis viridis DSM 45668 Ga0197555_01 2e-08 | 625102:625349 | 624912:625401 |
| NZ_JAFBCX010000002.1 | Lentzea nigeriaca DSM 45680 Ga0453997_02 2e-08 | 435727:435872 | 435530:436161 |
| NZ_KB912942.1 | Amycolatopsis benzoatilytica AK 1665 AmybeDRAFT_scaffold1.1 2e-08 | 7647764:7647920 | 7647092:7648123 |
| NZ_ARVW01000001.1 | Amycolatopsis nigrescens CSC17Ta-90 AmyniDRAFT_Contig68.1_C 2e-08 | 4100326:4100573 | 4100018:4100710 |
| NZ_JAANOV010000001.1 | Amycolatopsis granulosa DSM 45669 Ga0197595_01 6e-08 | 3705914:3706160 | 3705723:3706212 |
| NZ_FOWC01000001.1 | Amycolatopsis rubida DSM 44637 6e-08 | 1494568:1494726 | 1494300:1494928 |
| NZ_JADQDK010000001.1 | Pseudonocardia abyssalis KRD168 contig_1_pilon 6e-08 | 4563475:4563674 | 4563349:4563702 |
| NZ_QFWW01000004.1 | Lentzea terrae NEAU-LZS 42 scaffold4 6e-08 | 178119:178261 | 177923:178554 |
| NZ_PQHX01000060.1 | Amycolatopsis thermalba 50.9b NODE_60 6e-08 | 1391:1238 | 1076:1581 |
| NZ_PQHZ01000049.1 | Amycolatopsis palatopharyngis DSM 44832 NODE_49 6e-08 | 35455:35296 | 35025:35846 |
| NZ_KE387043.1 | Saccharomonospora iraqiensis IQ-H1 ActirDRAFT_Scaffold112.91 2e-07 | 1738:1512 | 1481:2045 |
| NZ_CM001484.1 | Saccharomonospora glauca K62 2e-07 | 4143299:4143073 | 4143017:4143489 |
| NZ_JACBJG010000012.1 | Saccharomonospora sp. NB11 NODE_12_length_144702_cov_58.687 2e-07 | 21071:20763 | 20724:21186 |
| NZ_JAENJH010000002.1 | Prauserella sp. ASG 168 NODE_2_length_1331183_cov_33.8405 2e-07 | 1147445:1147564 | 1147232:1147834 |
| NZ_FNCC01000018.1 | Lentzea fradiae CGMCC 4.3506 2e-07 | 67690:67546 | 67256:67942 |
| NZ_FPJG01000006.1 | Amycolatopsis australiensis DSM 44671 2e-07 | 1558707:1558866 | 1558432:1559088 |
| NZ_AHBX01000099.1 | Saccharomonospora azurea SZMC 14600 contig99 2e-07 | 25037:24749 | 24687:25182 |
| NZ_JAAMPJ010000002.1 | Lentzea alba NEAU-D13 Scaffold2 8e-07 | 700176:700036 | 699741:700374 |
| NZ_BNAU01000004.1 | Amycolatopsis deserti CGMCC 4.7677 04 sequence 8e-07 | 501665:501512 | 501350:501874 |
| NZ_CM001466.1 | Saccharomonospora azurea NA-128 8e-07 | 2565890:2565601 | 2565539:2566035 |
| NZ_KI912258.1 | Saccharomonospora iraqiensis subsp. paurometabolica YIM 90007 SacpaDRAFT_SPI.75 8e-07 | 105196:105422 | 104889:105453 |
| NZ_WHOL01000065.1 | Actinokineospora pegani TRM 65233 815 3e-06 | 59985:60126 | 59786:60336 |
| NZ_WMBA01000131.1 | Amycolatopsis pithecellobii RM579 NODE_131_length_3422_cov_8.983308 3e-06 | 5:127 | 0:0 |
| NZ_VTHK01000018.1 | Amycolatopsis anabasis EGI 650086 RDPYD18112716_A.Scaf18 3e-06 | 65205:64961 | 64760:65712 |
| NZ_VDFW01000037.1 | Amycolatopsis alkalitolerans SYSUP0005 Amycolatopsis_sp.strain_SYSUP0005_Contig37 3e-06 | 8300:8457 | 8057:8614 |
| NZ_JACHJS010000001.1 | Saccharothrix violaceirubra DSM 45084 Ga0436958_01 3e-06 | 6746550:6746310 | 6746209:6746688 |
| NZ_CP016793.1 | Lentzea guizhouensis DHS C013 9e-06 | 2554033:2553889 | 2553599:2554309 |
| NC_015312.1 | Pseudonocardia dioxanivorans CB1190 9e-06 | 448221:448045 | 447912:448397 |
| NZ_BEGX01000032.1 | Pseudonocardia sp. N23 9e-06 | 69941:70091 | 69867:70302 |
| NZ_JACCFK010000001.1 | Amycolatopsis endophytica DSM 104006 Ga0374050_01 9e-06 | 2337170:2337323 | 2336977:2337485 |
| NZ_SNXU01000001.1 | Actinokineospora alba DSM 45114 Ga0197470_11 9e-06 | 2023833:2023971 | 2023614:2024225 |
| NZ_QQAU01000014.1 | Lentzea flaviverrucosa DSM 44664 Ga0244493_114 3e-05 | 148490:148350 | 148054:148686 |
| NZ_BMNC01000005.1 | Lentzea pudingi CGMCC 4.7319 05 sequence 3e-05 | 297255:297115 | 296819:297432 |
| NZ_MUYM01000021.1 | Lentzea kentuckyensis NRRL B-24416 NRRL_B-24416_contig_21 3e-05 | 22096:22236 | 21899:22531 |
| NZ_AXWW01000027.1 | Actinokineospora inagensis DSM 44258 H504DRAFT_scaffold00022.22_C 3e-05 | 52659:52527 | 52332:53019 |
| NZ_VMNW02000140.1 | Amycolatopsis acidicola K81G1 NODE_140_length_15511_cov_23.1871 3e-05 | 6656:6502 | 6346:6870 |
| NZ_FNUJ01000004.1 | Amycolatopsis pretoriensis DSM 44654 3e-05 | 578625:578785 | 578302:579007 |
| NZ_MASU01000011.1 | Prauserella sp. YIM 121212 BA062_c11 3e-05 | 212497:212591 | 212330:212858 |
| NZ_CP016353.1 | Prauserella marina DSM 45268 0.0001 | 6319344:6319188 | 6318982:6319527 |
| NZ_AFWY03000027.1 | Amycolatopsis sp. ATCC 39116 Amy_contig00089.27_C 0.0001 | 88274:88429 | 88069:88591 |
| NZ_JAHDTG010000026.1 | Pseudonocardia sp. H11422 Scaffold23_1 0.0001 | 53474:53667 | 53362:53829 |
| NZ_VOBR01000008.1 | Lentzea sp. FXJ1.1311 Scaffold8 0.0001 | 70270:70126 | 69844:70508 |
| NZ_JABVED010000003.1 | Actinokineospora xionganensis HBU206404 Scaffold3 0.0004 | 243338:243476 | 243121:243730 |
| NZ_BJVJ01000132.1 | Pseudonocardia sulfidoxydans NBRC 16205 132 sequence 0.0004 | 7267:7158 | 6958:7368 |
| NZ_JNYY01000006.1 | Amycolatopsis vancoresmycina NRRL B-24208 contig6.1 0.0004 | 468965:468807 | 468586:469404 |
| NZ_LN850107.1 | Alloactinosynnema sp. L-07 isolate Alloactinosynnema sp. L-07 I 0.0004 | 6194971:6195109 | 6194735:6195363 |
| NZ_MASW01000001.1 | Prauserella muralis DSM 45305 BAY60_c01 0.001 | 394783:394603 | 394398:394987 |
| NZ_AUII01000023.1 | Pseudonocardia asaccharolytica DSM 44247 _ NBRC 16224 DSM 44247 G567DRAFT_scaffold00022.22_C 0.001 | 25377:25187 | 25056:25469 |
| NZ_QHCP01000002.1 | Actinokineospora mzabensis CECT 8578 Ga0244608_102 0.001 | 768252:768159 | 767950:768646 |
| NZ_FORP01000033.1 | Amycolatopsis sacchari DSM 44468 0.001 | 49391:49237 | 49081:49592 |
| NZ_AP022617.1 | Mycolicibacterium monacense JCM 15658 0.001 | 5535868:5535917 | 5535561:5535966 |
| NZ_JAFB01000015.1 | Amycolatopsis taiwanensis DSM 45107 A3CUDRAFT_scaffold_11.12_C 0.005 | 125064:125189 | 124788:125367 |
| NZ_SMFZ01000001.1 | Pseudonocardia endophytica DSM 44969 Ga0197528_11 0.005 | 3206065:3206172 | 3205977:3206320 |
| NZ_AZUM01000002.1 | Saccharomonospora piscinae CNQ490 B126DRAFT_scaffold_1.2_C 0.005 | 805714:805519 | 805461:805933 |
| NZ_BNAR01000009.1 | Lentzea cavernae CGMCC 4.7367 09 sequence 0.005 | 300247:300344 | 300004:300636 |
| NZ_MKQR01000028.1 | Actinokineospora bangkokensis 44EHW Scaffold5 0.005 | 66836:66928 | 66428:67139 |
| NZ_VJWX01000860.1 | Amycolatopsis rhizosphaerae TBRC 6029 NODE_860_length_1280_cov_3.78057 0.005 | 4:96 | 0:0 |
| NC_013159.1 | Saccharomonospora viridis DSM 43017 0.005 | 3917111:3916926 | 3916855:3917461 |
| NZ_PPHG01000094.1 | Amycolatopsis sp. CA-128772 Contig0000094 0.005 | 98842:99000 | 98320:99221 |
| NZ_JNYD01000020.1 | Pseudonocardia autotrophica NRRL B-16064 contig20.1 0.005 | 23575:23478 | 23285:23678 |
| NZ_BNAV01000010.1 | Amycolatopsis bartoniae CGMCC 4.7679 10 sequence 0.017 | 175116:175011 | 174854:175469 |
| NZ_JAAXKZ010000007.1 | Pseudonocardia bannensis DSM 45300 NODE_7_length_92078_cov_21.806821 0.017 | 53988:53889 | 53632:54103 |
| NZ_JAAXLS010000078.1 | Amycolatopsis acididurans K13G38 NODE_78_length_22348_cov_44.065794 0.017 | 8635:8724 | 8406:8889 |
| NZ_AUBB01000026.1 | Pseudonocardia spinosispora DSM 44797 G406DRAFT_scaffold00015.15_C 0.017 | 142494:142683 | 142371:142782 |
| NZ_SWMS01000009.1 | Prauserella endophytica CGMCC 4.7182 Scaffold9 0.017 | 146523:146617 | 146356:146883 |
| NZ_KI632511.1 | Actinospica robiniae DSM 44927 ActroDRAFT_ARA.1 0 | 5654749:5655161 | 5654748:5655162 |
| NZ_JAGSOG010000136.1 | Actinospica durhamensis CSCA 57 NODE_136_length_22758_cov_33.3361 5e-108 | 21648:21301 | 21237:21898 |
| NZ_JAGSOH010000013.1 | Actinospica sp. MGRD01-02 NODE_13_length_91938_cov_43.6122 6e-69 | 51276:50977 | 50951:51525 |
| NZ_JAGSXH010000015.1 | Actinocrinis puniceicyclus DSM 45618 NODE_15_length_86014_cov_13.3141 2e-11 | 53547:53417 | 53264:53732 |
| NZ_PGGW01000058.1 | Streptomyces carminius TRM SA0054 502 0.003 | 174328:174266 | 174107:174491 |
| NZ_WIXO01000001.1 | Streptomyces taklimakanensis TRM43335 Contig00001 0.003 | 3208090:3208152 | 3207908:3208413 |
| NZ_AZWL01000006.1 | Streptomyces sp. CNH099 B121DRAFT_scaffold_4.5_C 0.01 | 90885:90947 | 90717:91132 |
| NZ_BIFH01000023.1 | Embleya hyalina NBRC 13850 23 sequence 1e-109 | 97357:97678 | 97271:97790 |
| NZ_CP048261.1 | Streptomyces rimosus subsp. rimosus ATCC 10970 0.036 | 7086821:7086865 | 7086658:7086865 |
| NZ_JADEYH010000041.1 | Streptomyces verrucosisporus CPB1-1 41 0.036 | 6095:6157 | 5934:6384 |
| NZ_WWJX01000336.1 | Streptomyces sp. SID3343 SID3343.c401 6e-88 | 3126:2820 | 2758:3185 |
| NZ_NWVL01000001.1 | Streptomyces sp. WZ.A104 Scaffold1 0.0009 | 170946:171014 | 170675:171106 |
| NZ_WWJL01000018.1 | Streptomyces sp. SID4921 SID4921.c18 0.038 | 599233:599301 | 598932:599381 |

References

1. Bernhart SH, Hofacker IL, Will S, et al. RNAalifold: improved consensus structure prediction for RNA alignments. BMC Bioinformatics. 2008;9:474.

2. Larkin MA, Blackshields G, Brown NP, et al. Clustal W and clustal X version 2.0. Bioinformatics. 2007 Nov 1;23(21):2947-2948.
